# Novel and conserved drought-responsive microRNAs expression analysis in root tissues of wheat (*Triticum asetivum* L.) at reproductive stage

**DOI:** 10.3389/fpls.2025.1581542

**Published:** 2025-05-20

**Authors:** Pradeep Sharma, Shefali Mishra, Amandeep Kaur, O. P. Ahlawat, Ratan Tiwari

**Affiliations:** Crop Improvement Division, ICAR-Indian Institute of Wheat and Barley Research, Karnal, India

**Keywords:** wheat, miRNA, transcripts, abiotic stress, drought, DEG, reproductive stage

## Abstract

**Introduction:**

MicroRNAs (miRNAs) are a class of 20- to 24-nucleotide endogenous small RNAs that regulate gene expression post-transcriptionally, playing vital roles in plant development and stress responses. Among abiotic stresses, drought stress (DS) is one of the most critical factors affecting wheat yield worldwide. Understanding miRNA-mediated regulatory mechanisms under drought stress conditions is crucial for improving drought tolerance in wheat.

**Methods:**

To identify drought-responsive miRNAs in wheat, small RNA libraries were constructed from drought-tolerant (NI5439) and drought-susceptible (WL711) genotypes subjected to both control and drought-stress conditions. High-throughput sequencing was used to identify known and novel miRNAs. The family distribution of miRNAs, target prediction, pathway analysis, and differential expression analysis were conducted. A heat map was generated for the top 50 up- and downregulated miRNAs, and novel miRNAs were validated through qRT-PCR.

**Results and discussion:**

A total of 306 known and 58 novel miRNAs were identified across the two wheat genotypes. The identified miRNAs belonged to over 18 families, with miR9662a-3p being the most abundant. Most identified miRNAs were 21 nucleotides in length. A total of 2,300 target genes were predicted for the known miRNAs. Pathway analysis revealed that target genes were involved in key biological processes including signal transduction, transport, organelle localization, DNA methylation, histone and chromatin modification, and plant development. Ten novel miRNAs were validated using qRT-PCR, confirming their differential expression under drought stress. The findings significantly expand the repertoire of drought-responsive and novel miRNAs in wheat. These miRNAs and their target genes provide valuable insights into the molecular mechanisms underlying drought tolerance. The validated novel miRNAs represent potential targets for genetic manipulation to enhance drought resilience in wheat cultivars.

**Conclusion:**

This study provides a comprehensive miRNA expression profile in wheat under drought conditions and highlights several novel miRNAs that are differentially expressed between tolerant and susceptible genotypes. The integration of sequencing, computational analysis, and qRT-PCR validation strengthens the utility of these findings for future functional genomics studies and breeding programs aimed at developing drought-tolerant wheat varieties.

## 1 Introduction

Climate change, primarily driven by global warming, has emerged as a significant threat to ecosystems and food security worldwide. Rising temperatures, erratic precipitation patterns, and increased frequency of extreme weather events have intensified challenges in global agriculture, necessitating urgent strategies for climate-resilient food production. Wheat (*Triticum aestivum L.*), one of the most widely cultivated cereal crops, serves as a fundamental source of calories and nutrition for billions of people. However, despite advancements in wheat production over the past decade, global consumption has outpaced supply, exacerbating the demand-supply gap (Parmar et al., 2020; Kaur et al., 2023a; Zhao et al., 2024).

Drought stress at the reproductive stage poses a significant threat to wheat productivity by disrupting key physiological and molecular processes that are essential for grain development. It induces oxidative stress due to excessive accumulation of reactive oxygen species (ROS), leading to cellular damage, impaired photosynthetic efficiency, and premature leaf senescence during the grain-filling stage, ultimately reducing biomass accumulation and grain yield (Nelson et al., 2014; Kim et al., 2020; Berahim et al., 2021; Ahmad et al., 2023). To mitigate these detrimental effects, breeding programs have focused on developing drought-tolerant wheat varieties with enhanced physiological adaptability and stress-responsive molecular mechanisms (Wang et al., 2023). However, the intricate nature of drought responses necessitates a deeper understanding of the regulatory pathways involved in stress adaptation.Phytohormones play a crucial role in modulating wheat’s response to drought stress at the reproductive stage. Abscisic acid (ABA) and jasmonic acid (JA) regulate stomatal closure to minimize water loss while activating stress-responsive gene networks through the mitogen-activated protein kinase (MAPK) signaling pathway (Sharma et al., 2023). Additionally, JA influences ABA biosynthesis and degradation, creating a dynamic hormonal interplay that is critical for drought adaptation (Wang et al., 2022). However, excessive accumulation of ABA and JA can accelerate leaf senescence, impairing photosynthetic activity and reducing overall grain yield (Liu et al., 2023). In contrast, brassinosteroids (BRs) contribute to osmotic regulation, while cytokinins (CTKs) delay senescence and counteract the negative effects of ABA and JA, thereby enhancing stress resilience in wheat (Chen et al., 2023; Sun et al., 2023). Understanding these complex hormonal interactions is essential for developing strategies to improve wheat’s reproductive-stage drought tolerance and ensure sustainable yield production under water-limited conditions.

Recent advancements in molecular biology have highlighted the role of microRNAs (miRNAs) as key post-transcriptional regulators in plant stress responses. miRNAs are small, non-coding RNA molecules that regulate gene expression by targeting specific mRNAs for degradation or translational repression. Emerging evidence suggests that miRNAs play a crucial role in modulating drought tolerance by influencing stress-responsive pathways such as transcriptional regulation, hormone signaling, and antioxidant defense mechanisms (Sunkar et al., 2012a; Liu et al., 2023). High-throughput sequencing technologies have facilitated the identification of numerous stress-inducible miRNAs in wheat, including miR156, miR166, miR169, miR172, and miR399, which target key genes involved in stress adaptation (Ramachandran et al., 2020; Saroha et al., 2024). Despite growing knowledge of miRNA-mediated stress regulation, the molecular mechanisms underlying miRNA-mRNA interactions in wheat drought responses remain largely unexplored. Functional enrichment analyses and genome-wide expression studies suggest that miRNAs play a significant role in coordinating complex regulatory networks to enhance drought resilience (Mishra et al., 2023a). However, a comprehensive understanding of these regulatory pathways is still lacking, particularly during the critical grain-filling stage when drought stress has the most profound impact on yield formation.

This study aims to elucidate the role of drought-responsive miRNAs in wheat by identifying key miRNA-mRNA regulatory networks involved in drought adaptation. By integrating high-throughput sequencing, transcriptome analysis, and functional validation approaches, we seek to uncover novel miRNA-mediated mechanisms that contribute to drought tolerance in wheat. The findings of this research will provide valuable insights for breeding climate-resilient wheat varieties with enhanced drought tolerance, ensuring sustainable wheat production in the face of global climate change.

## 2 Materials and methods

### 2.1 Stress treatment, tissue collection, and root phenotyping

The study utilized two contrasting wheat genotypes for drought stress, NI5439 as tolerant (T) and WL711 as susceptible (S), to evaluate their performance under control (C) and drought stress (D) conditions (Kaur et al., 2017). Both genotypes were grown in cylindrical mud pots measuring 1.05 m in length and 0.18 m in diameter. The columns were filled with a homogenized mixture of soil, sand, and vermicompost in a 3:1:1 ratio, respectively. Plants in the well-watered treatment were maintained under normal environmental conditions, whereas drought-treated plants were placed in an area covered with a transparent sheet to simulate drought stress. Initially, three germinated seeds were planted per pot, with only one healthy seedling retained after 15 days. Before the onset of drought stress, columns were irrigated twice daily to maintain optimal soil moisture. Drought stress was initiated at the Z24 stage of Zadok’s scale (main shoot with four tillers), and root tissues were collected at the Z37 stage (flag leaf just visible). These root samples were immediately flash-frozen in liquid nitrogen and stored at −80°C for subsequent analyses (Zadoks and Board, 1999). The root systems were carefully extracted by breaking the pots and sectioned into four depths: 0–30 cm, 30–60 cm, 60–90 cm, and 90–120 cm, following the approach of Narayanan et al. (2014). Roots were gently washed using a low-pressure water fountain over a 1.5 m sieve to minimize damage. The cleaned root samples were then preserved in 70% ethanol and scanned using a document scanner. Root volume and other traits like root length and diameter were quantified using WinRHIZO^®^ software, which provides an accurate digital analysis of root morphological parameters (Singh et al., 2011).

### 2.2 RNA extraction, construction of small RNA libraries and deep sequencing

Total RNA was extracted using the TRIzol method, and its quality was assessed via NanoDrop ND-1000 spectrophotometer (NanoDrop Technologies, USA).Small RNAs were isolated in triplicate from frozen root tissues using the mirVana™ miRNA isolation kit (Ambion), following the manufacturer’s protocol. The small RNA fractions from the three replicates were pooled together for library construction. The preparation of small RNA libraries was performed according to the protocol by (Lu et al., 2007), with minor modifications. Small RNAs were sequentially ligated with 3’ and 5’ adapters (**Supplementary Table 1**). Following this, reverse transcription was performed using an RT primer, and PCR amplified the resulting cDNA. The integrity and quantity of the constructed libraries were assessed using the RNA Integrity Number (RIN) on an Agilent 2100 Bioanalyzer (Agilent Technologies, USA). The final libraries were submitted for high-throughput sequencing at SciGenome Labs (India) using the Illumina MiSeq platform (Illumina, USA). The libraries were labelled as Tolerant Control (TC), Tolerant Drought (TD), Susceptible Control (SC) and Susceptible Drought (SD). The raw data have been submitted to NCBI and the accession number is PRJNA1012115.

### 2.3 Computational analysis of small RNA sequencing data

Sequencing reads with a Phred quality score > 30 were retained and processed using Cutadapt v1.3 to remove adapter sequences. Non-coding small RNAs, including siRNAs, snRNAs, snoRNAs, piRNAs, tRNAs, and rRNAs, were filtered out by mapping them to respective databases (**Supplementary Table 2**) using Bowtie2 v2.1.0 (Langmead and Salzberg, 2012). The remaining non-redundant reads (17–35 nucleotides) were used for identifying both conserved and novel miRNAs in wheat. For conserved miRNAs, reads were aligned to the Triticum aestivum reference genome using Bowtie v1.2.3 (Langmead et al., 2009) and compared with known miRNAs in miRBase v22 (Kozomara et al., 2019). Initially, reads were mapped to mature miRNAs, followed by precursor sequences to ensure accuracy. Differential expression analysis was performed using DESeq2 in R v4.0.0 (Love et al., 2014) to identify significantly differentially expressed miRNAs.

For novel miRNA identification, miRDeep2 v2.0.0.7 (Friedländer et al., 2012) was used with the *Triticum aestivum* genome as a reference (Ensembl Plants Release 60: https://ftp.ebi.ac.uk/ensemblgenomes/pub/release-60/plants/fasta/triticum_aestivum/ncrna/). High-confidence novel miRNAs were identified based on (Meyers et al., 2008) criteria, including a 3’ two-nucleotide overhang, no more than four mismatches with the complementary precursor arm, and minimal asymmetric bulges (one or two bases) in the miRNA/miRNA* duplex. Secondary structure prediction was performed using MFOLD (Zuker, 2003) to confirm the characteristic stem-loop hairpin structure of miRNA precursors. Potential target genes for novel miRNAs were predicted using miRanda, which identifies mRNA targets based on sequence complementarity. Thisworkflow, integrating updated genome references and bioinformatics tools, ensures high-precision miRNA discovery and expression analysis, providing insights into their regulatory roles in gene silencing and stress response pathways in wheat.

### 2.4 Gene ontology analysis

**Figure 1:**
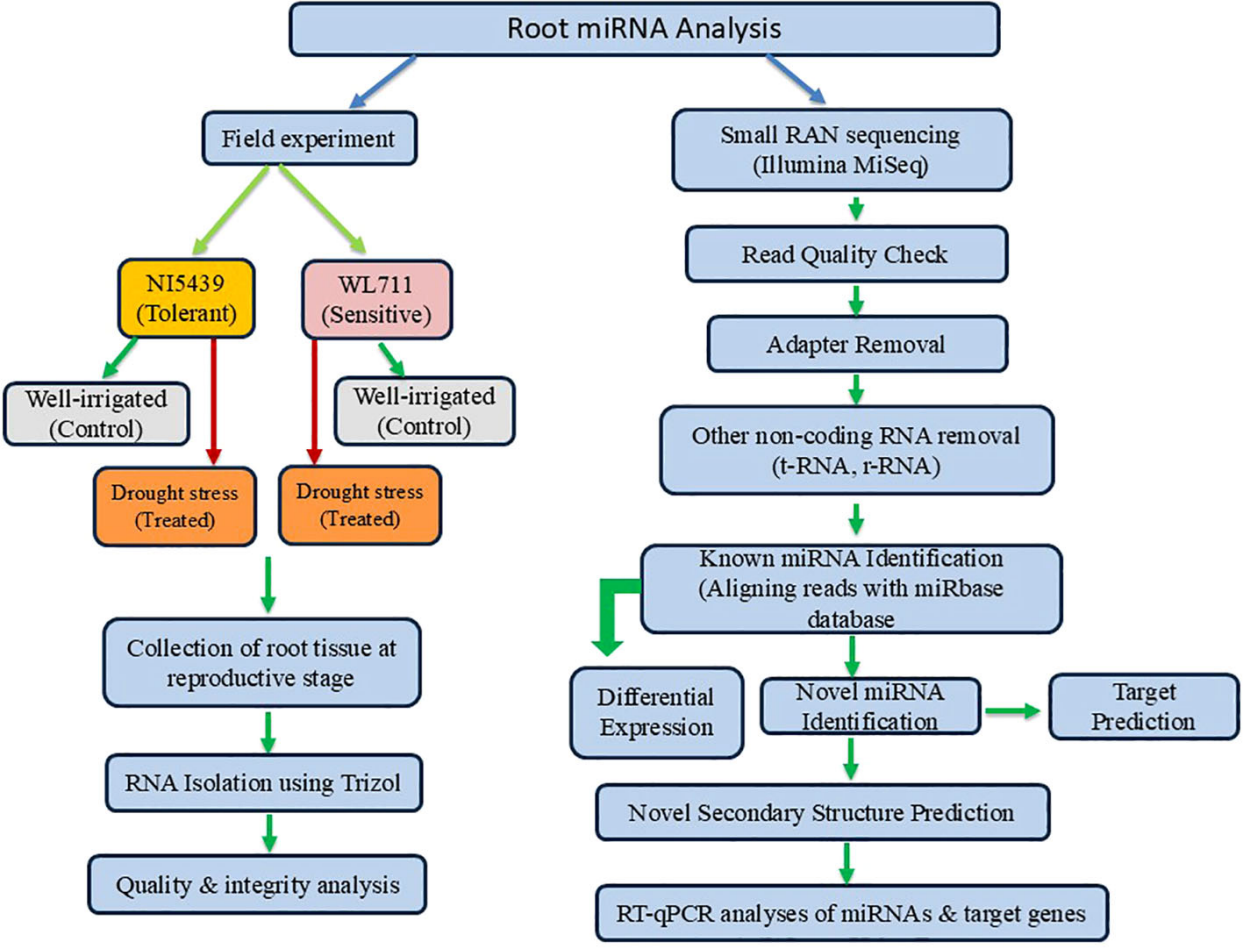
Summary of the approach employed for the identification, characterization, and functional validation of known and novel drought-responsive miRNAs in wheat root tissues at the reproductive stage.

To understand the functional roles of differentially expressed genes (DEGs) in wheat under drought stress, Gene Ontology (GO) enrichment analysis was performed. The DEGs were annotated using the BLAST2GO tool (Conesa et al., 2005) by mapping sequences against the non-redundant (NR) database of the National Center for Biotechnology Information (NCBI). The identified genes were classified into three major GO categories: biological process (BP), molecular function (MF), and cellular component (CC). The functional enrichment analysis was conducted using AgriGO v2.0, a specialized tool for plant GO analysis (Tian et al., 2017), to determine significantly overrepresented GO terms. The statistical significance of enrichment was assessed using Fisher’s exact test with a false discovery rate (FDR) < 0.05 for multiple testing corrections. **Figure 1** presents a summary of the miRNA analysis conducted under stress conditions, highlighting the key findings and insights from the study.

### 2.5 Gene regulatory network analysis

Cytoscape (version 3.2.1)tool was used for analysis of gene network analysis of differential expressed genes. For network analysis, top 100 upregulated and downregulated genes each were considered. ARACNE (Algorithm for the Reconstruction of Accurate Cellular Networks) and Network Analyzer plug-in were used for analyzing the network of all the four sets of DEGs. On the basis of high degree and betweenness, hub genes were selected.

### 2.6 Validation and expression profiling of miRNAs and their target genes by quantitative PCR

Small RNA cDNA (srcDNA) library was constructed according to the protocol of (Bailey et al., 2013). Briefly, small RNAs were polyadenylated at 37°C for 45 min in 50 μl reaction volume containing 0.3 μg ofmall RNA, 0.1 U *E. coli* poly(A) polymerase, 1X *E. coli* poly(A) polymerase reaction buffer [50 mM Tris-HCl, 250 mM NaCl, 10 mM MgCl_2,_ pH 7.9 at 25°C] and 1 mM ATP. Then, the poly(A)-tailed small RNA samples were purified to remove unincorporated ATP by using a purification cartridge provided in mirVana probe and marker kit as per manufacturer’s protocols. The purified poly(A)-tailed small RNA samples were stored at –70°C. The srcDNA libraries were generated by mixing 500 ng of poly(A)-tailed small RNA and 1 μg of RTQ primer in a 26 μl reaction volume. The reaction mixture was incubated at 65°C for 10 min followed by addition of 0.2 U M-MuLV reverse transcriptase, 1XM-MuLV reverse transcriptase reaction buffer [50 mM Tris-HCl, 75 mM KCl, 3 mM MgCl_2,_ 10 mM DTT, pH 8.3 at 25°C] and 1 mM dNTP mix in a final reaction volume of 40 μl. The reverse transcription was carried out at 37°C for 60 min followed by inactivation of the enzyme at 70°C for 15 min. About 1 μl of 5 U RNaseH was added to remove poly(A)-tailed small RNAs. The samples were purified by using the QIAquick PCR purification kit in 50 μl of final volume.

The qRT-PCR was performed using 0.3 μg of srcDNA, 1X SYBR green/fluorescein qPCR master mix, 1 μM RTQ-UNIr primer, and 1 μM miRNA-specific forward primer (**Supplementary Table 3**). The PCR reactions were performed in triplicate for each gene. The thermal cycling PCR reactions were performed with the following profile: 95°C for 5 min, 40 cycles of 15 sec denaturing at 94°C, 30-sec annealing at 55°C and 30-sec extension at 72°C, and finally a melt curve step from 65°C to 95°C with a rise of 0.5°C for 5 sec. The U6 snRNA was used as a reference gene for all the samples amplified. Relative quantification of expression for each miRNA was analyzed using the comparative CT method as described by (Livak and Schmittgen, 2001). Ten novel miRNAs viz. #ps_55, #ps _199, #ps _45 and #ps_160, #ps_19, #ps_91, #ps_187, #ps_103, #ps_74, #ps_47, #ps_89, #ps_157, #ps_55, #ps_121 and its targets TaDRA1, TaDRA2, TaDRA3, TaDRA4, TaDRA5, TaDRA6, TaDRA7, TaDRA8, TaDRA9, TaDRA10, TaDRA11, TaDRA12, TaDRA13 and TaDRA14 were chosen at random to validate them under drought stress in wheat respectively. The real ids of miRNAs and its targets were mentioned in (**Supplementary Table 4**).

## 3 Results

### 3.1 Phenotyping under drought stress

**Figure 2:**
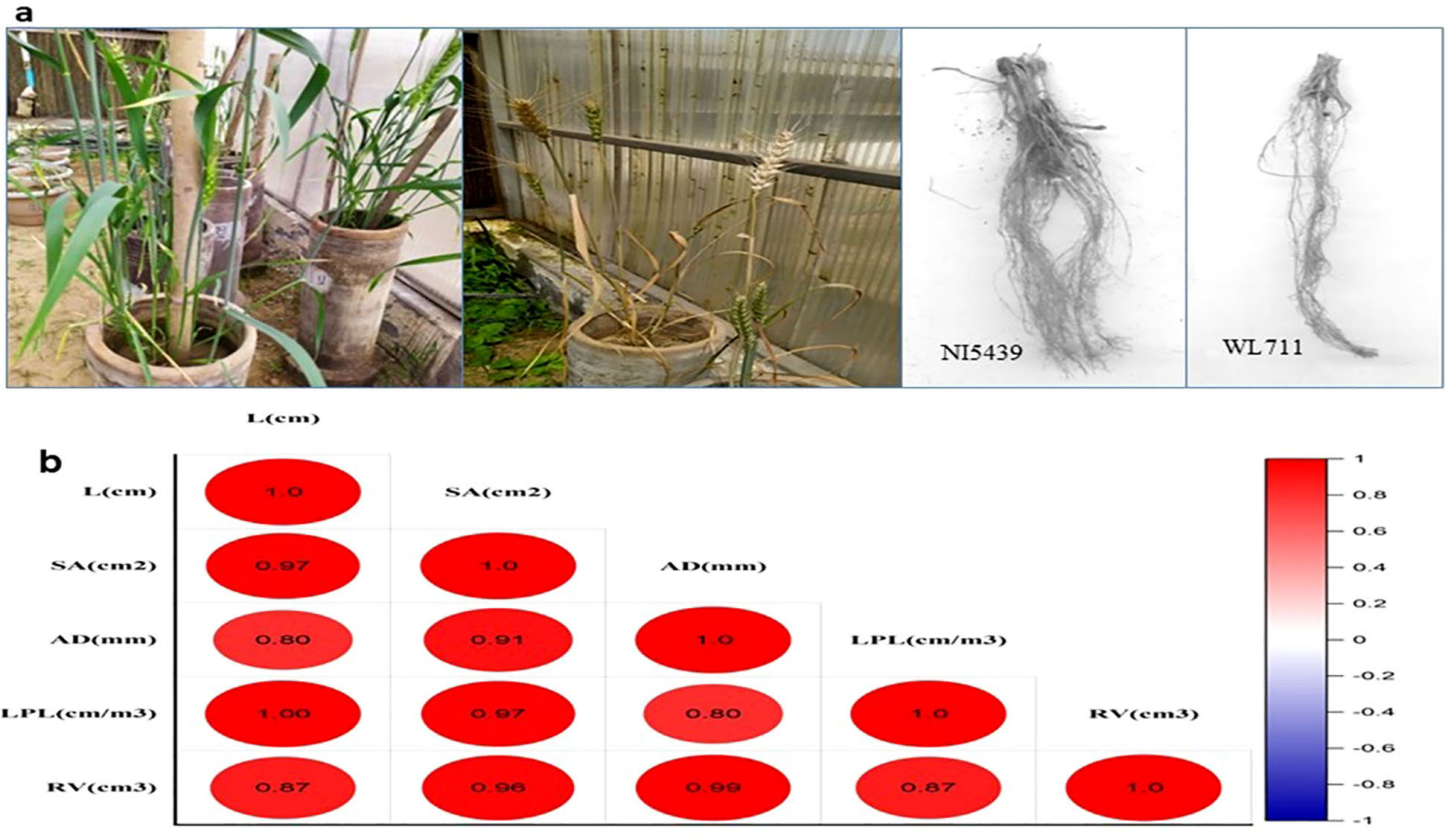
**(A)** Phenotyping of contrasting wheat genotypes under drought stress conditions to evaluate variations in root traits. **(B)** Correlation Matrix of Root Morphological Traits in Wheat under Drought Stress. L (cm) – Root Length, SA (cm²) – Root Surface Area, AD (mm) – Root Average Diameter, LPL (cm/m³) – Root Length Per Unit Volume, RV (cm³) – Root Volume. The figure represents a Pearson correlation matrix for root traits in wheat, with color intensity indicating correlation strength (red = positive, blue = negative).

Two wheat genotypes that were already known for their distinct behavior in drought stress were grown in fields under irrigated and drought conditions. Root samples were collected for small RNA library preparation at the booting stage of growth and development. The pooled analysis of variance revealed that both genotype and stress conditions differed significantly for all the traits studied. Based on LSD, the two genotypes differed in root length, surface area, and Length perVolume whereas stress conditions were significantly different for root length, surface area, and root volume.The two genotypes under normal conditions differed significantly for root length, surface area, and Length perVolume (**Figure 2**). However, under drought stress conditions, the two genotypes differed in total length and Length perVolume.Genotype 2 registered more reduction than genotype 1 in all the traits except diameter (**Figure 2**). Under normal conditions, the root length had a significant positive correlation with surface area and Length perVolume and the root volume had a significant positive correlation with surface area and average diameter. However, under drought-stressconditions, all the traits were interlinked positively.

### 3.2 Analysis of the small RNA libraries

Small RNA libraries were generated from the root tissues of two wheat genotypes, one exhibiting drought tolerance and the other drought sensitivity, cultivated under both normal and drought-stressed conditions. Sequencing produced approximately 200 million raw reads (200,780,257). Specifically, 62,320,791 reads were obtained from the drought-tolerant genotype under normal conditions, 73,006,712 from the same genotype under drought stress, 24,620,142 from the drought-sensitive genotype under normal conditions, and 40,832,612 from the drought-sensitive genotype under drought stress (**Table 1**). Raw sequencing reads underwent preprocessing to remove adapter sequences, yielding clean, non-redundant reads of 10,825,961, 6,203,540, 4,552,914, and 8,142,717 for the respective libraries. Non-coding RNAs, including siRNAs, snRNAs, snoRNAs, piRNAs, tRNAs, and rRNAs, were filtered out, leaving unannotated reads ranging from 17 to 35 nucleotides in length. These unannotated sequences were then analyzed to identify both conserved and novel miRNAs in wheat, offering valuable insights into miRNA-based regulatory mechanisms in response to drought stress.

**Table 1:** Summary of small RNA sequencing data in the four libraries ofwheat.

Category	TC (Tolerant-Control)			TD(Tolerant-Stressed)			SC (Sensitive-Control)			SD (Sensitive-Stressed)		
	Total Reads	Percent (%) Total Reads	Unique Reads	Total Reads	Percent (%) Total Reads	Unique Reads	Total Reads	Percent (%) Total Reads	Unique Reads	Total Reads	Percent (%) Total Reads	Unique Reads
Total raw reads	6,23,20,791	–	–	7,30,06,712	–	–	2,46,20,142	–	–	4,08,32,612	–	–
Clean reads	1,08,25,961	100	–	62,03,540	100	–	45,52,914	100	–	81,42,717	100	–
siRNA	1,33,821	1.24	627	1,13,169	1.82	627	97,455	2.14	627	1,43,269	1.76	627
piRNA	8,56,115	7.91	1,66,339	6,71,632	10.83	1,55,552	5,90,126	12.96	1,45,855	9,81,213	12.05	1,66,417
snRNA	7,696	0.07	289	6,926	0.11	287	4,687	0.10	286	6,700	0.08	292
snoRNA	15,133	0.14	1,060	13,249	0.21	1,049	9,954	0.22	1,032	15,547	0.19	1,058
tRNA	3,36,752	3.11	51,000	1,95,055	3.14	42,543	1,81,407	3.98	41,918	2,82,927	3.47	50,266
rRNA	40,97,899	37.85	9,31,773	21,57,834	34.78	6,30,847	17,59,917	38.65	5,80,263	26,39,542	32.42	7,92,532
Unannotated reads (4bp – 50bp)	53,78,545	49.68	–	30,45,675	49.10	–	19,09,368	41.94	–	40,73,519	50.03	–

### 3.3 Identification of conserved miRNAs

**Figure 3:**
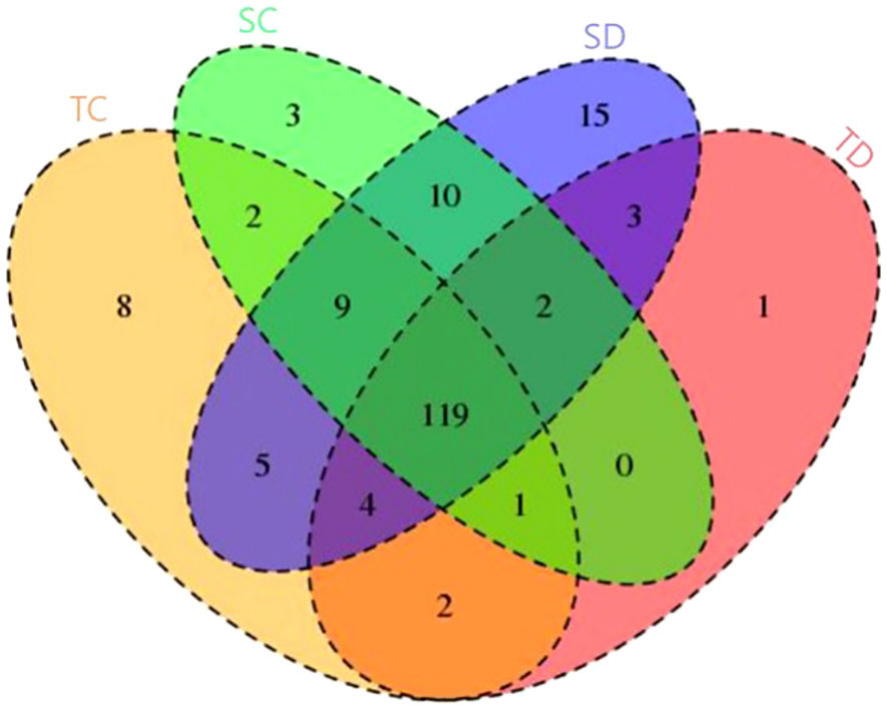
Venn diagram represents distribution of conserved miRNA among four libraries. illustrates the distribution of differentially expressed genes (DEGs) across four experimental conditions: TC (yellow), SC (green), SD (blue), and TD (red). Each section represents unique or shared DEGs among the conditions. The numbers indicate the count of DEGs specific to one condition or shared among multiple conditions.

The identification of conserved miRNAs was conducted by aligning 17–35 bp unique reads to a wheat reference genome. Initially, these reads were mapped to mature miRNAs, and any unmapped sequences were subsequently aligned with precursor sequences. This approach led to the identification of 150, 132, 146, and 167 previously characterized miRNAs, distributed across 86 families, in the tolerant-control (TC), tolerant-stressed (TD), sensitive-control (SC), and sensitive-stressed (SD) libraries, respectively (**Table 2**). Among these, 119 miRNAs were found to be shared across all libraries analyzed in this study (**Figure 3**). Additionally, several miRNAs were uniquely expressed under individual conditions, such as 8 in TC, 3 in SC, 15 in SD, and 1 in TD—suggesting condition-specific regulatory roles. Shared miRNAs between pairs and triplets of conditions also varied, with notable overlap between SC and SD (10 miRNAs) and between SC and TC (9 miRNAs). These results highlight both common and condition-specific miRNA-mediated regulatory responses under the tested stress conditions.

**Table 2:** Summary of conserved and novel miRNA in wheat under drought stress.

Sample Name	TC	TD	SC	SD
No. of mature miRNAs	59	52	60	72
No. of precursormiRNAs	91	80	86	95
No. of novel miRNAs	9	17	18	15

Notably, two miRNAs (tae-miR1119 and tae-miR9773) were exclusively detected under control conditions in both genotypes, whereas three miRNAs (tae-miR1129, tae-miR9660, and tae-miR9661) were specifically recovered under drought stress. These five miRNAs are likely to play a regulatory role in drought stress response. Additionally, 12 genotype-specific miRNAs were identified, with two (tae-miR1125 and tae-miR5049) associated with the drought-tolerant genotype, while the remaining ten (tae-miR1136, tae-miR9652, tae-miR9657b, tae-miR9657c, tae-miR9663, tae-miR9666a, and tae-miR9670) were linked to the drought-sensitive genotype.

**Figure 4:**
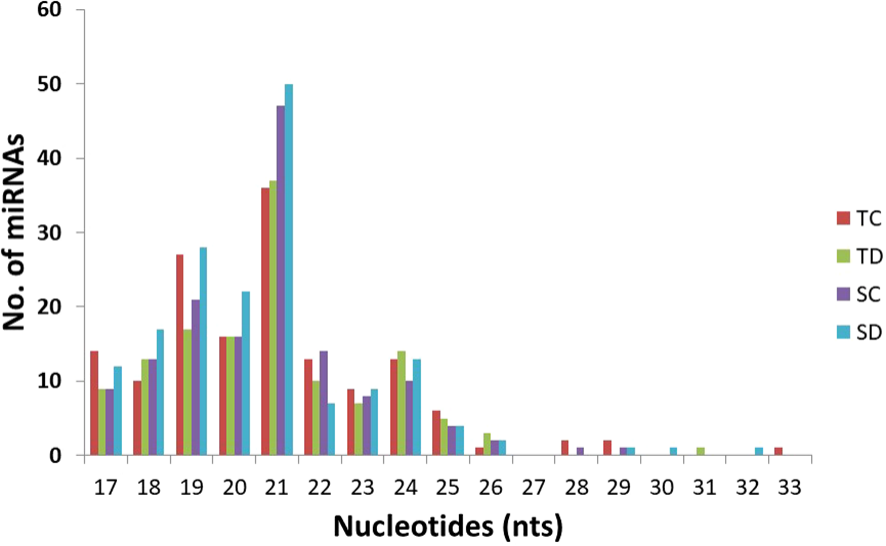
Length distribution of conserved miRNAs in the four libraries from wheat. TC: sRNA library from the drought-tolerant genotype NI-5439 without drought stress, TD: from NI-5439 with drought stress, SC: from the drought-sensitive genotype WL-711 without drought stress, SD: from WL-711 with drought stress.

The number of miRNA members varied across different families. The tae-miR159 and tae-miR9662 families exhibited the highest diversity, with an average of 20 members each. These were followed by tae-miR9653, tae-miR167, tae-miR1130, tae-miR9672, and tae-miR9657, each containing more than 10 members. The most highly conserved miRNA identified across all wheat libraries was tae-miR9662a-3p, along with its precursor tae-MIR9662a (**Supplementary Table 5**). The second and third most abundant miRNAs belonged to the conserved MIR159 family, specifically tae-miR159a and tae-miR159b. In terms of length distribution, the most prevalent class of conserved miRNAs was 21 nucleotides (28.57% on average), followed by the 19-nucleotide class (15.63% on average) (**Figure 4**).

### 3.4 Identification of Novel miRNAs

The discovery of novel miRNAs was achieved through secondary structure prediction, leading to the identification of 58 novel miRNAs in *Triticum aestivum*. These miRNAs were predicted from aligned sequencing data based on structural and genomic features. Among them, 9 novel miRNAs were detected in the control condition of the NI5439 genotype, while 10 were identified under drought stress. Similarly, in the WL711 genotype, 15 and 14 novel miRNAs were detected in control and drought-stressed samples, respectively (**Table 3**). Comprehensive characterization was conducted for each novel miRNA, including chromosomal localization, precursor sequence identification, secondary structure prediction, and mature miRNA sequence analysis. The secondary stem-loop structures were computationally predicted using an energy minimization approach. The minimal folding free energy (MFE) values of precursor miRNAs ranged from –46.71 to –13.47 kcal/mol, with an average of –33.73 ± 10.57 kcal/mol (**Table 3**), indicating their structural stability. The graphical representation of these structures is provided in **Supplementary Figure 1a-d**.

**Table 3:** Novel miRNAs identified by reference genome of *Triticum aestivum* in NI5439 and WL711 samples under control and drought stress condition.

miRNA ID	MFE	Chromosome	Sequence	Mature miRNA length	
(i) NI5439 control					
#ps111	-38.88	2A	uggacgaggaugugcagcugc	21	
#ps141	-38.88	2B	uggacgaggaugugcagcugc	21	
#ps25	-43.58	2D	uggacgaggaugugcagcugc	21	
#ps45	-18.89	3A	gcuugggcgagaguaguacuagg	23	
#ps55	-42.05	5B	ugaagcugccagcaugaucuga	22	
#ps59	-46.71	5B	ucggaccaggcuucauucccc	21	
#ps87	-41.69	5D	ugaagcugccagcaugaucuga	22	
#ps93	-46.58	5D	ucggaccaggcuucauucccc	21	
#ps56	-42.72	6D	cccgccuugcaccaagugaa	20	
(ii) NI5439 drought stress					
#ps187	-38.88	2A	uggacgaggaugugcagcugc	21	
#ps172	-13.47	2A	gaagacugcucugcuuugag	20	
#ps265	-38.88	2B	uggacgaggaugugcagcugc	21	
#ps47	-43.58	2D	uggacgaggaugugcagcugc	21	
#ps103	-46.71	5B	ucggaccaggcuucauucccc	21	
#ps157	-46.58	5D	ucggaccaggcuucauucccc	21	
#ps22	-22.31	6A	aaagacugcucugcuuugag	20	
#ps39	-22.31	6B	aaagacugcucugcuuugag	20	
#ps74	-42.72	6D	cccgccuugcaccaagugaa	20	
#ps191	-21.9	7B	ugauuguugcuugcguacacu	21	
(iii) WL711 control					
#ps137	-38.88	2A	uggacgaggaugugcagcugc	21	
#ps145	-30.74	2A	ccucgccggcugcgcguccacc	22	
#ps185	-38.88	2B	uggacgaggaugugcagcugc	21	
#ps19	-43.58	2D	uggacgaggaugugcagcugc	21	
#ps34	-21.3	3A	ugcugcguugacuggcgcuc	20	
#ps115	-32.46	3B	ccucgccggcugcgcguccacc	22	
#ps91	-46.71	5B	ucggaccaggcuucauucccc	21	
#ps94	-32.93	5B	ccucgccggcugcgcguccacc	22	
#ps87	-42.05	5B	ugaagcugccagcaugaucuga	22	
#ps112	-32.93	5D	ccucgccggcugcgcguccacc	22	
#ps125	-41.69	5D	ugaagcugccagcaugaucuga	22	
#ps133	-46.58	5D	ucggaccaggcuucauucccc	21	
#ps43	-15	6B	uauauuaucacucugaggga	20	
#ps161	-21.9	7B	ugauuguugcuugcguacacu	21	
#ps63	-42.72	6D	ucgcuuggugcagaucgggac	21	
(iv) WL711 drought stress					
#ps54	-34.27	1B	ugagaagguagaucauaauagc	22	
#ps55	-25.65	1B	uguuaugaucugcuucucauc	20	
#ps121	-38.88	2A	uggacgaggaugugcagcugc	21	
#ps157	-38.88	2B	uggacgaggaugugcagcugc	21	
#ps19	-43.58	2D	uggacgaggaugugcagcugc	21	
#ps89	-42.05	5B	ugaagcugccagcaugaucuga	21	
#ps91	-46.71	5B	ucggaccaggcuucauucccc	21	
#ps123	-41.69	5D	ugaagcugccagcaugaucuga	22	
#ps131	-46.58	5D	ucggaccaggcuucauucccc	21	
#ps20	-22.31	6A	aaagacugcucugcuuugag		
#ps25	-22.31	6B	aaagacugcucugcuuugag	20	
#ps55	-42.72	6D	ucgcuuggugcagaucgggac	21	
#ps168	-41.48	7D	ugcaucauuuggaacucgccg	20	
#ps173	-33.29	7D	uuccaaguugcguaguggaccgg	23	

Interestingly, two novel miRNAs, #ps19 and #ps91, were consistently detected in both libraries of the drought-sensitive genotype, suggesting their potential involvement in drought stress regulation. The most prevalent length among the novel miRNAs was 21 nucleotides, with a range spanning from 18 to 23 nucleotides. The GC content of these novel miRNAs averaged 55.67 ± 13.16%, indicative of their potential stability and functional relevance. Regarding chromosomal distribution, 13 loci were mapped to genome A, whereas genome B and genome D harbored 26 and 20 loci, respectively. Notably, the highest number of miRNA loci detected on a single chromosome was 8, found on chromosomes 5B and 5D, highlighting possible regions of miRNA enrichment in the wheat genome. These findings provide new insights into the regulatory landscape of wheat miRNAs, particularly under drought stress conditions.

### 3.5 Gene ontology

**Figure 5:**
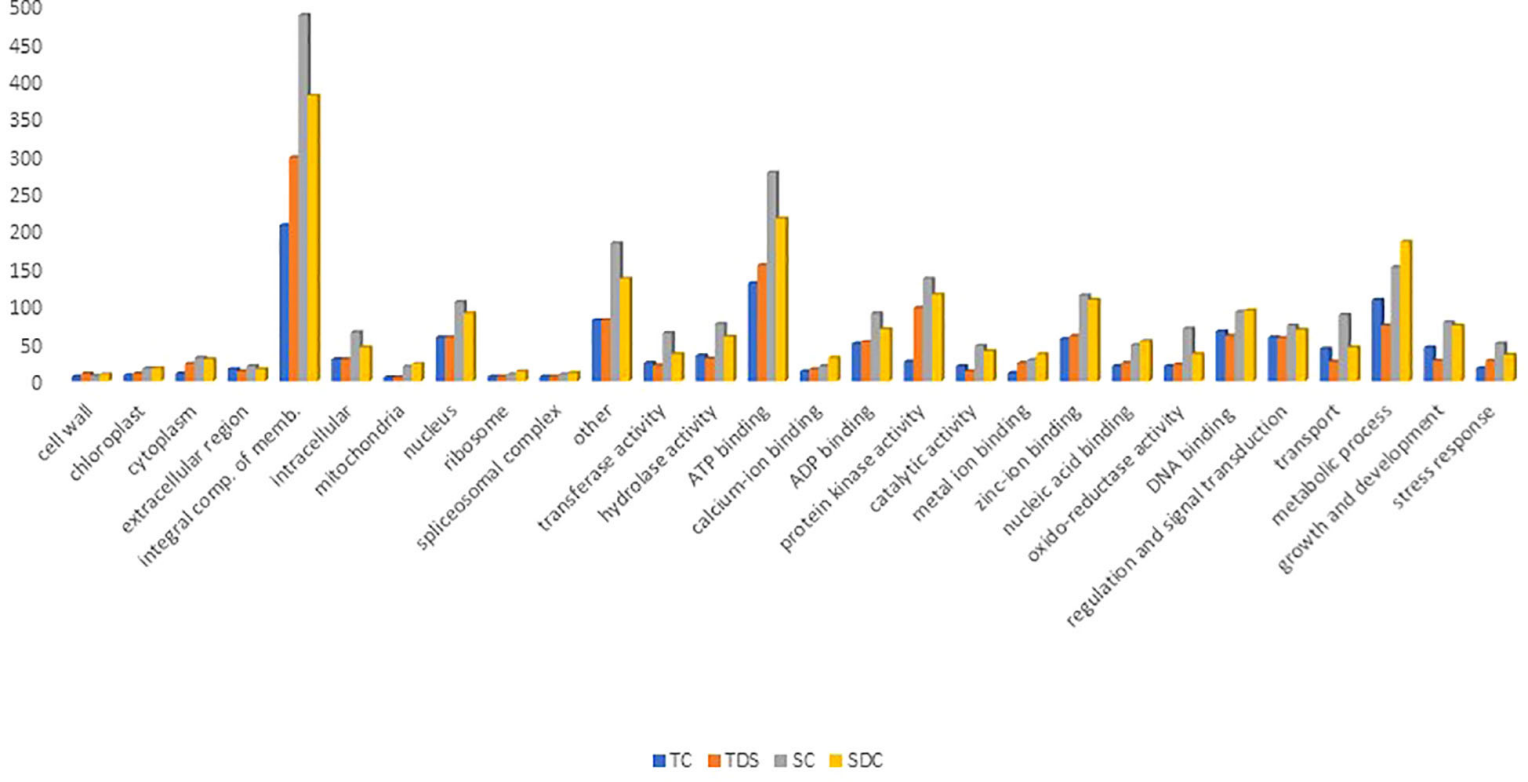
Gene ontology analysis of pooled libraries under control and stress conditions.

To annotate and analyze the functional roles of predicted miRNA target genes in wheat, a total of 4,551 target transcripts (740 in TC, 894 in TD, 1,340 in SC, and 1,577 in SD) were subjected to Gene Ontology (GO) analysis. These transcripts, associated with genes of known functions, were classified into biological processes, cellular components, and molecular functions based on their GO annotations (**Figure 5**).

In the molecular function category, the predicted miRNA targets were primarily linked to DNA, nucleic acid, and ion binding (183 terms in TC, 167 in TD, 302 in SC, and 322 in SD), catalytic activity (20 in TC, 13 in TD, 47 in SC, and 40 in SD), transferase activity (24 in TC, 21 in TD, 64 in SC, and 36 in SD), protein kinase activity (26 in TC, 97 in TD, 136 in SC, and 115 in SD), and oxidoreductase activity (20 in TC, 22 in TD, 70 in SC, and 36 in SD) (**Figure 5**; **Supplementary Table 6a-d**). In the biological processes category, many target transcripts were associated with stress response and defense mechanisms (17 terms in TC, 27 in TD, 50 in SC, and 35 in SD) and regulatory functions, including metabolic processes, growth and development, signal transduction, transcriptional regulation, and photosynthesis (**Figure 5**; **Supplementary Table 6a-d**). Similar patterns have been observed in rice and maize (Sunkar et al., 2012b; Božić et al., 2024). The cellular component category revealed that most target genes were associated with membrane components, while fewer were linked to organelles such as chloroplasts, mitochondria, ribosomes, and spliceosomes. The lower representation of chloroplast-related genes suggests a suppression of photosynthetic activity under stress, supported by the decline in photosynthesis-related GO terms in the biological process category. Interestingly, in resistant plants, plastid-associated terms increased under the cellular component category, although photosynthetic activity remained suppressed.

### 3.6 Gene regulating network of miRNAs

**Figure 6:**
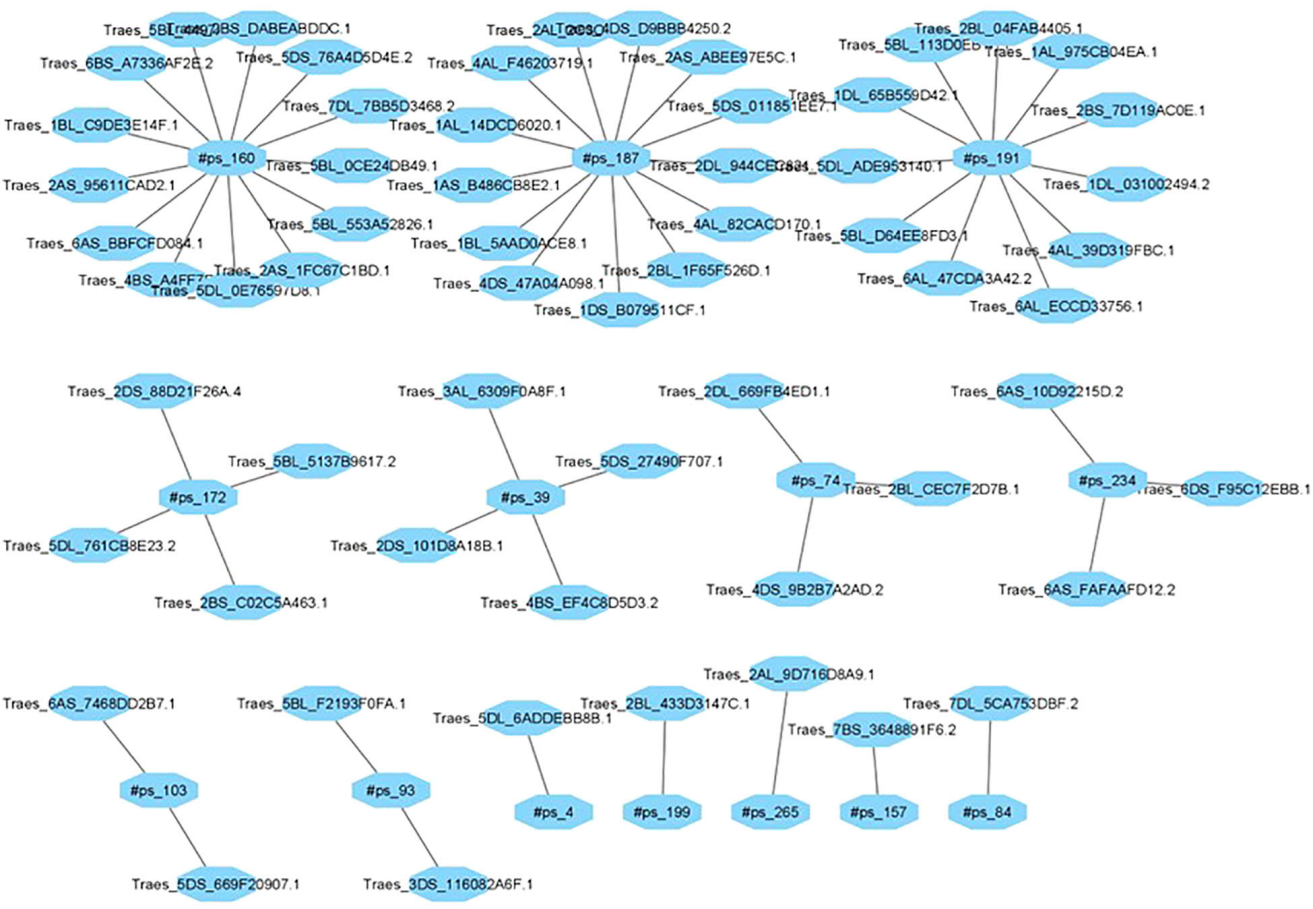
Network of miRNA targets in wheat genes under drought stress in the TD library.

The identification of miRNA-regulated target genes is essential for elucidating the functionalroles of miRNAs in plants, particularly in response to environmental stress. Computational target prediction revealed 314 putative target genes for differentially expressed miRNAs in wheat, spanning various biological and molecular functions. In the tolerant-control (TC) library, five miRNAs were predicted to regulate 41 genes, while 13 novel miRNAs were associated with the regulation of 60 genes in the tolerant-drought (TD) library (**Figure 6**). Similarly, in the sensitive-control (SC) library, 11 miRNAs were linked to 78 target genes, whereas in the sensitive-drought (SD) library, eight novel miRNAs were predicted to influence the expression of 134 genes under both control and drought stress conditions (**Supplementary Figure 2a-c**).

The predicted target genes exhibit a broad spectrum of functional categories, underscoring their pivotal roles in plant growth, development, and stress adaptation. Notably, several identified miRNAs target key transcription factor (TF) families, including MYB, NAC, WRKY, and bZIP, which serve as master regulators of gene expression networks balancing abiotic stress responses, hormone signaling, and developmental processes. The enrichment of TFs among miRNA targets suggests a hierarchical regulatory mechanism wherein miRNAs modulate multiple downstream genes, thereby influencing extensive biological pathways. Additionally, a subset of target genes encodes proteins associated with phytohormonal signaling, such as auxin response factors (ARFs) and abscisic acid (ABA)-responsive elements, which are integral to stress tolerance, growth regulation, and developmental plasticity under drought stress. The identification of histone-modifying enzymes, including histone deacetylases (HDACs) and methyltransferases, implies a potential role for miRNAs in chromatin remodeling and epigenetic regulation, thereby contributing to transcriptional stability and adaptive gene expression in response to environmental stressors. Furthermore, several miRNAs were predicted to regulate genes involved in ion transport and homeostasis, such as potassium and calcium transporters, which are essential for maintaining osmotic balance and intracellular signaling under drought conditions. The identification of miRNA-targeted proteases suggests a role in stress-induced proteolysis, which may facilitate cellular adaptation by regulating protein turnover under adverse conditions. Additionally, the modulation of kinase-mediated phosphorylation cascades by miRNAs indicates their involvement in signal transduction pathways, influencing cellular responses to external stimuli. Collectively, these findings highlight the intricate miRNA-mediated regulatory networks that fine-tune gene expression, thereby enhancing wheat’s adaptive potential to drought stress. A deeper understanding of these interactions could provide a foundation for developing stress-resilient wheat cultivars through targeted genetic and biotechnological approaches.

### 3.7 *In silico* expression analysis of miRNAs

**Figure 7:**
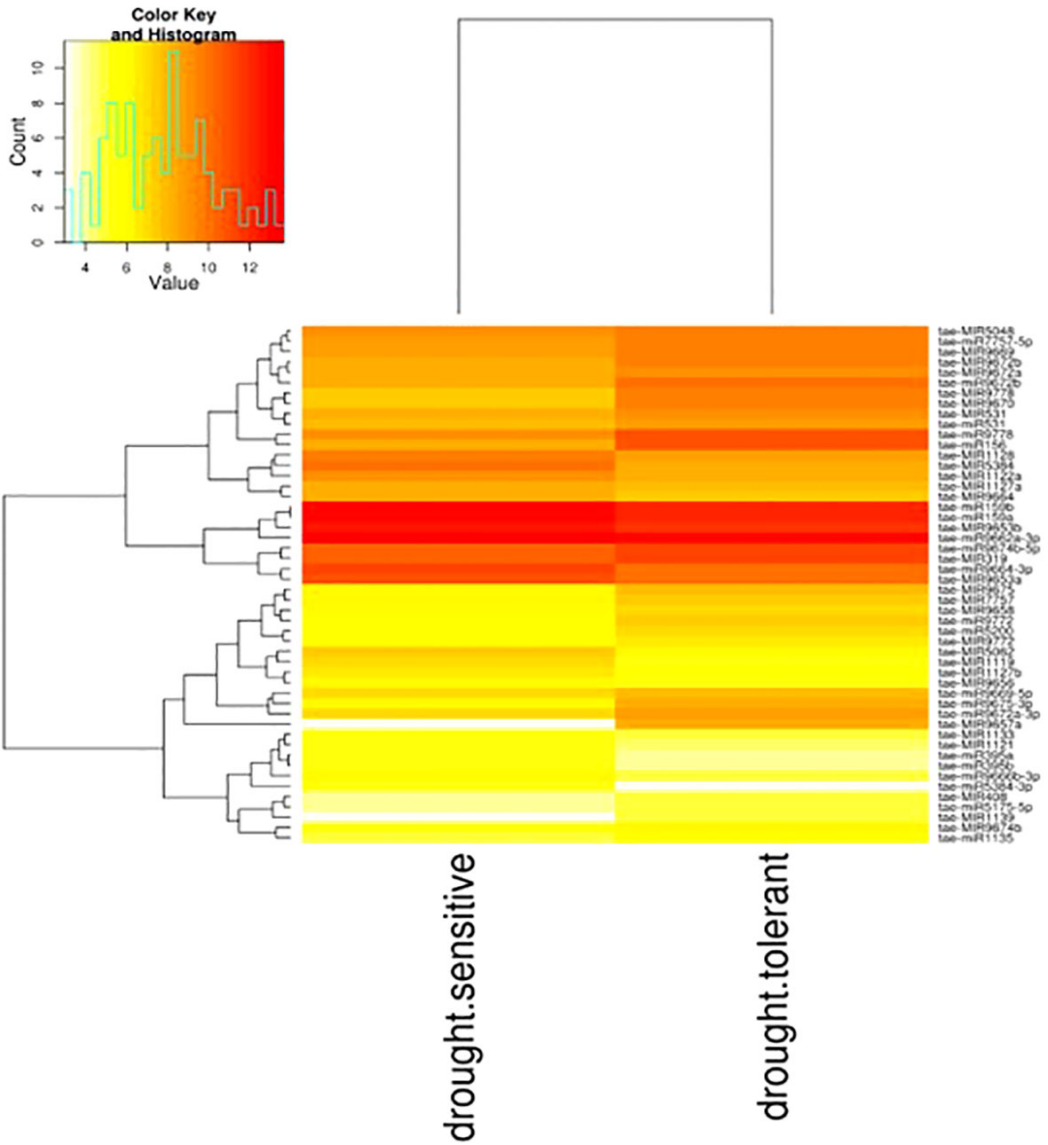
Visualizing differential expression between sample drought-tolerant and drought-sensitive using g-plot heatmap from Wheat. The plot shows the hierarchical clustering of sample both samples. (Yellow shows low-level expression; Red shows high-level expression).

The differential expression patterns of conserved miRNAs in both wheat genotypes were analyzed to assess their regulatory roles under drought stress conditions. Hierarchical clustering of all samples was performed to visualize expression trends, with the heatmap representing distinct expression profiles across different experimental conditions (**Figure 7**).

A total of 18 mature miRNAs exhibited significant differential expression based on fold change criteria (<1 or >1), indicating their potential involvement in drought stress response. Among them, six miRNAs—*tae-miR395a, tae-miR395b, tae-miR5049-3p, tae-miR5384-3p, tae-miR9664-3p*, and *tae-miR9666b-3p*—were significantly upregulated during drought stress, suggesting their potential role in activating stress-responsive pathways (**Table 4**). These miRNAs may be involved in regulating sulfur metabolism, oxidative stress responses, and transcriptional regulation under water-limiting conditions.

**Table 4:** Comparative expression Profile of miRNAs in contrasting wheat genotypes.

S.No.	miRNA	log2	pval	padj
1.	tae-miR156	-3.15	0.02	0.63
2.	tae-miR9778	-2.82	0.03	0.64
3.	tae-miR9675-3p	-2.67	0.05	0.77
4.	tae-miR9672a-3p	-2.54	0.05	0.77
5.	tae-miR9672b	-2.29	0.07	0.82
6.	tae-miR9772	-2.07	0.07	0.82
7.	tae-miR531	-1.48	0.24	1.00
8.	tae-miR5200	-1.44	0.15	1.00
9.	tae-miR9669-5p	-1.25	0.34	1.00
10.	tae-miR5175-5p	-1.25	0.21	1.00
11.	tae-miR7757-5p	-1.04	0.40	1.00
12.	tae-miR1135	-1.04	0.28	1.00
13.	tae-miR9666b-3p	1.06	0.28	1.00
14.	tae-miR395b	1.18	0.24	1.00
15.	tae-miR5049-3p	1.18	0.51	1.00
16.	tae-miR395a	1.28	0.20	1.00
17.	tae-miR9664-3p	1.65	0.15	1.00
18.	tae-miR5384-3p	2.51	0.02	0.63

Conversely, two-thirds of the differentially expressed miRNAs were downregulated under drought stress, including *tae-miR156, tae-miR1135, tae-miR531, tae-miR5175-5p, tae-miR5200, tae-miR7757-5p, tae-miR9669-5p, tae-miR9672a-3p, tae-miR9672b, tae-miR9675-3p, tae-miR9772*, and *tae-miR9778*. The repression of these miRNAs may be linked to the modulation of stress-adaptive processes such as leaf morphogenesis, hormone signaling, and secondary metabolite biosynthesis, which are critical for drought tolerance.These results suggest that specific miRNAs play contrasting roles in drought stress adaptation by either enhancing or suppressing gene expression networks associated with plant survival under water-deficient conditions. The observed differential expression patterns provide valuable insights into the complex regulatory mechanisms governing stress responses in wheat, offering potential targets for improving drought resilience through genetic or biotechnological approaches.

### 3.8 Validation of novel miRNAs

High-throughput deep sequencing of root tissues from contrasting wheat genotypes revealed several putative novel miRNAs involved in drought stress response. To validate these findings, plants were cultivated under both control and drought conditions, followed by qPCR-based expression analysis in root tissues. A subset of ten novel miRNAs and their corresponding target genes were randomly selected for validation, including *#ps_55, #ps_199, #ps_45, #ps_160, #ps_19, #ps_91, #ps_187, #ps_103, #ps_74, #ps_47, #ps_89, #ps_157, #ps_55*, and *#ps_121*. Their predicted target genes (*TaDRA1–TaDRA14*) encode proteins involved in drought response and adaptation mechanisms, including stress-responsive transcription factors, osmotic regulation proteins, and key enzymes in antioxidant defense pathways.

**Figure 8:**
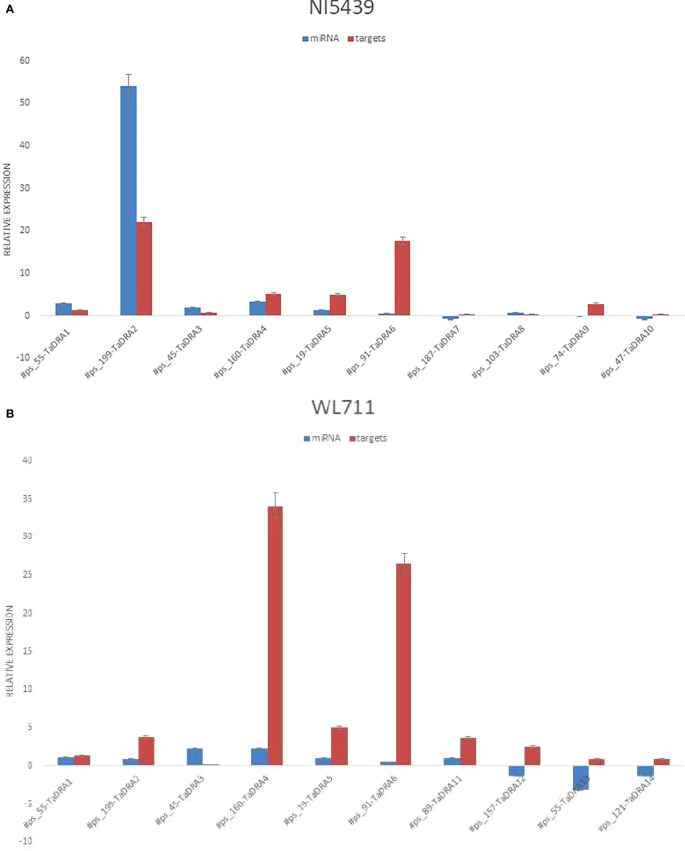
**(A)** Relative expression levels of miRNAs and their corresponding targets in root tissue of the drought-tolerant genotype NI5439. The orange colour represents the miRNA and blue represents the wheat genes. **(B)** Relative expression levels of miRNAs and their corresponding targets in the drought-susceptible genotype WL711 root tissue.

Expression profiling in root tissues revealed genotype-specific regulatory patterns of the novel miRNAs, with distinct expression dynamics between the drought-tolerant (NI5439) and drought-sensitive (WL711) genotypes (**Figures 8A, B**). Notably, *#ps_199* exhibited significantly higher expression in the root tissues of the NI5439 genotype compared to other miRNAs, suggesting a potential role in root-specific drought response mechanisms. The NI5439 genotype displayed elevated expression levels of novel miRNAs relative to WL711, indicating a more robust miRNA-mediated regulatory network in drought adaptation.The identified miRNAs and their associated target genes likely participate in key physiological and molecular pathways, including hormone signaling, osmoprotectant biosynthesis, oxidative stress mitigation, and root system architecture modulation.

## 4 Discussion

Drought stress is one of the most significant abiotic factors limiting wheat productivity (*Triticum aestivum* L.). To cope with water-deficit conditions, plants have evolved complex regulatory mechanisms, including miRNA-mediated gene regulation, which plays a crucial role in modulating stress responses at the post-transcriptional level. This study provides a comprehensive analysis of drought-responsive miRNAs in wheat, identifying both conserved and novel miRNAs, along with their target genes and functional pathways. The findings were validated through expression profiling, functional annotation, and comparative analysis with prior studies, offering insights into miRNA-mediated drought tolerance mechanisms.

The identification of conserved and novel miRNAs is crucial for understanding the regulatory landscape of drought stress responses. In this study, multiple conserved miRNAs, including tae-miR159, tae-miR395, tae-miR156, tae-miR398, and tae-miR319, were differentially expressed in response to drought stress. These findings align with prior research in wheat and other cereals, where these miRNAs have been implicated in drought tolerance mechanisms (Sunkar et al., 2007; Akdogan et al., 2016; Zhao et al., 2023a). The upregulation of tae-miR395 in this study is consistent with previous reports highlighting its role in sulfur metabolism and stress adaptation (Zhou et al., 2010; Wang et al., 2013). Similarly, tae-miR398, known to regulate Cu/Zn superoxide dismutase (CSD) genes involved in ROS detoxification, was significantly upregulated, reinforcing its importance in oxidative stress mitigation (Gupta et al., 2014). In addition to conserved miRNAs, 59 novel miRNAs were identified, with several exhibiting genotype-specific expression patterns. Notably, #ps_91 was highly expressed in the drought-tolerant genotype, suggesting their potential role in stress resilience. Similar genotype-dependent miRNA expression has been reported in wheat, barley, and soybean, supporting the hypothesis that novel miRNAs contribute to adaptive stress responses (Gómez-Martín et al., 2023; Božić et al., 2024).

Hierarchical clustering and differential expression analysis revealed that several miRNAs exhibited significant upregulation or downregulation in response to drought stress. A total of 18 mature miRNAs were significantly expressed based on fold-change criteria. Among them, six miRNAs (tae-miR395a, tae-miR395b, tae-miR5049-3p, tae-miR5384-3p, tae-miR9664-3p, and tae-miR9666b-3p) were upregulated, whereas 12 miRNAs, including tae-miR156, tae-miR1135, tae-miR531, and tae-miR5200, were downregulated under drought conditions.The upregulation of tae-miR395 has been linked to enhanced sulfur metabolism and secondary metabolite biosynthesis, essential for stress adaptation (Kawakshima et al., 2009; Zhang J et al., 2022). The downregulation of tae-miR156 is of particular interest, as it has been previously associated with enhanced shoot growth and delayed flowering under stress conditions (Xu et al., 2021). These findings are consistent with prior studies in Arabidopsis and Oryza sativa, where miR156 negatively regulates SPL transcription factors, thereby influencing drought responses (Wang et al., 2011; Trindade et al., 2010). In contrast to previous studies where miR319 was downregulated under drought stress in wheat (Akdogan et al., 2016), this study observed its significant upregulation, particularly in tolerant genotypes. Given its role in targeting TCP transcription factors, this suggests that miR319 might be involved in modifying leaf morphology and cell wall biosynthesis to counteract stress effects.

The identified miRNA-target interactions highlight a complex regulatory network governing stress adaptation in wheat, aligning with previous findings in other plant species. The results demonstrated that several miRNAs target transcription factor (TF) families, including MYB, NAC, WRKY, and bZIP, which are critical regulators of gene expression under abiotic stress. Similar miRNA-mediated regulation of TFs has been reported in Arabidopsis and rice, where these TF families modulate drought-responsive pathways by controlling stress-inducible gene expression (Zhao et al., 2019; Li et al., 2021). The presence of hormone-related target genes, such as auxin response factors (ARFs) and abscisic acid (ABA)-responsive elements, further supports the role of miRNAs in integrating hormonal signaling with stress responses. Studies in maize and barley have demonstrated that miRNAs modulate ABA signaling to fine-tune stomatal regulation and osmotic balance under water-deficit conditions (Guan et al., 2020).

**Figure 9:**
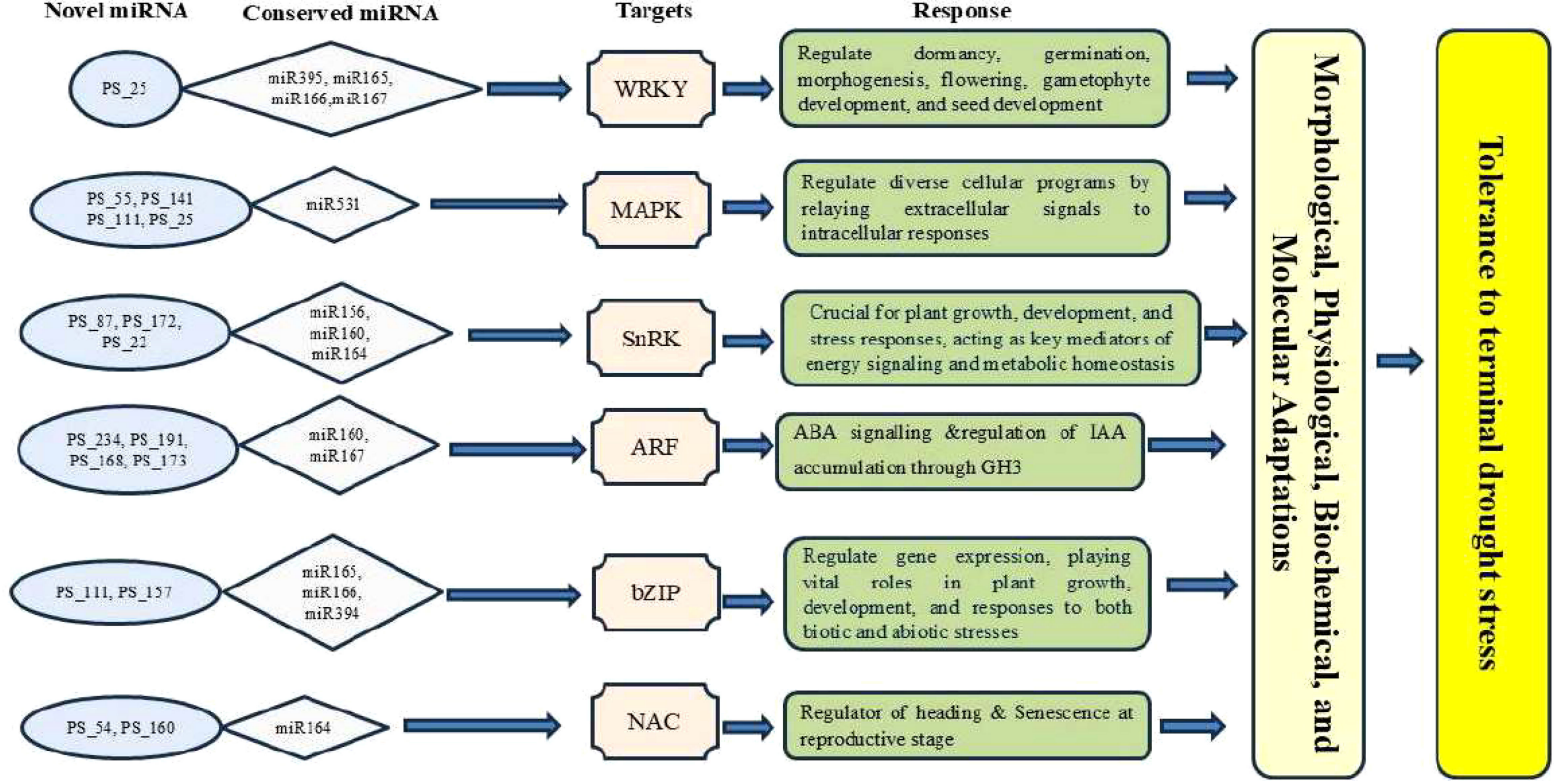
Illustrated depiction of the miRNA-mediated regulatory gene network involving known and novel miRNAs under drought stress at the reproductive stage in wheat.

Gene ontology (GO) and KEGG pathway analyses revealed that the miRNA target genes are involved in critical biological processes, including transcriptional regulation, hormone signaling, ion transport, osmotic balance, and ROS detoxification.Several miRNAs were found to target transcription factors, including members of the WRKY, NAC, MYB, and bZIP families. These transcription factors are well-documented regulators of abiotic stress responses (He et al., 2016; Zhang Y et al., 2022). The downregulation of tae-miR169, which targets NF-YA transcription factors, suggests its role in modulating drought-responsive genes, consistent with previous findings in wheat and rice (Zhao et al., 2007; Bakhshi et al., 2014). The involvement of miRNAs in hormone signaling pathways was also evident. tae-miR159 was found to regulate MYB transcription factors involved in ABA signaling, highlighting its role in stress-adaptive hormone responses (Reyes and Chua, 2007; Pandey et al., 2013). Similarly, tae-miR160, which targets auxin response factors (ARFs), exhibited significant expression changes, suggesting its role in modulating root development and drought adaptation (Ding et al., 2013). One of the most striking findings was the regulation of ROS scavenging and oxidative stress pathways. The upregulation of tae-miR398, which regulates superoxide dismutase (SOD), aligns with previous studies demonstrating miRNA-mediated control of oxidative stress responses in wheat and rice (Zhan and Meyers, 2023; Zhou et al., 2024). This supports the hypothesis that miRNA-regulated ROS detoxification plays a central role in drought tolerance.The identification of drought-responsive miRNAs provides a foundation for developing stress-resilient wheat varieties through molecular breeding and genetic engineering. The differential expression of miRNAs between drought-tolerant and drought-sensitive genotypes suggests that miRNAs could serve as biomarkers for selecting drought-adaptive traits in breeding programs.Several miRNAs identified in this study, particularly those regulating transcription factors, hormone signaling genes, and antioxidant enzymes, could be targeted for CRISPR/Cas9-based genome editing to enhance stress tolerance (Jaganathan et al., 2018; Tang and Chu, 2023). Based on our findings, we present a model illustrating miRNA-mediated gene regulation and stress responses in wheat under reproductive stage drought stress (**Figure 9**). Future studies should focus on functional validation of key miRNAs using transgenic approaches and miRNA knockout strategies to elucidate their precise roles in drought adaptation.Furthermore, integrating miRNA expression data with proteomics, metabolomics, and physiological measurements could provide a more comprehensive understanding of drought stress regulatory mechanisms in wheat. Multi-omics approaches will be crucial in unravelling the complex interplay between miRNAs and their targets, ultimately aiding in the development of climate-resilient crops (Zhao et al., 2023b).

## 5 Conclusion

In this study, four sRNA libraries were constructed from roots of drought-tolerant NI-5439 and drought-sensitive WL-711 wheat genotype from control and drought stress conditions at the booting stage. In total, 306 conserved and 58 novel microRNAs were identified from the four libraries. After computational expression analysis of mature miRNAs, 18 miRNAs showed significant changes in the expression after stress treatment. For the first time, 15 conserved miRNAs were emerged as drought-responsive in this study. The predicted targets of novel miRNAs were genes involved in gene silencing by RNA, DNA methylation, histone modification and chromatin modification. This study has significantly expanded the number of novel as well as drought-responsive miRNAs in wheat.

## Data Availability

The data have presented in the study are deposited in the NCBI, accession number is PRJNA1012115.
