# Supplementary material for: Novel and conserved drought-responsive microRNAs expression analysis in root tissues of wheat (Triticum asetivum L.) at reproductive stage
Source: Front Plant Sci. 2025 May 20;16:1581542. doi: 10.3389/fpls.2025.1581542 (PMC12129965; doi:10.3389/fpls.2025.1581542)
Supplement: Supplementary file 1 [file Table1.docx]

Supp. Table 1. List of adapters and primers used for small RNA library preparation.

| **S. No.** | **Adapter/Primer** | **Sequence (5' to 3')** | **Description** |
| --- | --- | --- | --- |
|  | 5' RNA adapter | GUU CAG AGU UCU ACA GUC CGA CGA UC | RNA oligonucleotide |
|  | 3' RNA adapter | pUUU CGU AUG CCG UCU UCU GCU UGU idT* | RNA oligonucleotide |
|  | RT Primer | CAA GCA GAA GAC GGC ATA CGA | DNA oligonucleotide |
|  | sRNA Primer 1 | ATA GGA TCC CAA GCA GAA GAC G | DNA oligonucleotide |
|  | sRNA Primer 2 | CGA GTC GAC GTT CAG AGT TCT A | DNA oligonucleotide |
|  | sRNA Seq Primer | AGT TCT ACA GTC CGA CGA TC | DNA oligonucleotide |

* p, phosphate; idT, inverted deoxythymidine

Supp. Table 2: List of databases used to annotate non-coding small RNAs.

| **S. No.** | **Small RNA*** | **Database** | **URL** | **Reference** |
| --- | --- | --- | --- | --- |
|  | tRNA | GtRNAdb | http://gtrnadb.ucsc.edu/ | Lowe and Eddy 1997 |
|  | rRNA | Rfam | http://rfam.xfam.org/ | Nawrocki et al. 2015 |
|  | piRNA | piRNABank | http://pirnabank.ibab.ac.in/index.shtml | Sai-Lakshmi and Agrawal 2008 |
|  | siRNA | siRNAdb | http://siRNA.cgb.ki.se | Chalk et al. 2005 |
|  | snRNA | NCBI Genbank | http://www.ncbi.nlm.nih.gov/genbank/ | Benson et al. 2013 |
|  | snoRNA | deepBase | http://rna.sysu.edu.cn/deepBase/download.php | Zheng et al. 2016 |

* RNA, ribonucleic acid; tRNA, transfer RNA; rRNA, ribosomal RNA; piRNA, piwi-interacting RNA; siRNA, small interfering RNA; snRNA, small nuclear RNA; snoRNA, small nucleolar RNA; NCBI, National Center for Biotechnology Information

Supp. Table 3: Actual and assigned IDs of miRNAs and their targets used for validation.

| Noval miRNA ids | Assign ids | Targets assign ids | Targets ids |
| --- | --- | --- | --- |
| >>chr5B_55 | #ps_55 | TaDRA1 | Traes_4BS_EF4C8D5D3.2 |
| >>chr7B_199 | #ps_199 | TaDRA2 | Traes_3DS_116082A6F.1 |
| >>chr3A_45 | #ps_45 | TaDRA3 | Traes_2AS_6AB3D73F7.1 |
| >>chr7A_160 | #ps_160 | TaDRA4 | Traes_5AL_E4E5B111A.1 |
| >>chr2D_19 | #ps_19 | TaDRA5 | Traes_1BL_D9AD1E5B1.2 |
| >>chr5B_91 | #ps_91 | TaDRA6 | Traes_6AS_7468DD2B7.1 |
| >>chr2A_187 | #ps_187 | TaDRA7 | Traes_1AL_14DCD6020.1 |
| >>chr5B_103 | #ps_103 | TaDRA8 | Traes_7DL_806E4512D.1 |
| >>chr6D_74 | #ps_74 | TaDRA9 | Traes_6AS_8119B6846.1 |
| >>chr2D_47 | #ps_47 | TaDRA10 | Traes_1AL_14DCD6020.1 |
| >>chr5B_89 | #ps_89 | TaDRA11 | Traes_7BL_44A22C7FC.2 |
| >>chr2B_157 | #ps_157 | TaDRA12 | Traes_2DS_97E3E7CFC.1 |
| >>chr1B_55 | #ps_55 | TaDRA13 | Traes_4AL_39D319FBC.1 |
| >>chr2A_121 | #ps_121 | TaDRA14 | Traes_1AL_14DCD6020.1 |

Supp. Table 4: List of primers used for validation of miRNAs.

| **S. No.** | **Primer** | **Primer sequence (5' to 3')** |
| --- | --- | --- |
|  | chr5B_55 | ATG ACC TAA CTC ATG GAT CAG AG |
|  | chr7B_199 | CGG AAA GTC CTT GGG CTG T |
|  | chr3A_45 | GCT TGG GCG AGA GTA GTA CTA GG |
|  | chr7A_160 | GAG AGA AGG TCG CCT GAC |
|  | chr2A_187 | TGGACGAGGATGTGCAGCTGC |
|  | chr5B_103 | TCGGACCAGGCTTCATTCCCC |
|  | chr6D_74 | CCCGCCTTGCACCAAGTGAA |
|  | chr2D_47 | TGGACGAGGATGTGCAGCTGC |
|  | chr5B_89 | TGAAGCTGCCAGCATGATCTGA |
|  | chr2B_157 | TGGACGAGGATGTGCAGCTGC |
|  | chr1B_55 | TGTTATGATCTGCTTCTCATC |
|  | chr2A_121 | TGGACGAGGATGTGCAGCTGC |
|  | U6 snRNA | AAC AGT CTG ACT TGT CCC TTC |
|  | RTQ-UNIr | CGA ATT CTA GAG CTC GAG GCA GG |
|  | RTQ Primer | CGA ATT CTA GAG CTC GAG GCA GGC GAC ATG GCT GGC TAG TTA AGC TTG GTA CCG AGC TCG GAT CCA CTA GTC C(T)_25_ |

* UNIr, universal reverse primer

Supp Table 5. Frequency of conserved and non-conserved miRNA families in wheat.

| **miRNA Family** | **Frequency** | | | |
| --- | --- | --- | --- | --- |
|  | **Tolerant-Control** | **Tolerant-Drought** | **Sensitive-Control** | **Sensitive-Drought** |
| MIR156 | 143 | 860 | 148 | 3183 |
| MIR159 | 10315 | 33306 | 8559 | 11766 |
| MIR160 | 230 | 430 | 192 | 254 |
| MIR164 | 218 | 358 | 270 | 248 |
| MIR167 | 935 | 1628 | 639 | 2356 |
| MIR169 | 15 | - | 5 | 1 |
| MIR171 | 45 | 26 | 30 | 36 |
| MIR319 | 893 | 1896 | 922 | 3529 |
| MIR395 | 50 | 20 | 10 | 8 |
| MIR396 | 54 | 86 | 60 | 45 |
| MIR397 | 137 | 88 | 101 | 70 |
| MIR398 | 1642 | 1610 | 2028 | 472 |
| MIR399 | 2 | - | - | 1 |
| MIR408 | 265 | 770 | 277 | 185 |
| MIR444 | 360 | 420 | 272 | 391 |
| MIR530 | 1 | 2 | - | 1 |
| MIR531 | 202 | 334 | 501 | 582 |
| MIR1117 | 7 | 12 | 6 | 8 |
| MIR1118 | 27 | 20 | 13 | 40 |
| MIR1119 | 67 | 26 | 20 | 4 |
| MIR1120 | 200 | 236 | 129 | 117 |
| MIR1121 | 12 | 32 | 5 | 8 |
| MIR1122 | 432 | 460 | 154 | 229 |
| MIR1123 | 43 | 46 | 24 | 36 |
| MIR1125 | 18 | 10 | 3 | 13 |
| MIR1127 | 297 | 204 | 101 | 99 |
| MIR1128 | 737 | 512 | 205 | 293 |
| MIR1129 | - | 6 | - | 2 |
| MIR1130 | 318 | 336 | 225 | 217 |
| MIR1131 | 5 | 12 | 5 | 2 |
| MIR1133 | 13 | 18 | 3 | 10 |
| MIR1135 | 292 | 222 | 97 | 154 |
| MIR1136 | 226 | 184 | 74 | 136 |
| MIR1137 | 13 | 30 | 14 | 40 |
| MIR1138 | - | - | - | 3 |
| MIR1139 | 1 | 4 | 1 | 12 |
| MIR1847 | 87 | 132 | 54 | 79 |
| MIR5048 | 1592 | 2180 | 488 | 4746 |
| MIR5049 | 7 | 10 | 1 | 2 |
| MIR5050 | 9 | 6 | 7 | 14 |
| MIR5062 | 79 | 132 | 17 | 61 |
| MIR5084 | 7 | 6 | 5 | 11 |
| MIR5175 | 39 | 36 | 22 | 42 |
| MIR5200 | 30 | 22 | 24 | 57 |
| MIR5384 | 439 | 1276 | 181 | 88 |
| MIR6197 | 3 | 2 | 1 | 6 |
| MIR7757 | 254 | 452 | 191 | 951 |
| MIR9652 | - | - | 2 | 17 |
| MIR9653 | 9733 | 12450 | 4324 | 5245 |
| MIR9654 | 3 | 4 | 1 | 11 |
| MIR9655 | 1 | - | - | - |
| MIR9656 | 66 | 46 | 21 | 45 |
| MIR9657 | 2 | 2 | 372 | 431 |
| MIR9658 | 147 | 124 | 90 | 222 |
| MIR9659 | - | - | - | 1 |
| MIR9660 | - | 4 | - | 3 |
| MIR9661 | 1 | 2 | 1 | 3 |
| MIR9662 | 21637 | 35398 | 13304 | 17284 |
| MIR9663 | - | - | 1 | 18 |
| MIR9664 | 2617 | 4194 | 226 | 1424 |
| MIR9665 | 1 | - | 2 | 2 |
| MIR9666 | 30 | 22 | 24 | 58 |
| MIR9668 | 365 | 324 | 112 | 203 |
| MIR9669 | 332 | 278 | 173 | 889 |
| MIR9670 | 65 | 118 | 23 | 788 |
| MIR9671 | 11 | 2 | 4 | 2 |
| MIR9672 | 366 | 756 | 182 | 2976 |
| MIR9673 | 2 | 2 | - | 12 |
| MIR9674 | 3002 | 3910 | 1785 | 5834 |
| MIR9675 | 20 | 86 | 9 | 412 |
| MIR9676 | 144 | 428 | 88 | 403 |
| MIR9677 | 5 | 4 | 2 | 5 |
| MIR9678 | - | - | - | 1 |
| MIR9679 | 1 | - | 1 | 1 |
| MIR9772 | 29 | 38 | 22 | 139 |
| MIR9773 | 1 | - | 1 | 1 |
| MIR9774 | 7 | - | - | 7 |
| MIR9775 | 1 | - | - | 1 |
| MIR9776 | 167 | 272 | 127 | 325 |
| MIR9777 | 439 | 830 | 341 | 421 |
| MIR9778 | 503 | 384 | 422 | 4445 |
| MIR9779 | 540 | 1276 | 309 | 1071 |
| MIR9780 | 3 | 8 | 1 | - |
| MIR9781 | - | - | - | 1 |
| MIR9782 | 1 | - | - | 5 |
| MIR9783 | 7 | 4 | 2 | 6 |
