# Supplementary material for: Novel and conserved drought-responsive microRNAs expression analysis in root tissues of wheat (Triticum asetivum L.) at reproductive stage
Source: Front Plant Sci. 2025 May 20;16:1581542. doi: 10.3389/fpls.2025.1581542 (PMC12129965; doi:10.3389/fpls.2025.1581542)
Supplement: Supplementary file 3 [file Table3.docx]

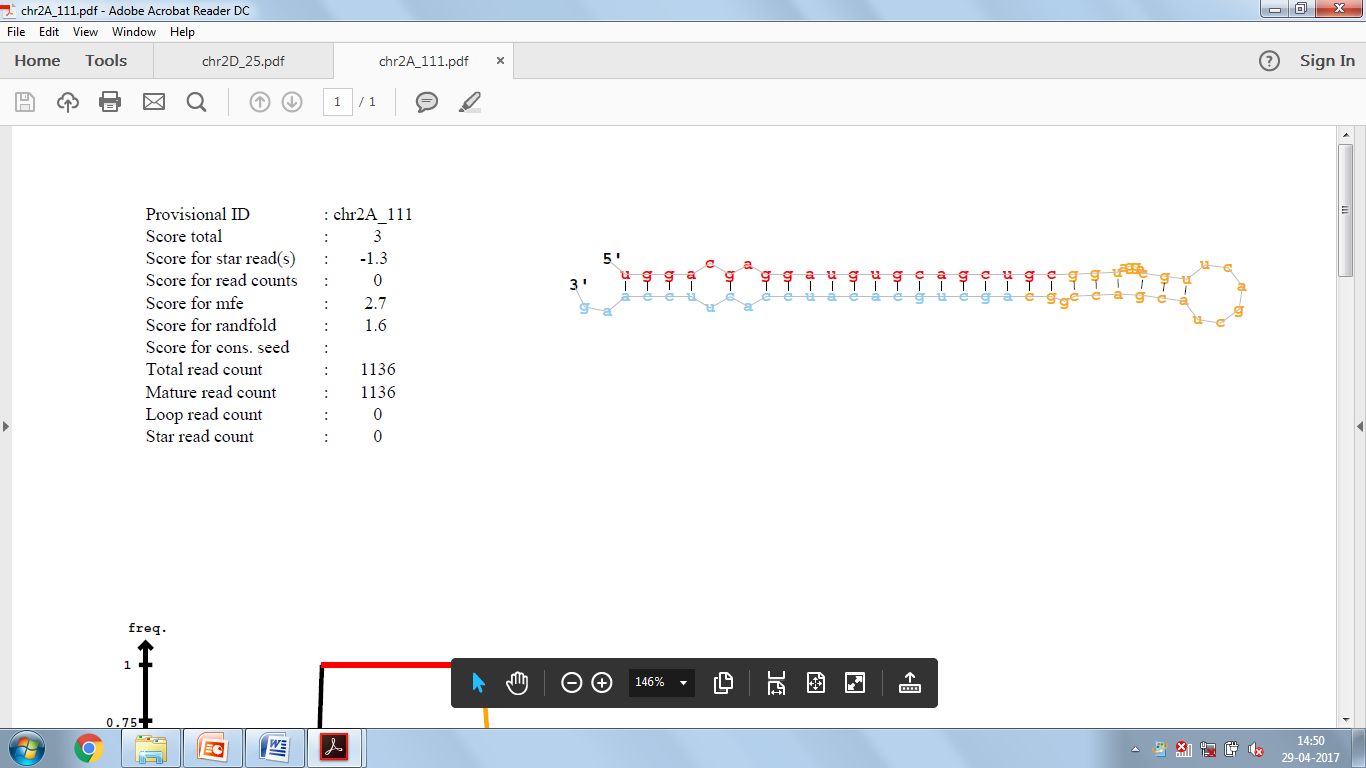


chr2A_111

chr3A_45

chr5B_55

chr5D_59

chr5D_87

chr5D_93

chr6D_56

chr2B_141

chr2D_25


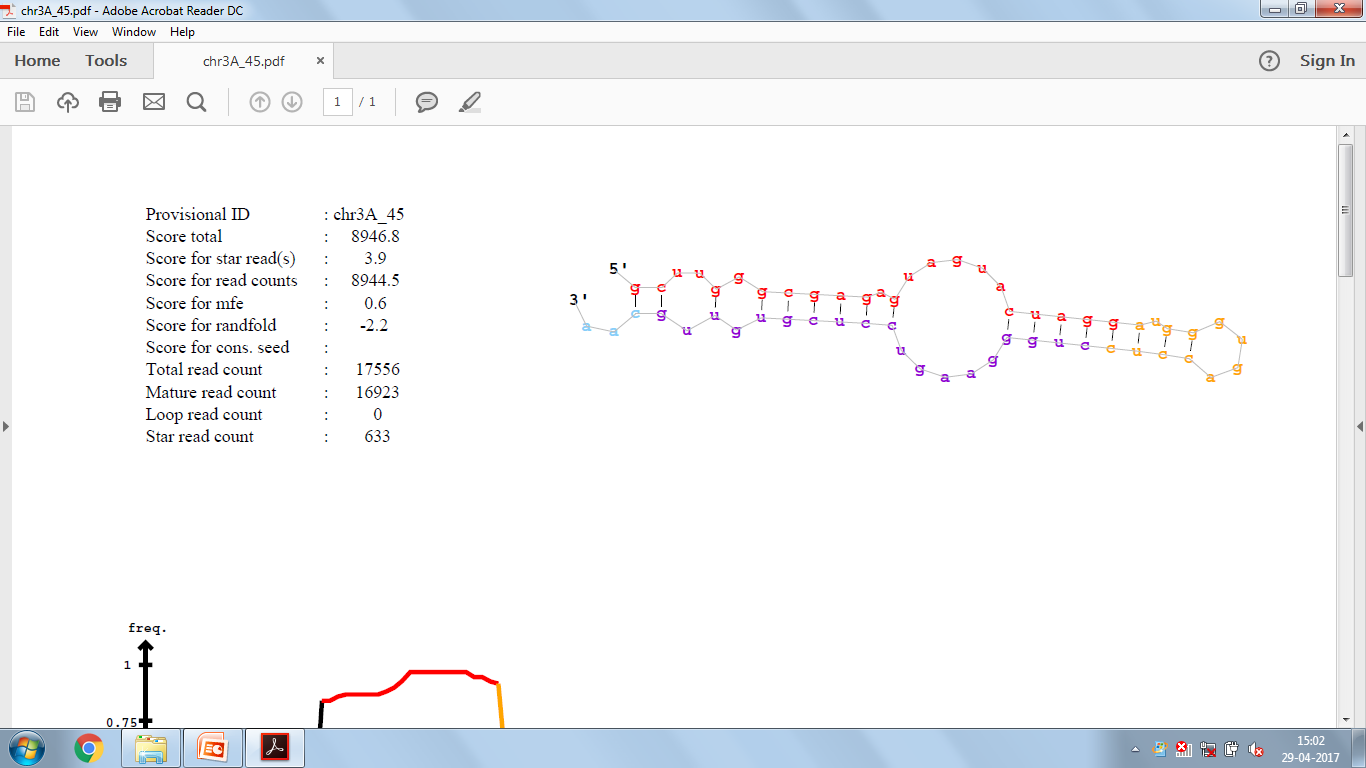


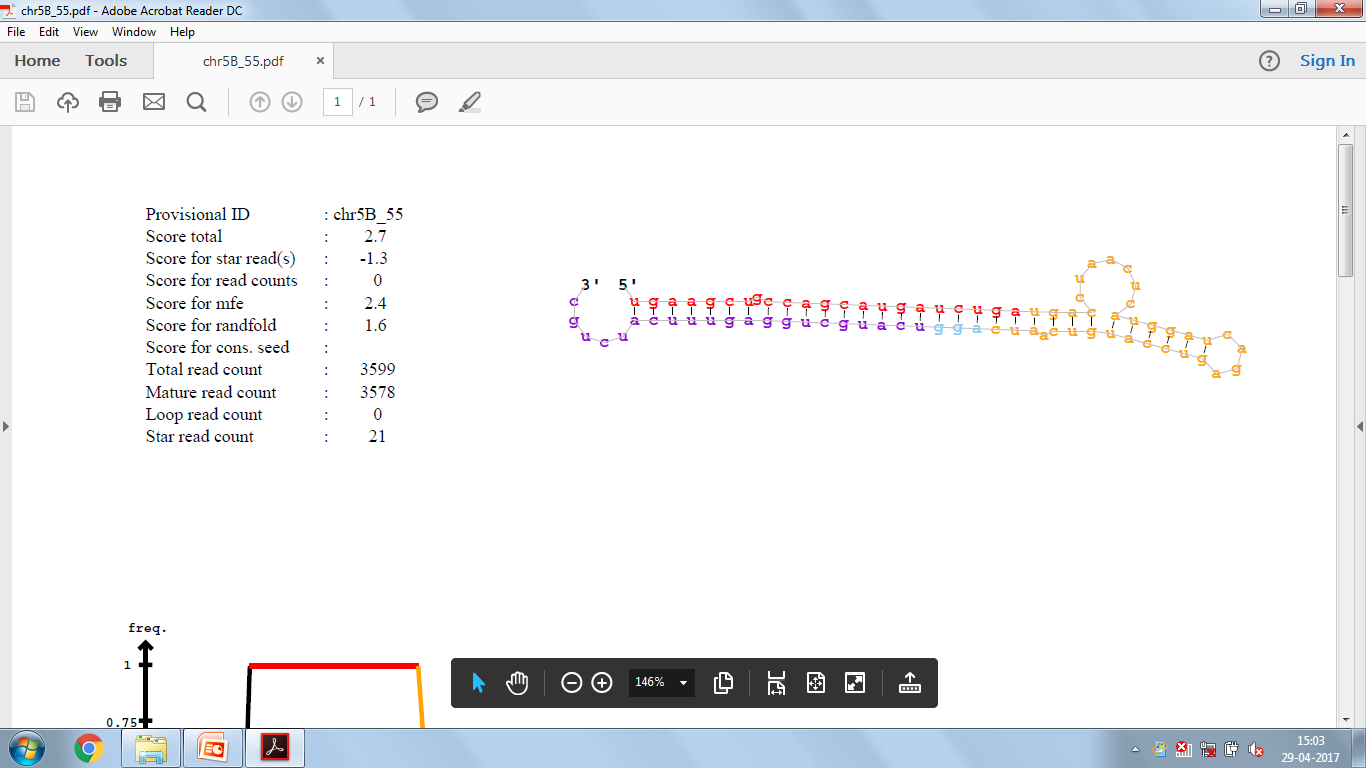


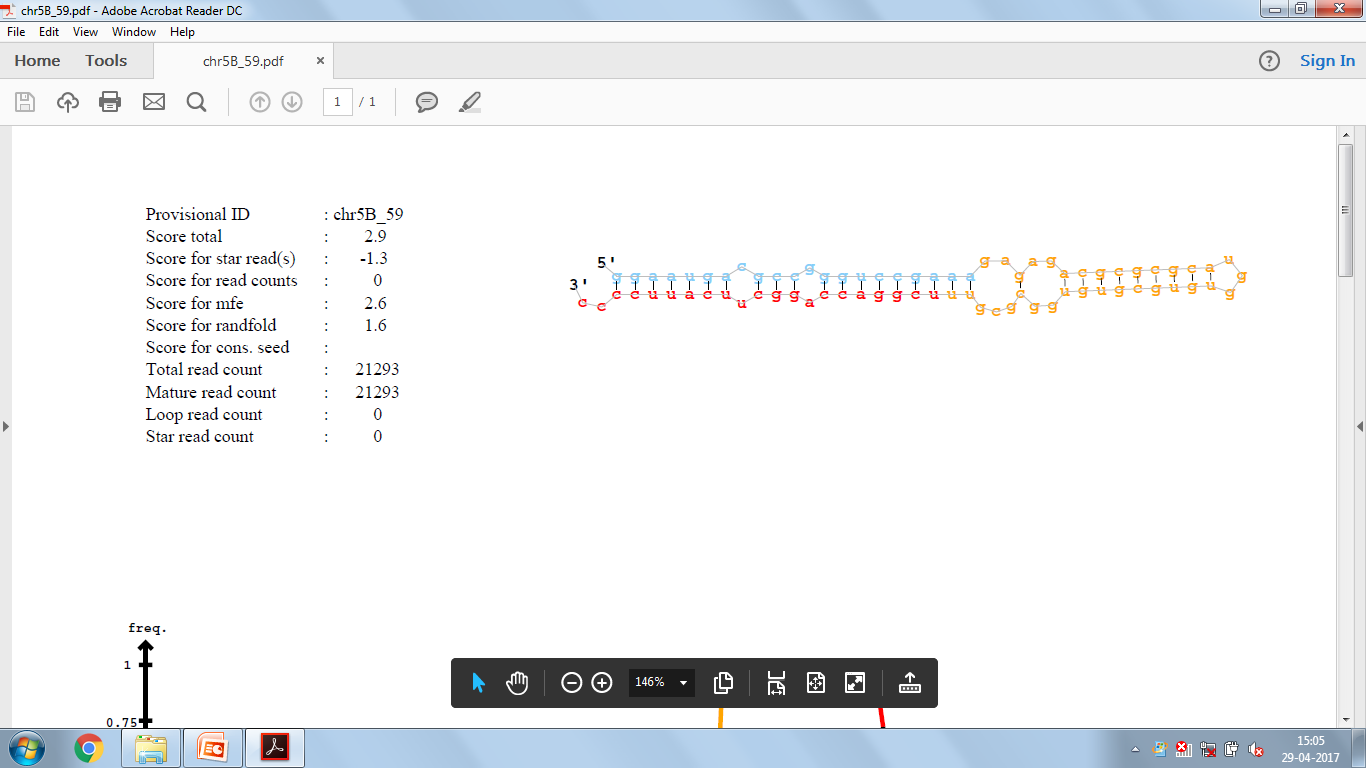


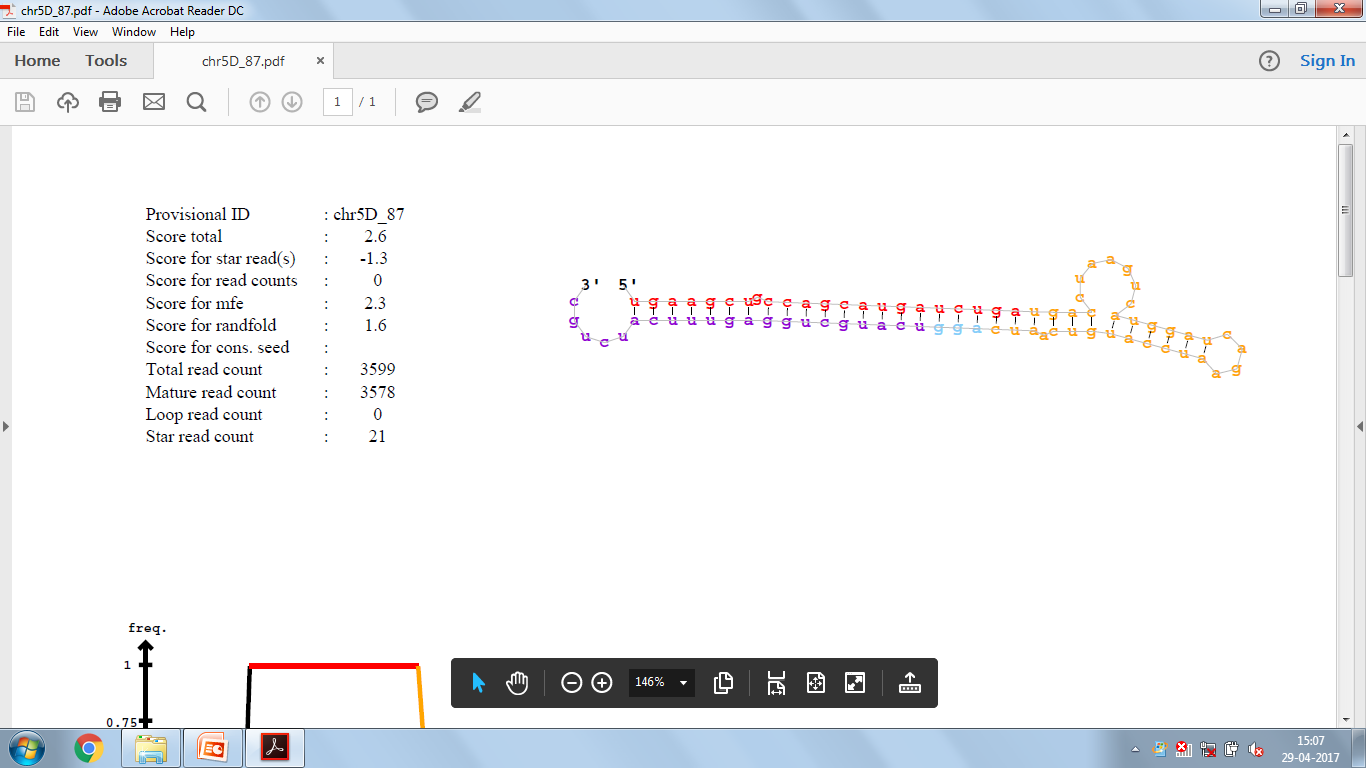


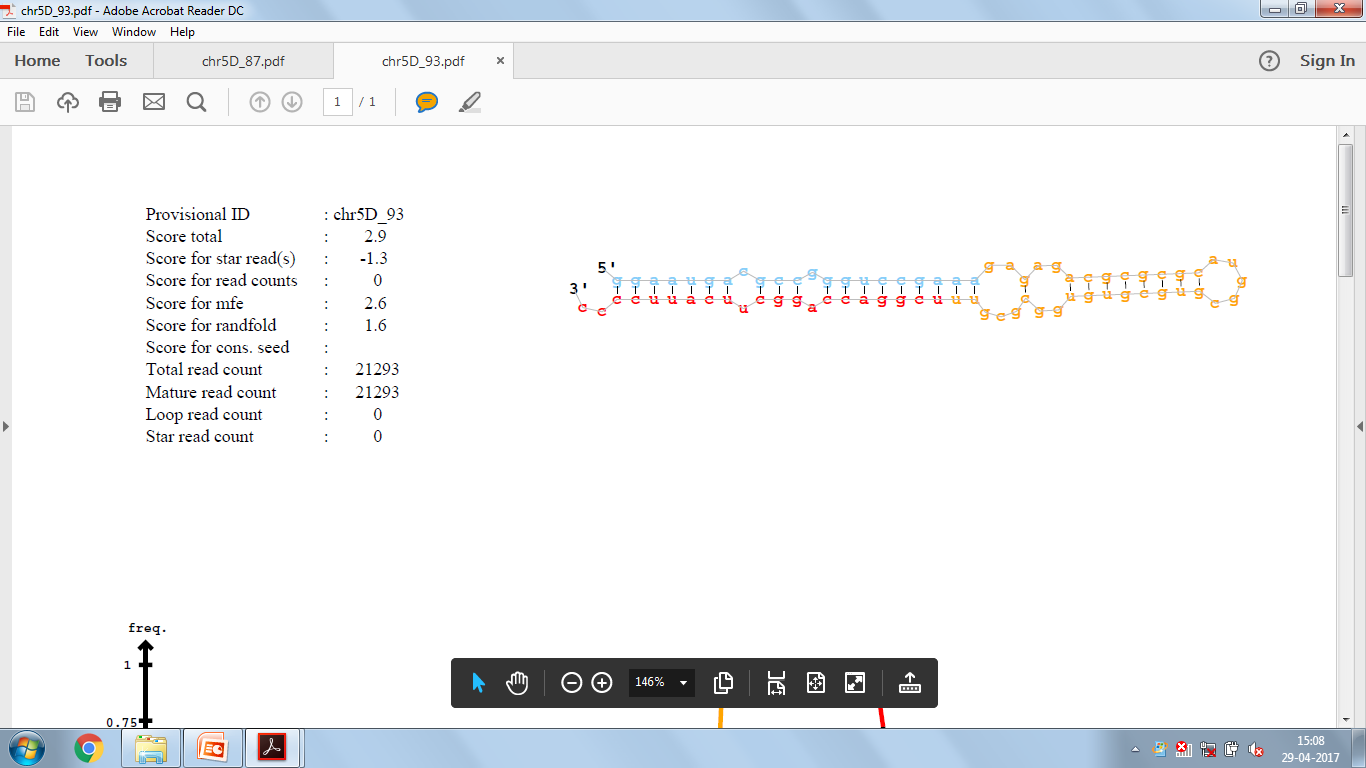


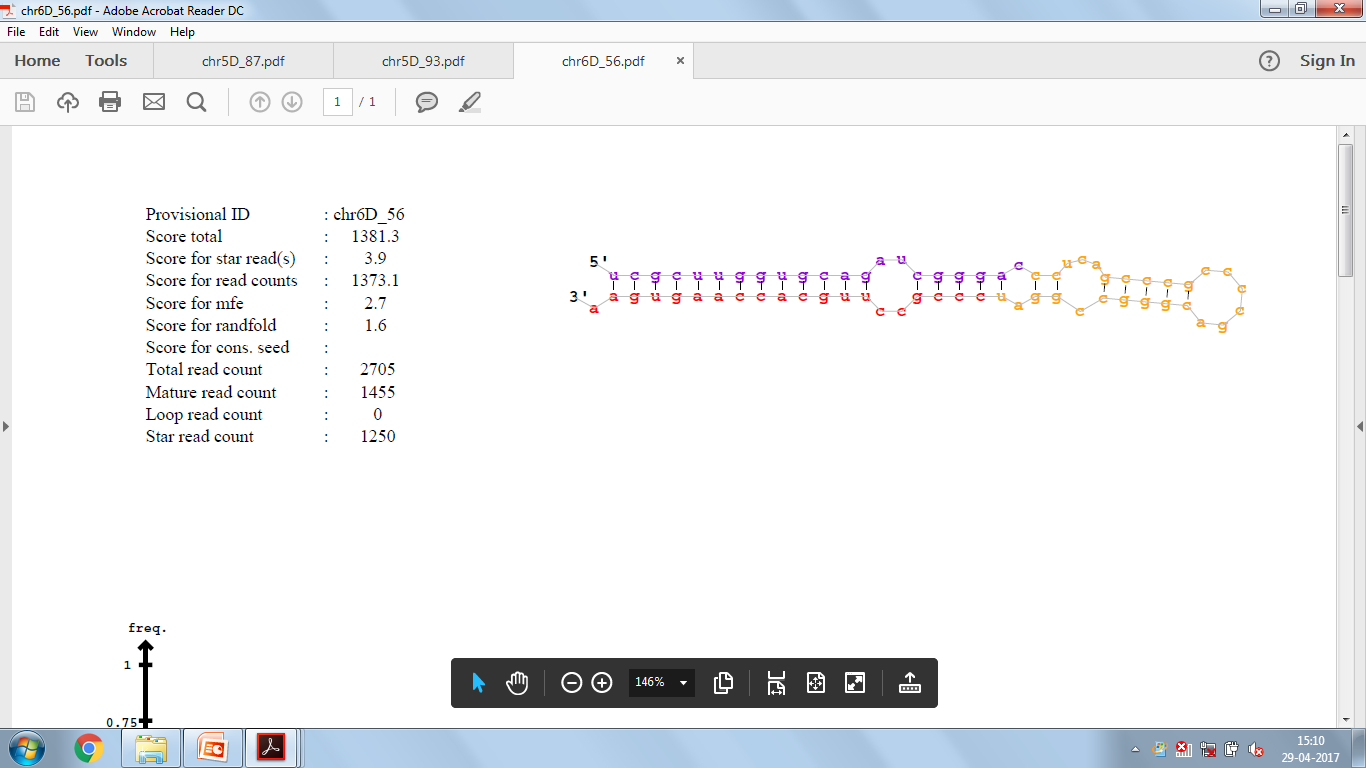


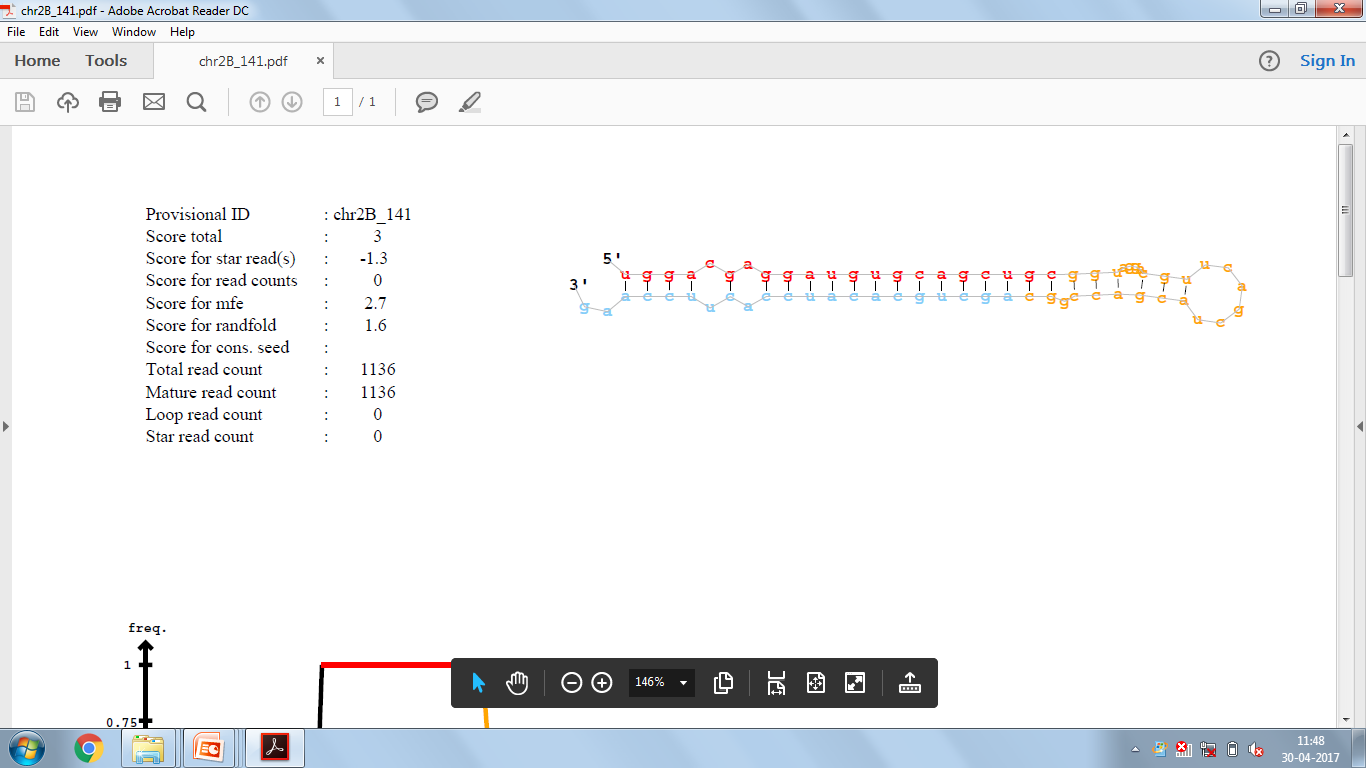


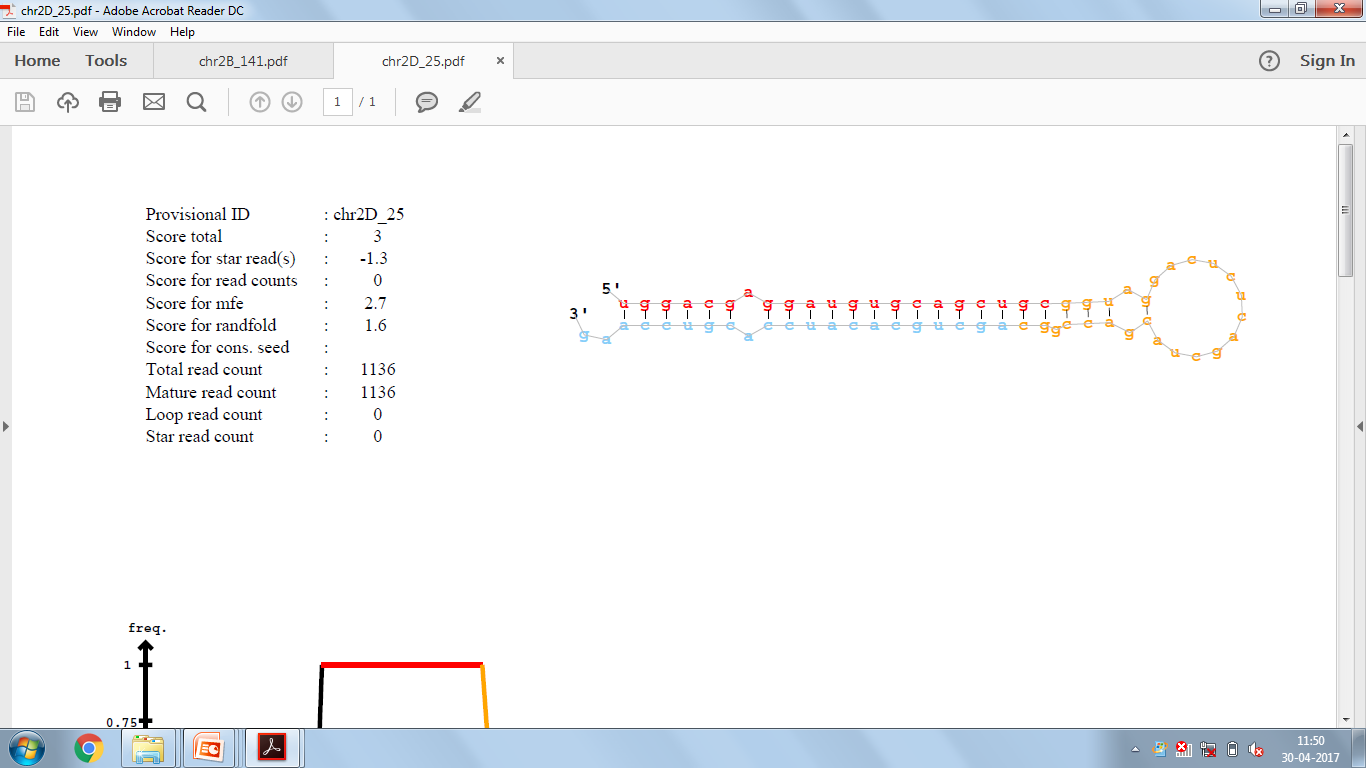


Supp. Fig. 1a: Structure of novel miRNAs identified in TC library.


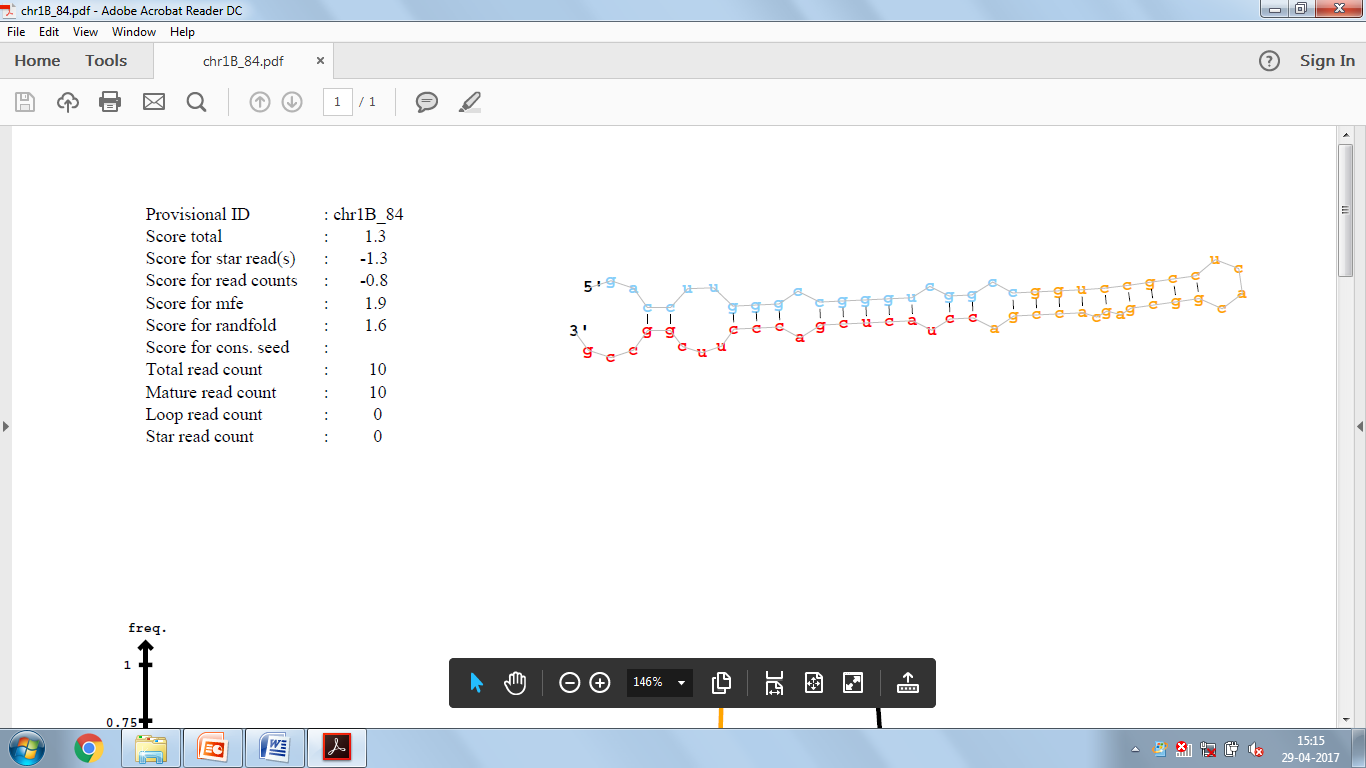


chr1B_84

chr2A_172

chr2A_187

chr2B_234

chr2B_265

chr2D_4

chr2D_47

chr3A_73

chr5B_103


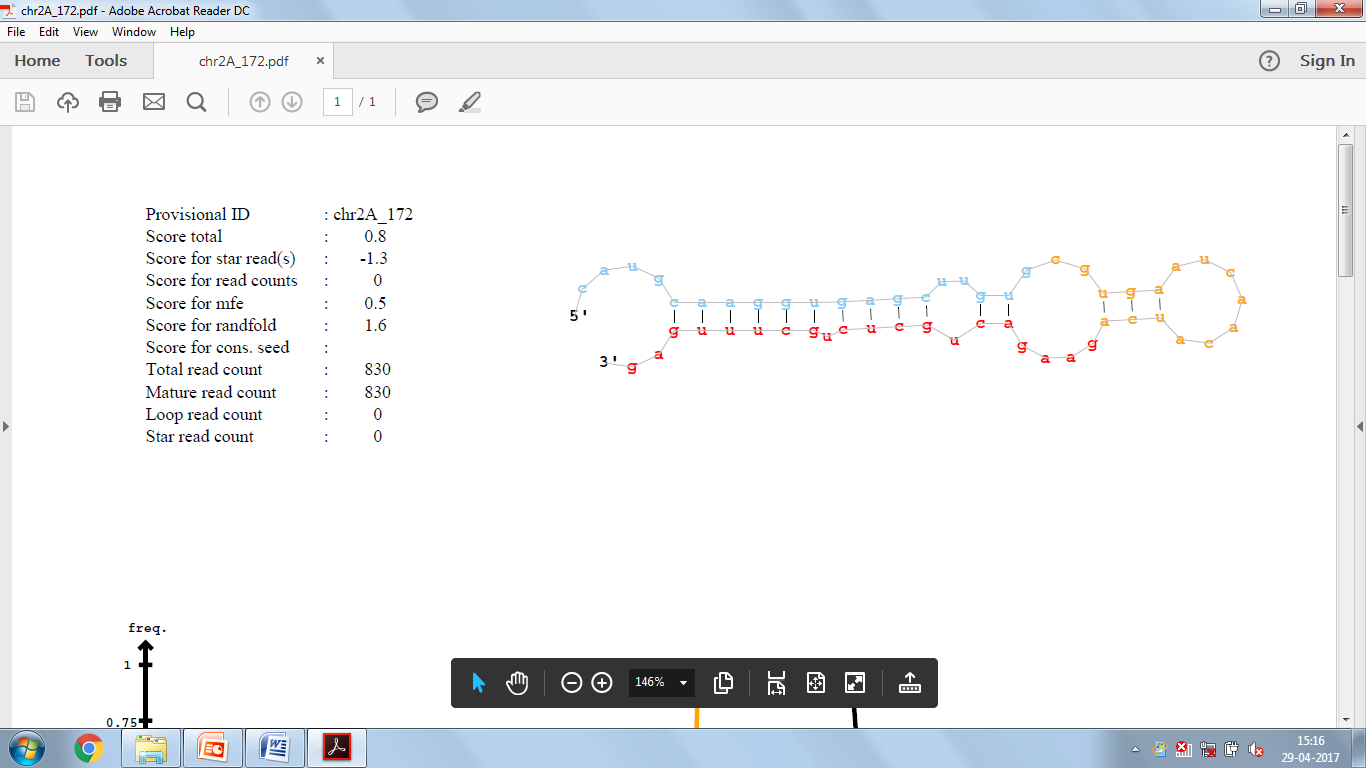


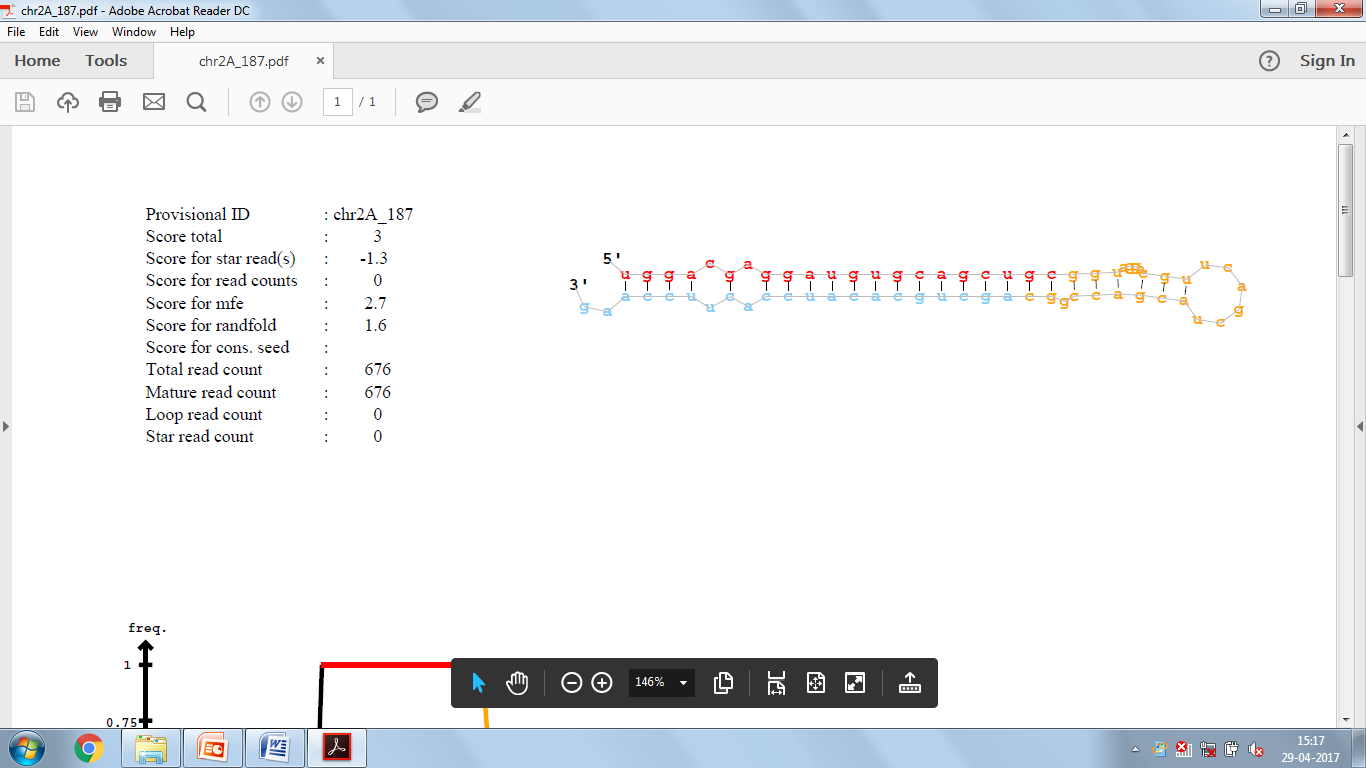


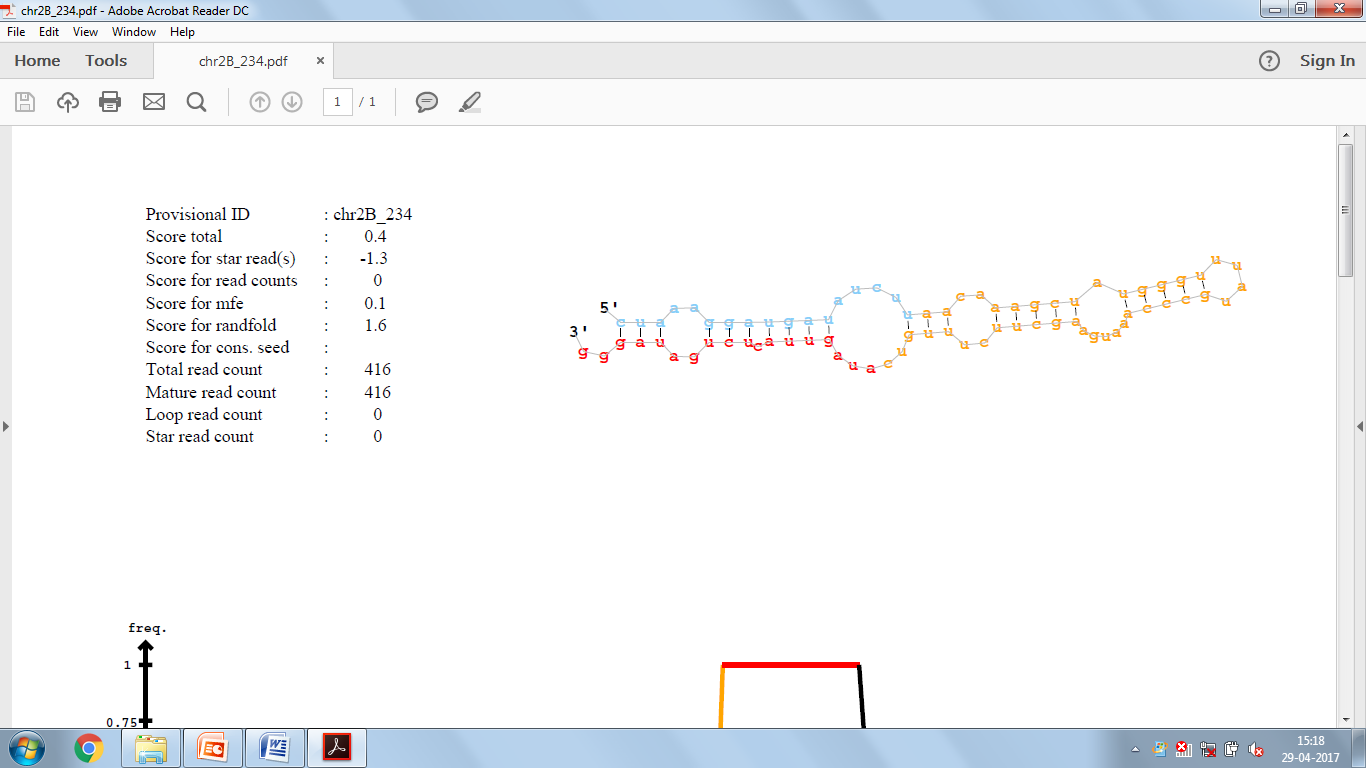


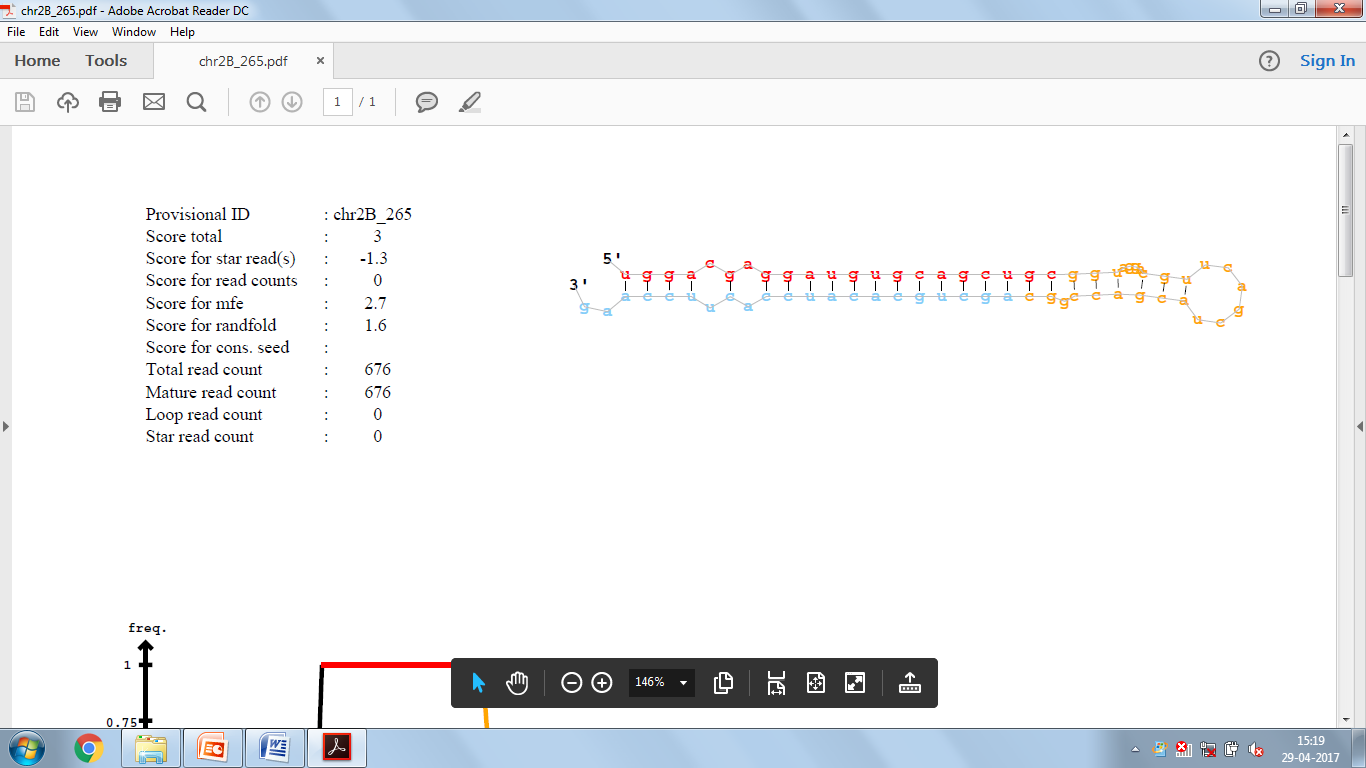


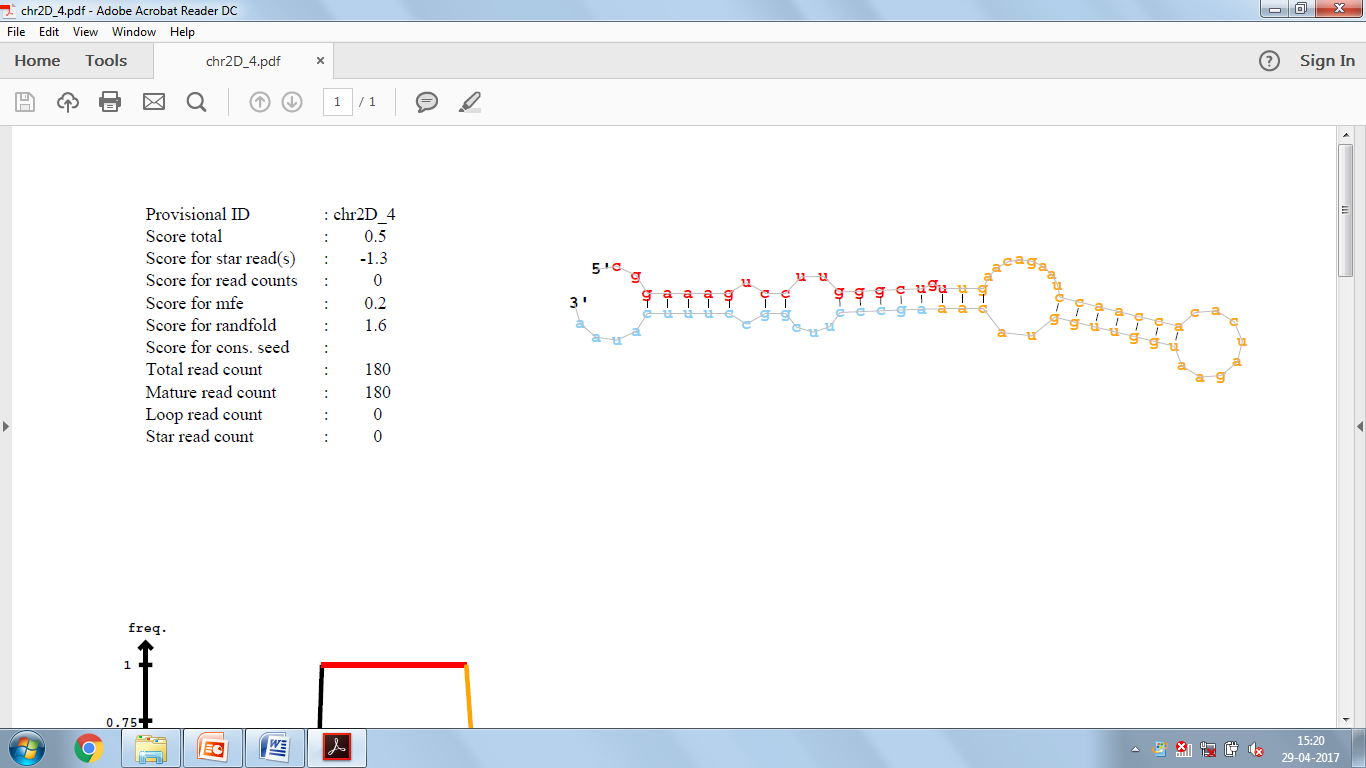


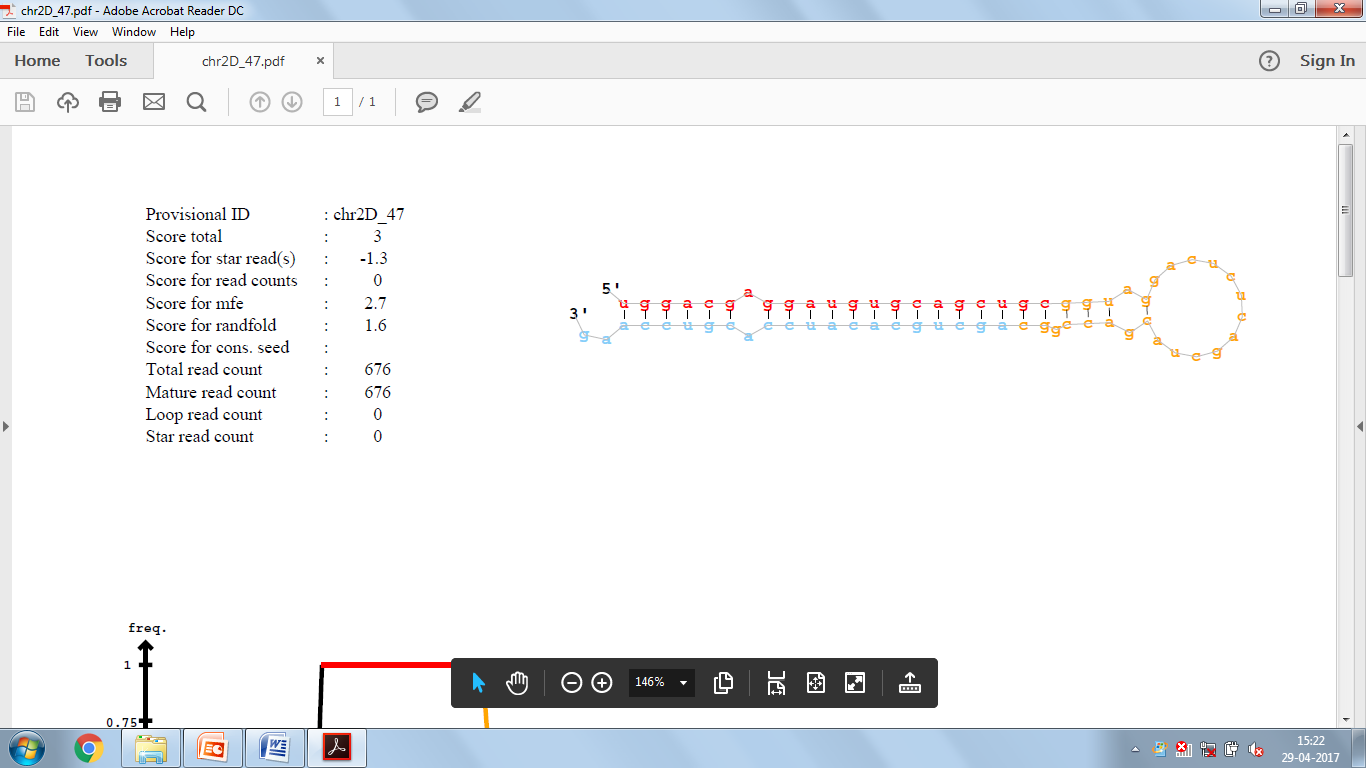


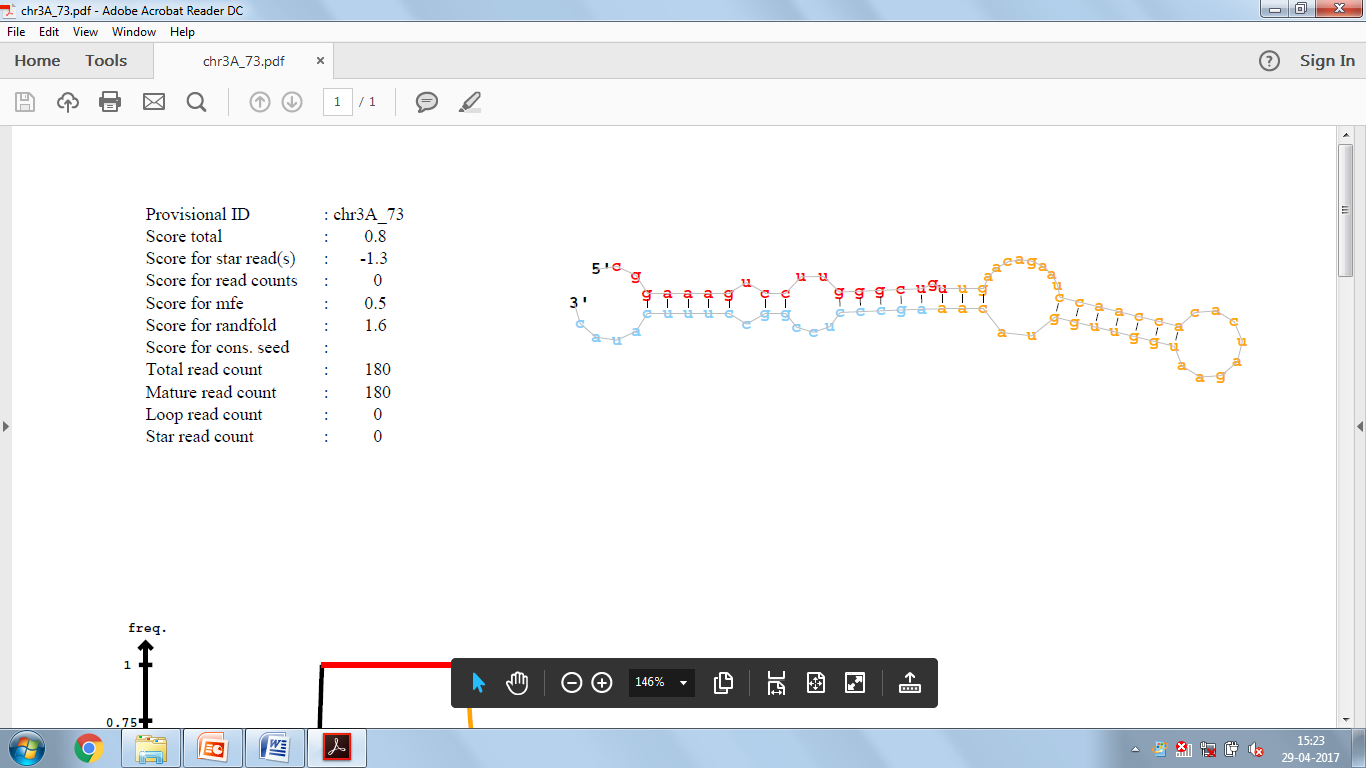


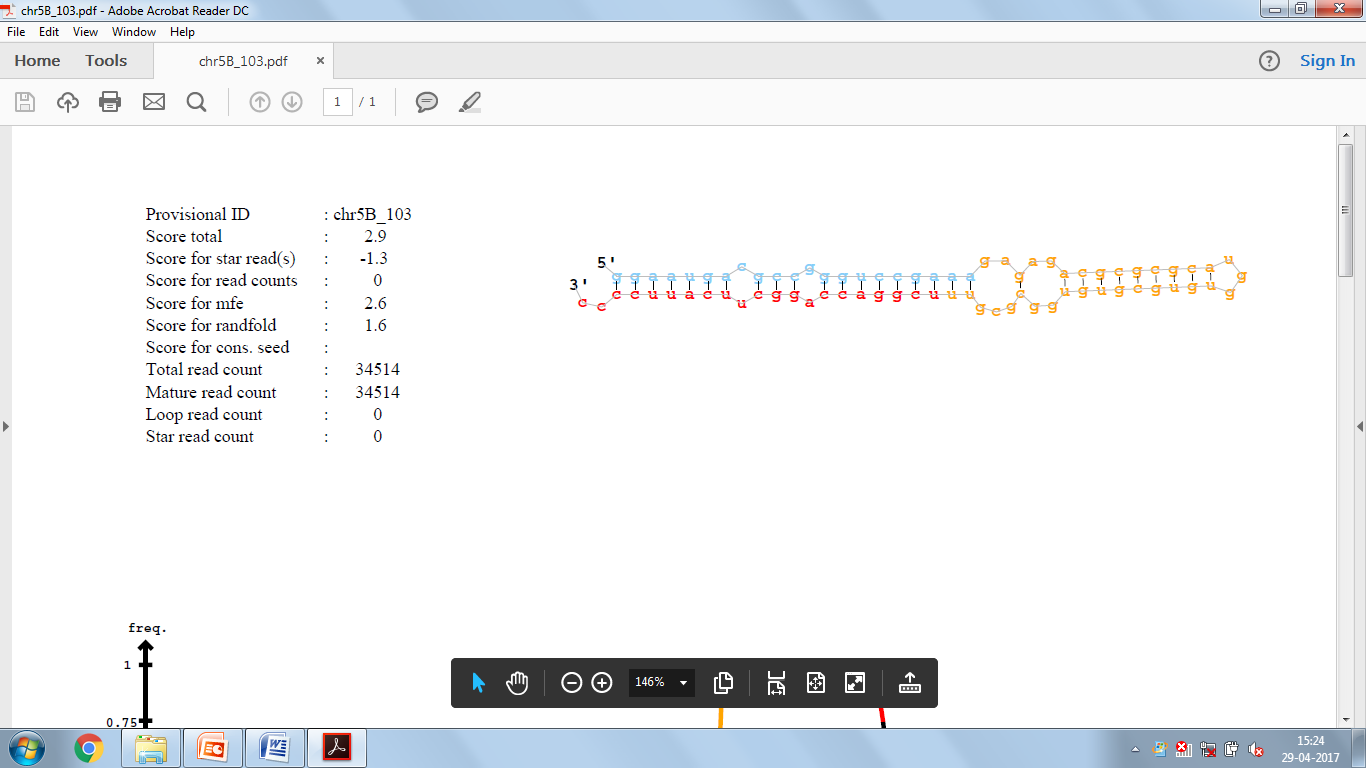


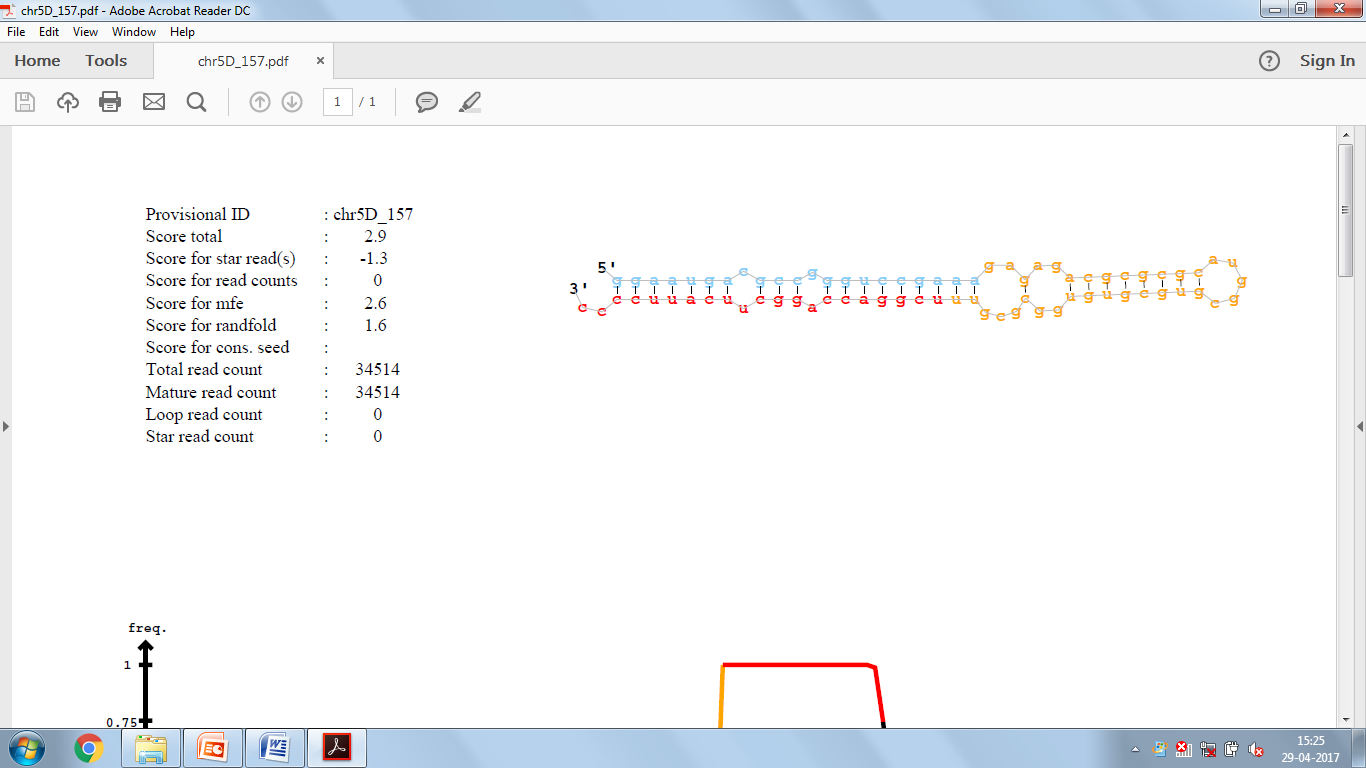


chr5D_157

chr6A_22

chr6B_39

chr6D_74

chr6D_93

chr7A_160

chr7B_191

chr7B_199


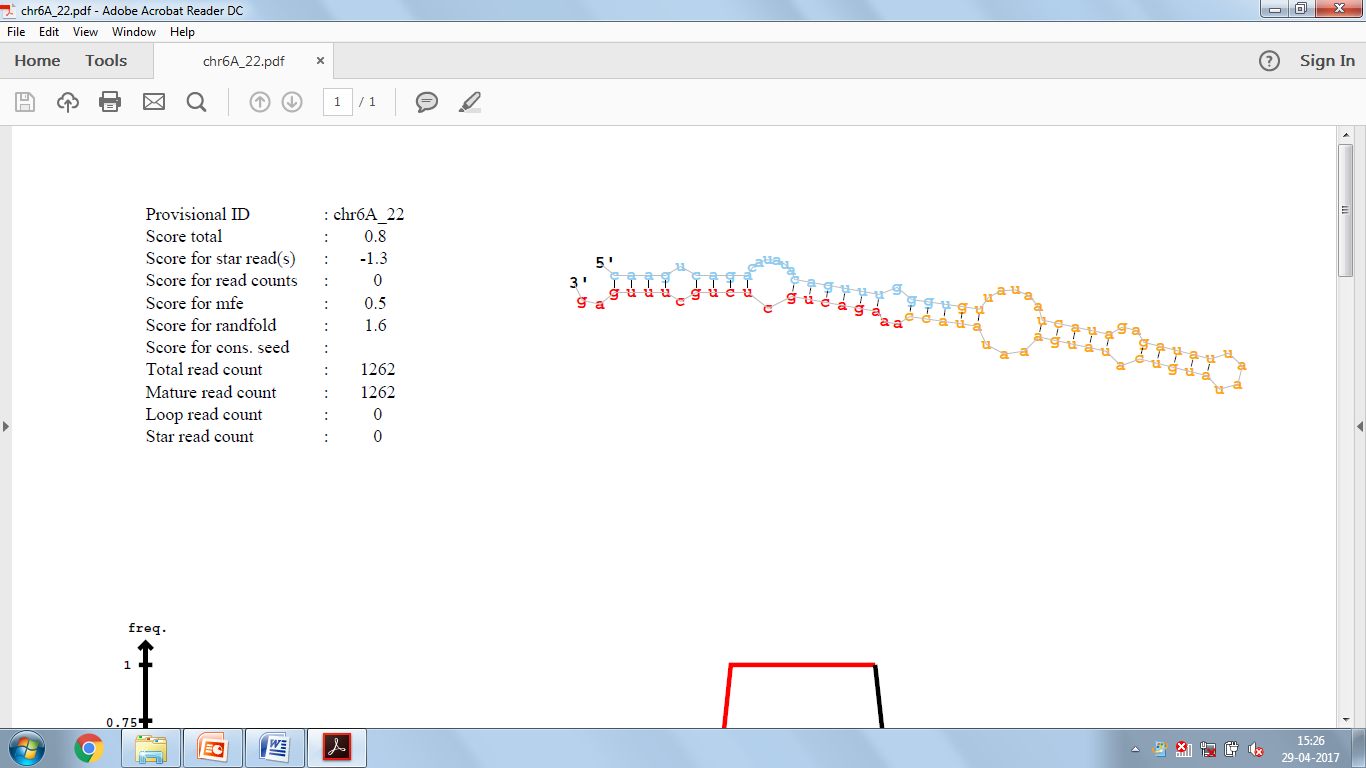


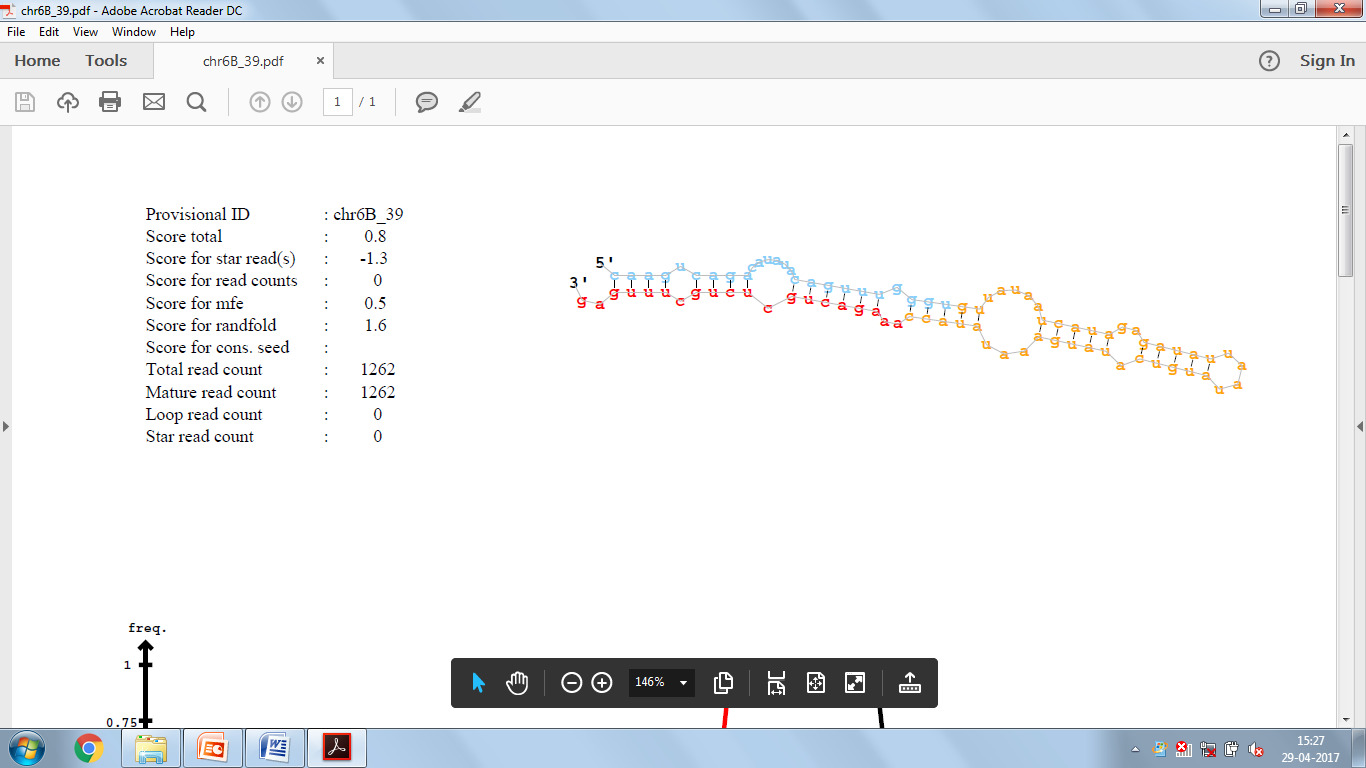


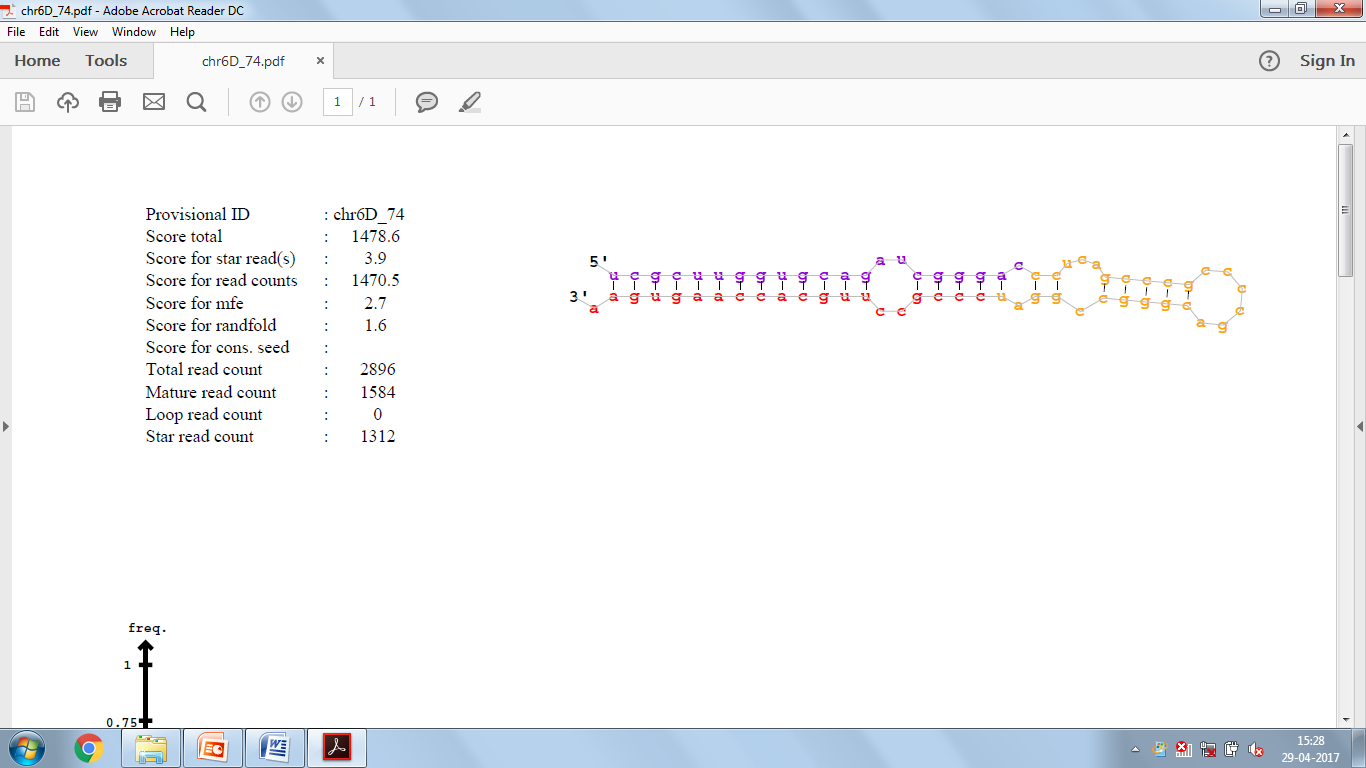


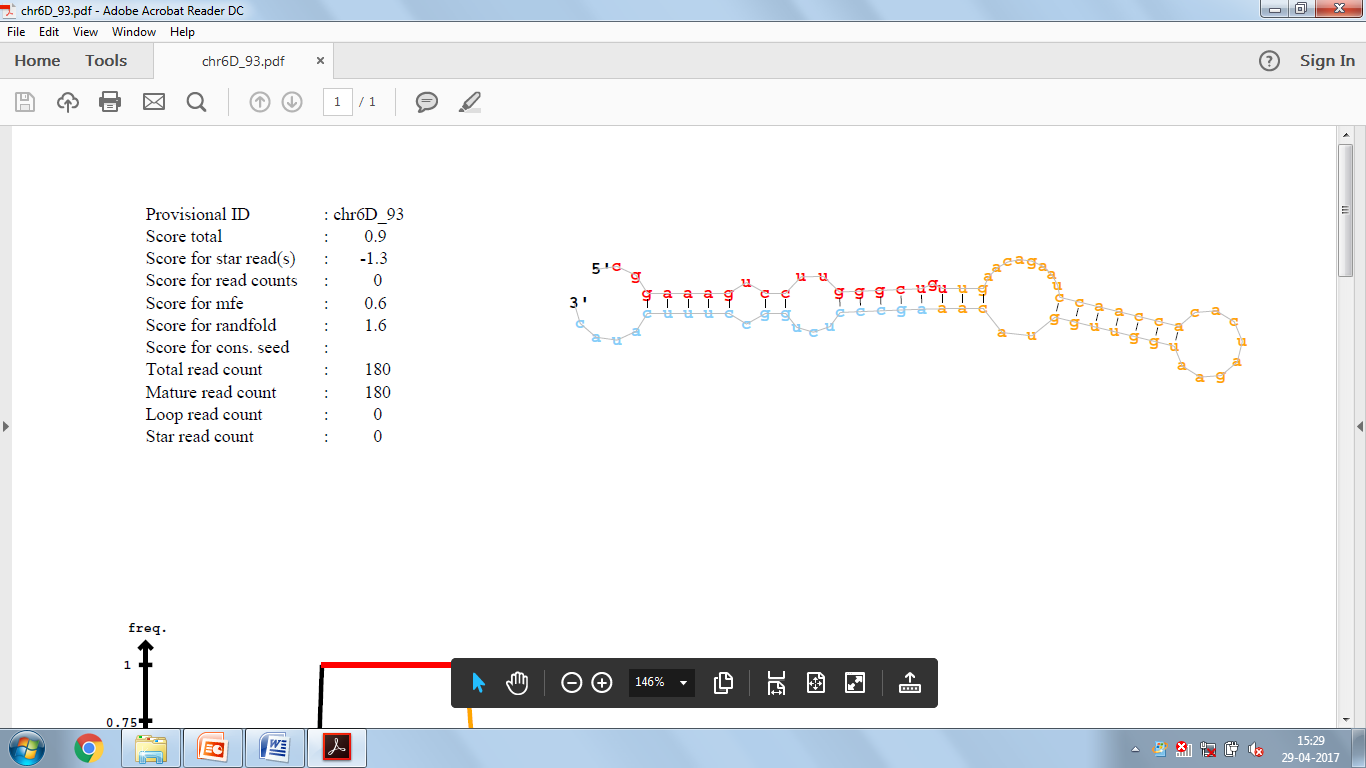


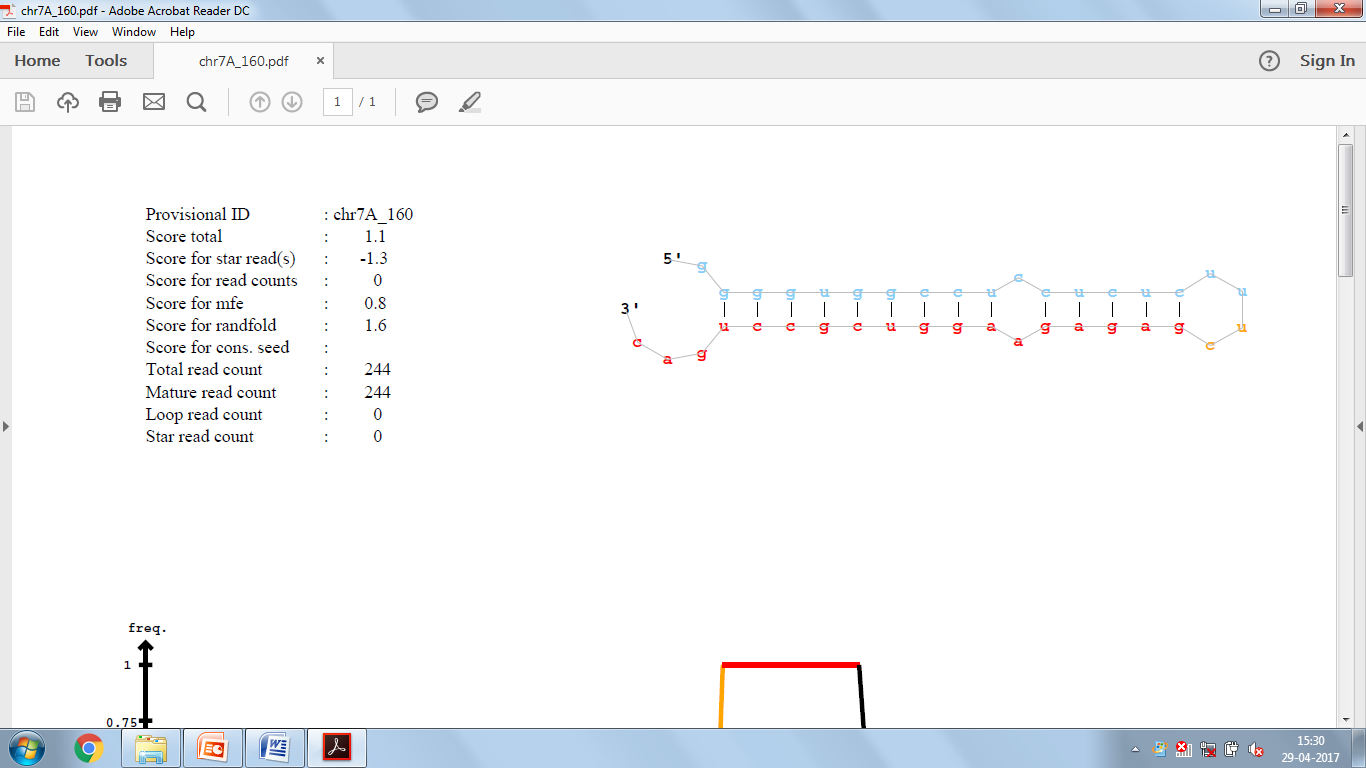


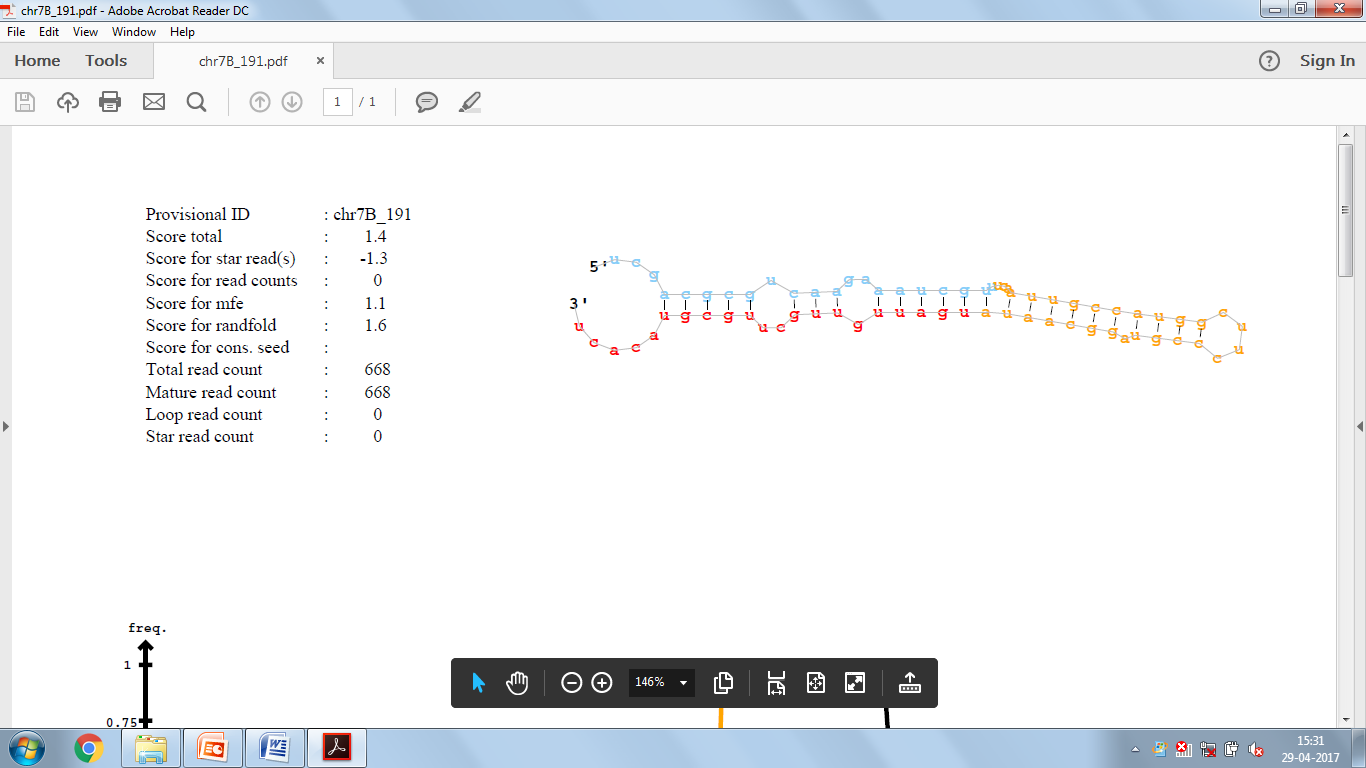


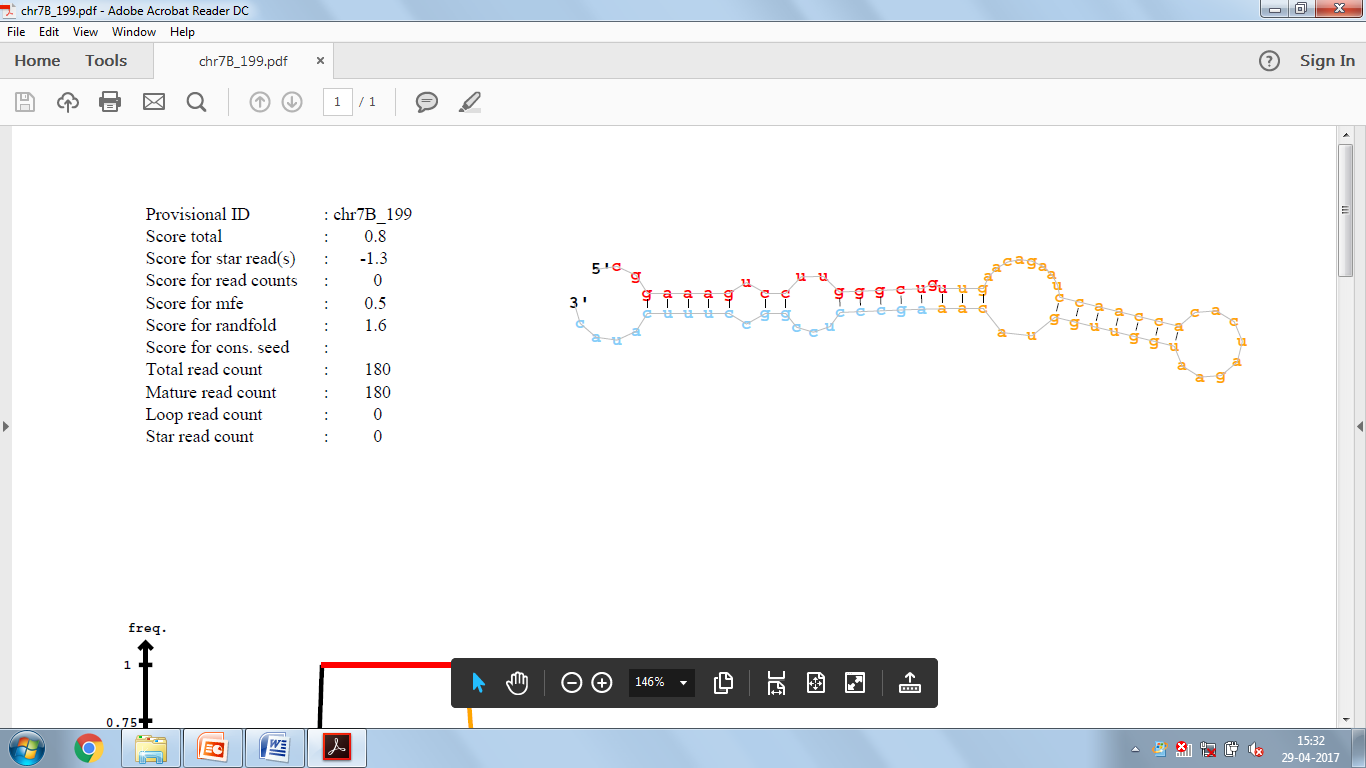


Supp. Fig. 1b: Structure of novel miRNAs identified in TD library.


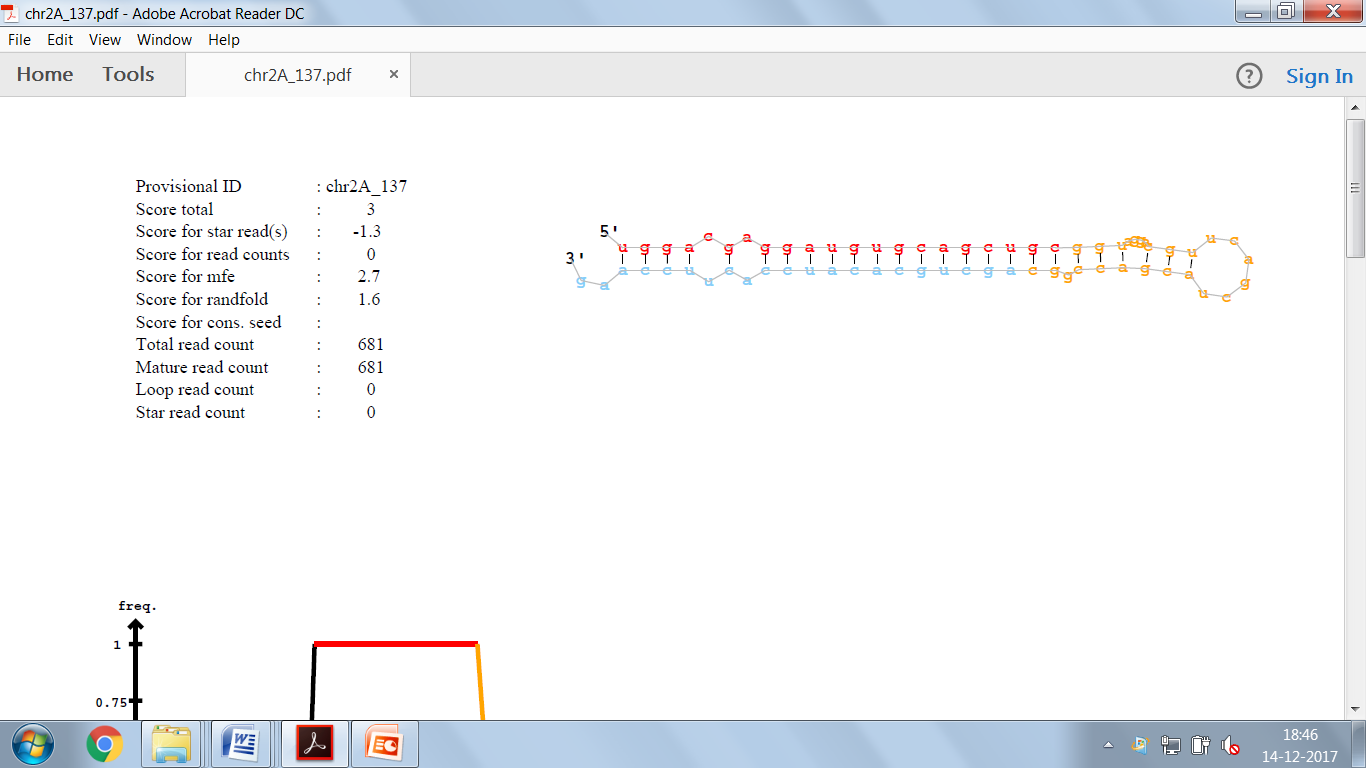


chr2A_137

chr2A_145

chr2B_164

chr2B_185

chr2D_19

chr3A_34

chr3B_101

chr3B_115

chr4A_172


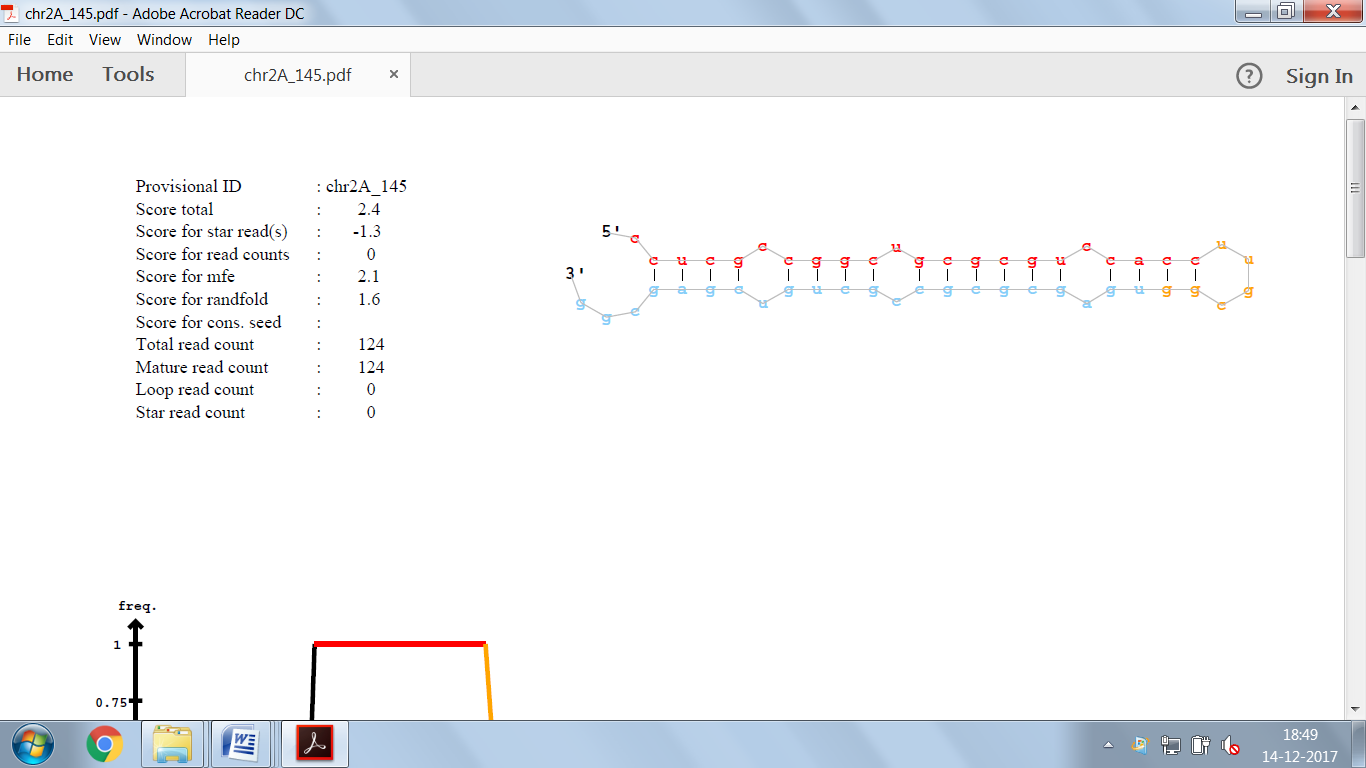


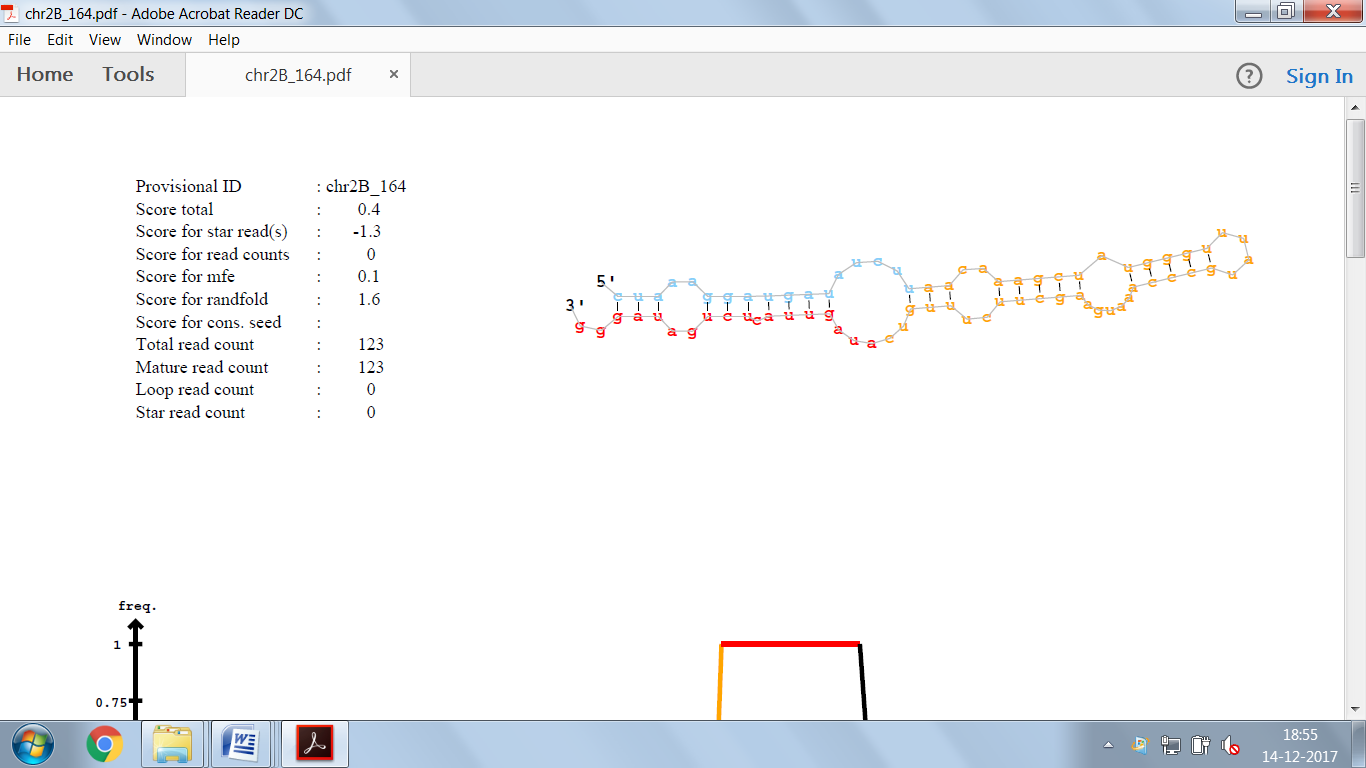


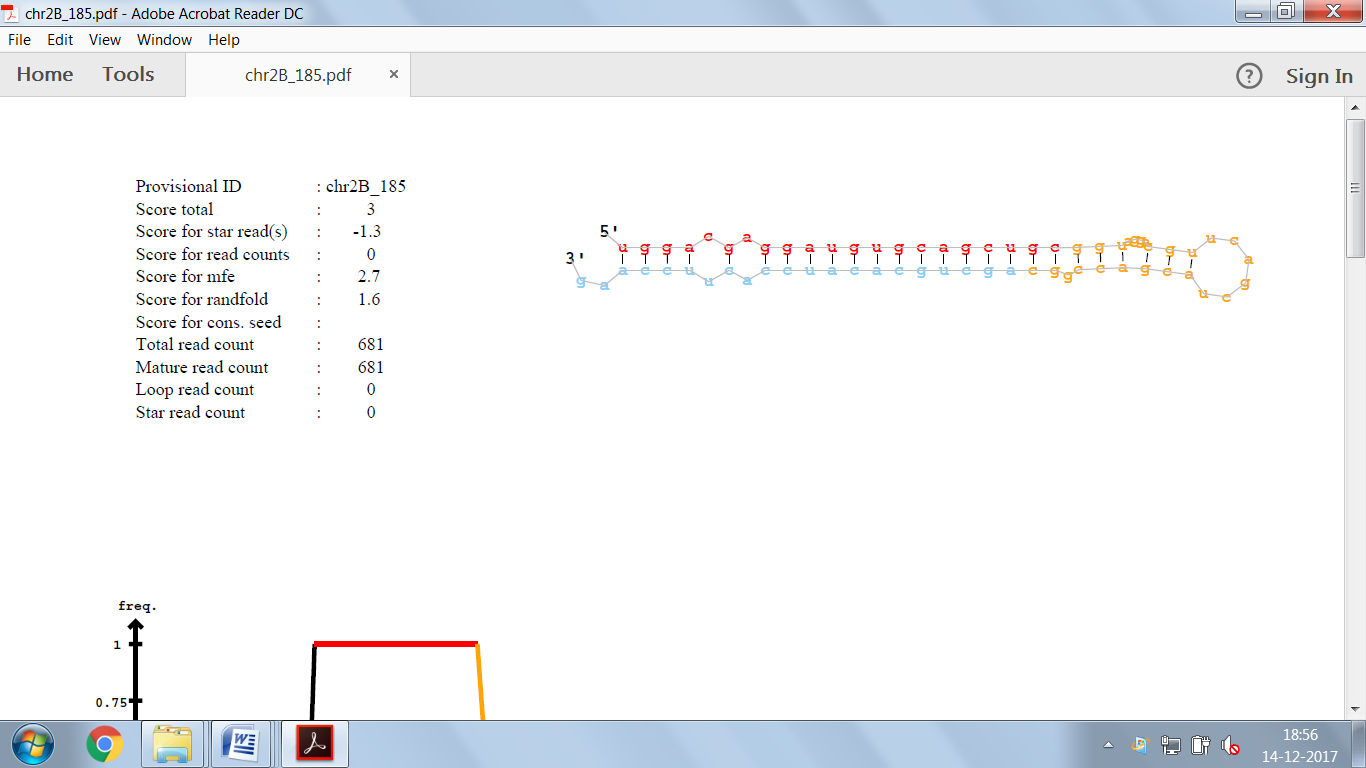


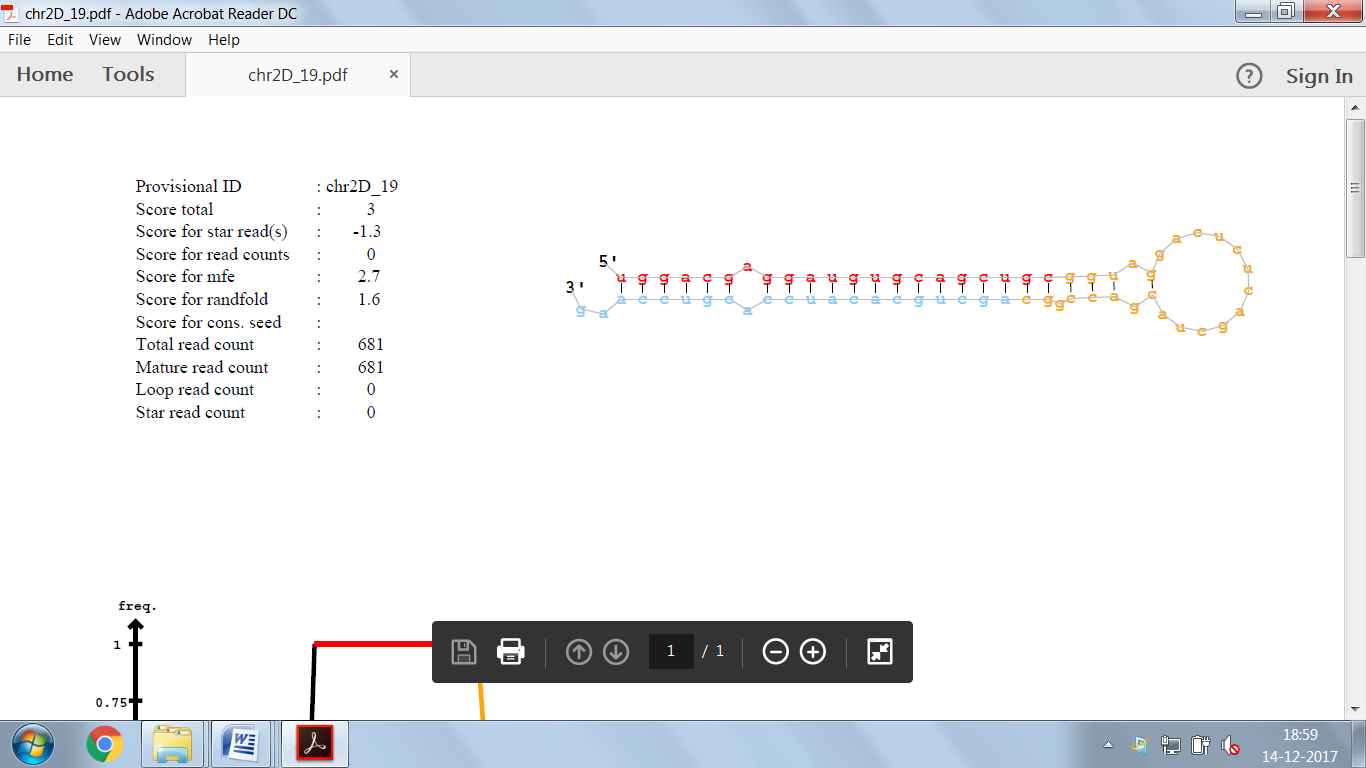


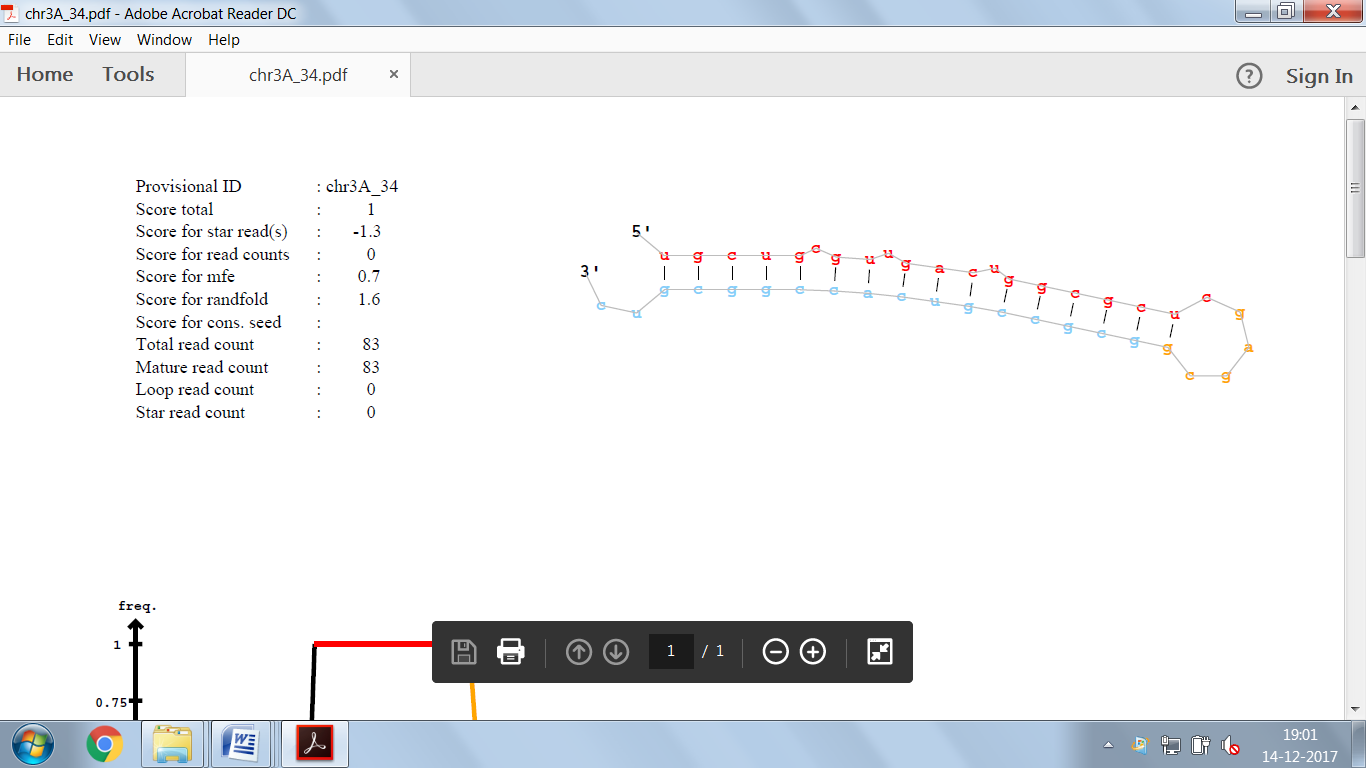


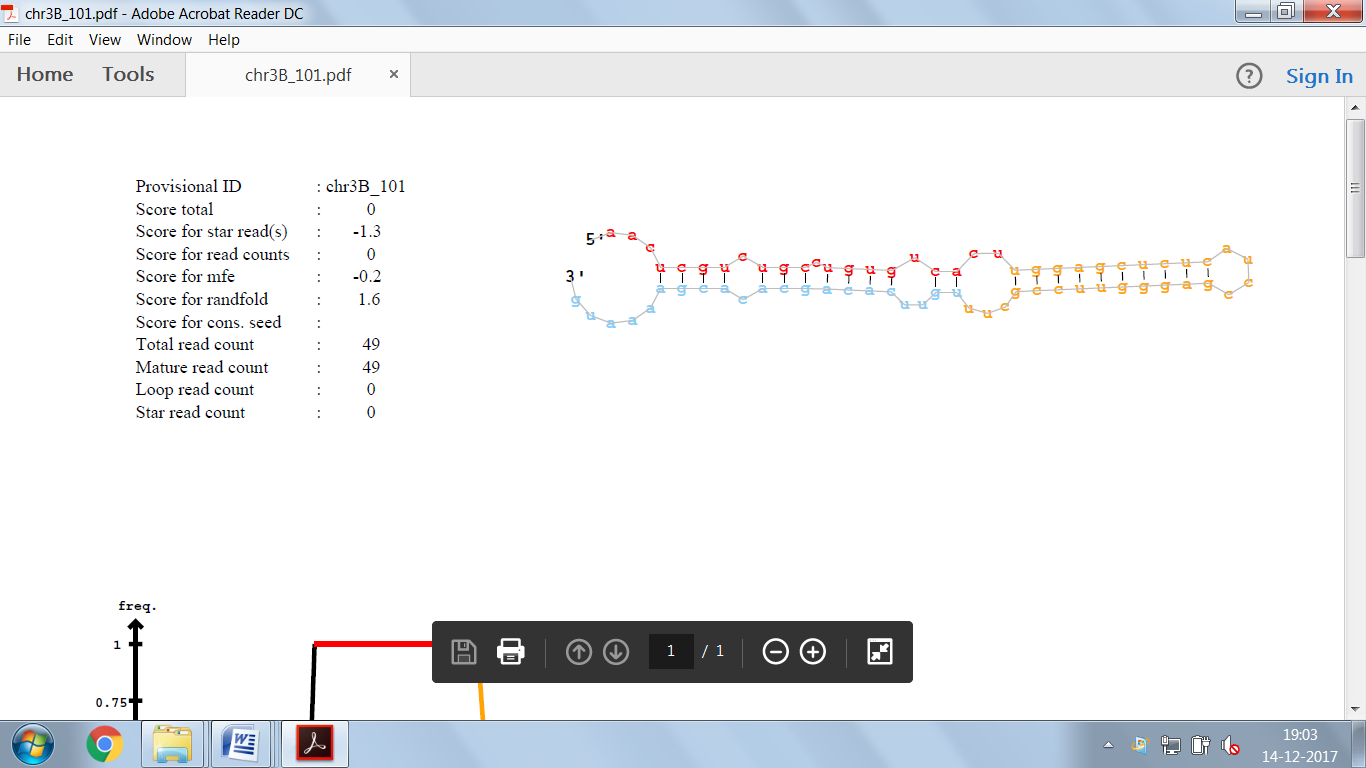


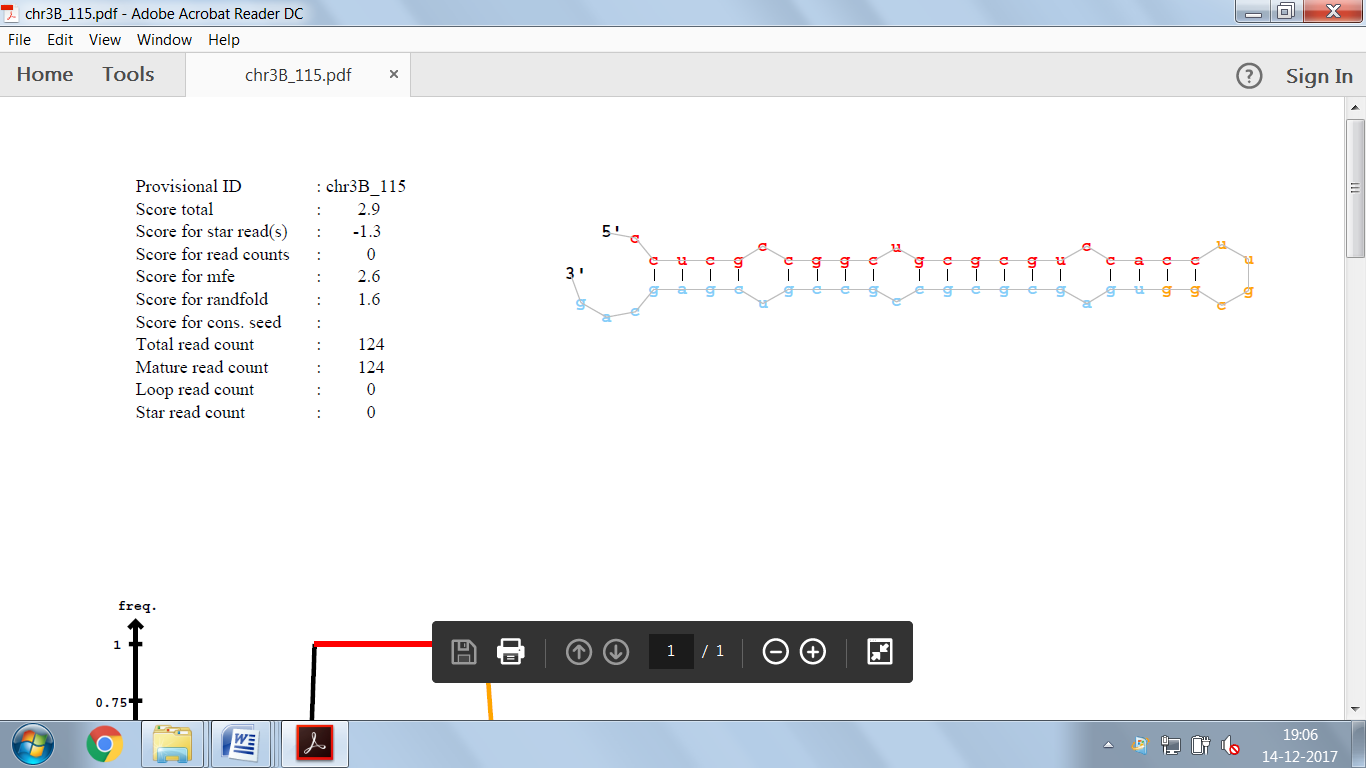


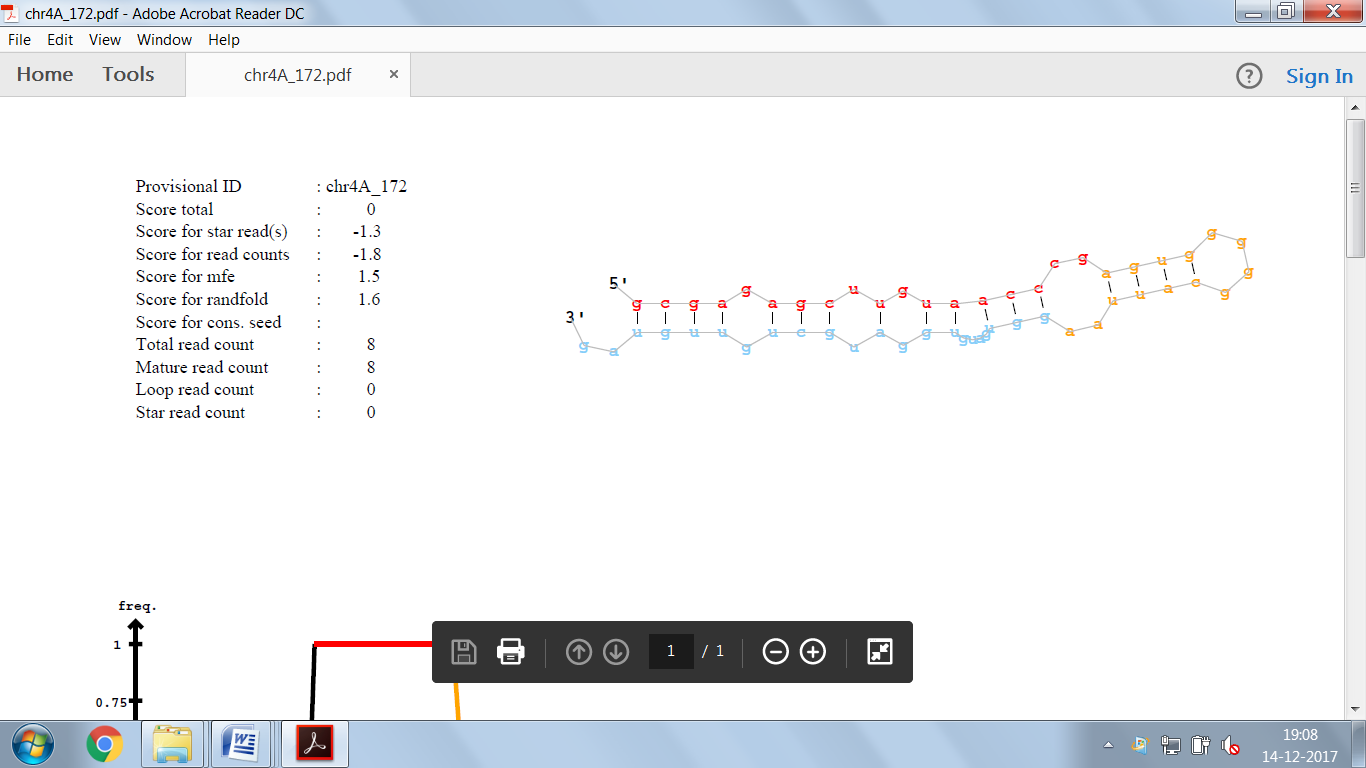


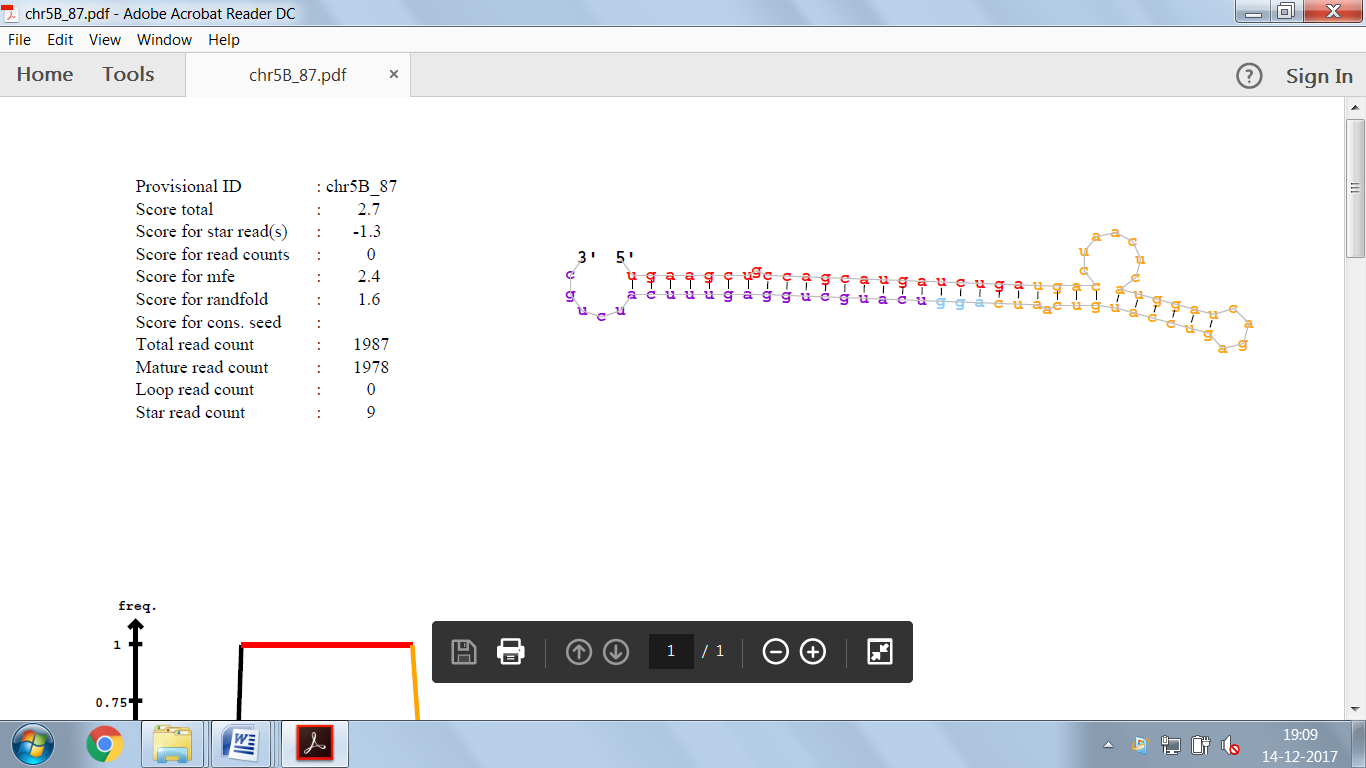


chr5B_87

chr5B_91

chr5B_94

chr5D_112

chr5D_125

chr5D_133

chr6B_43

chr6D_63

chr7B_161


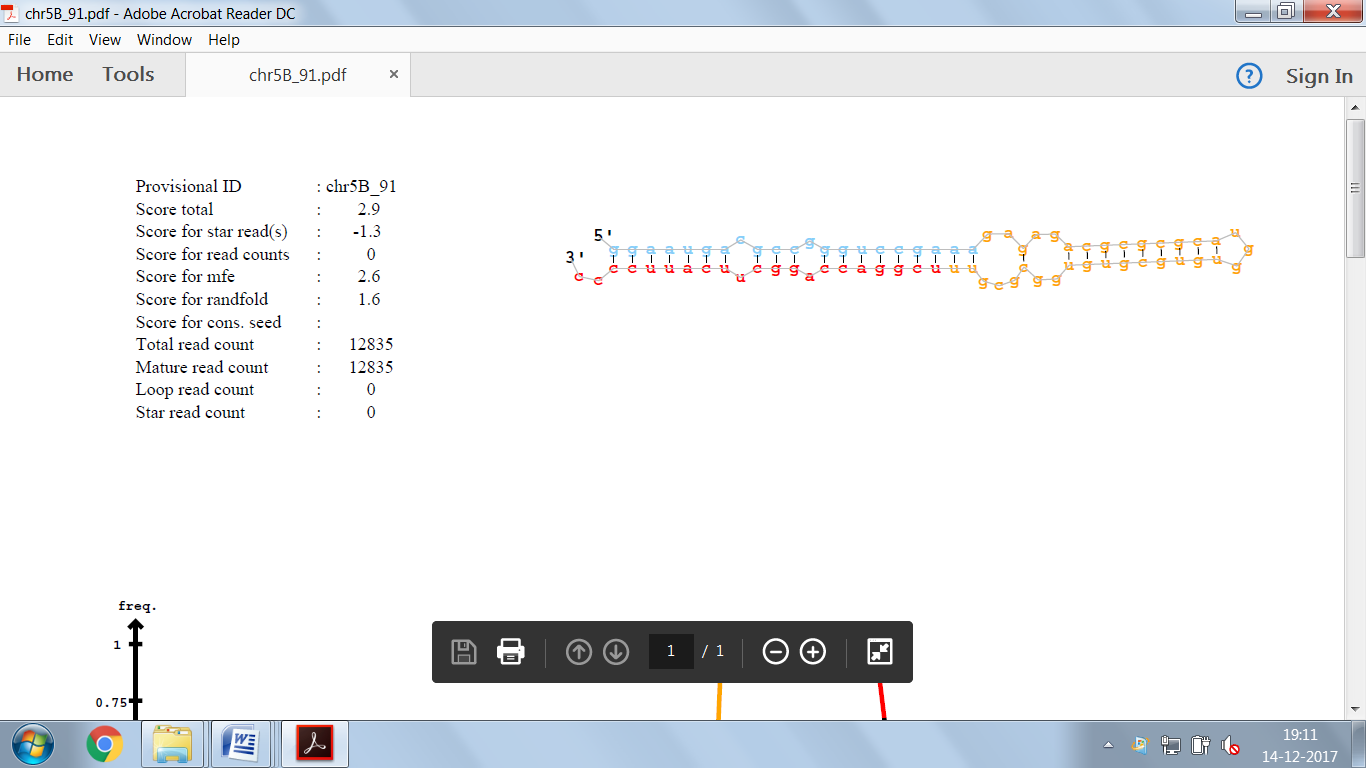


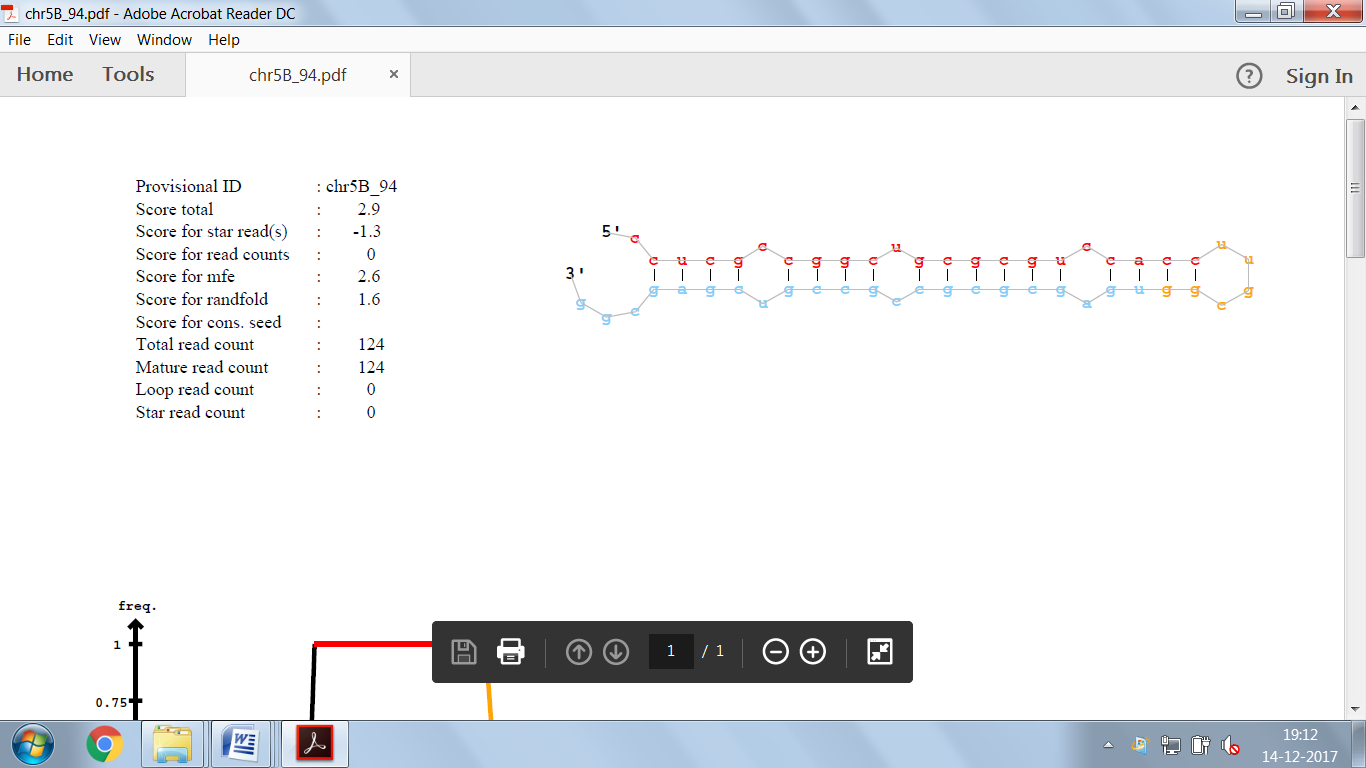


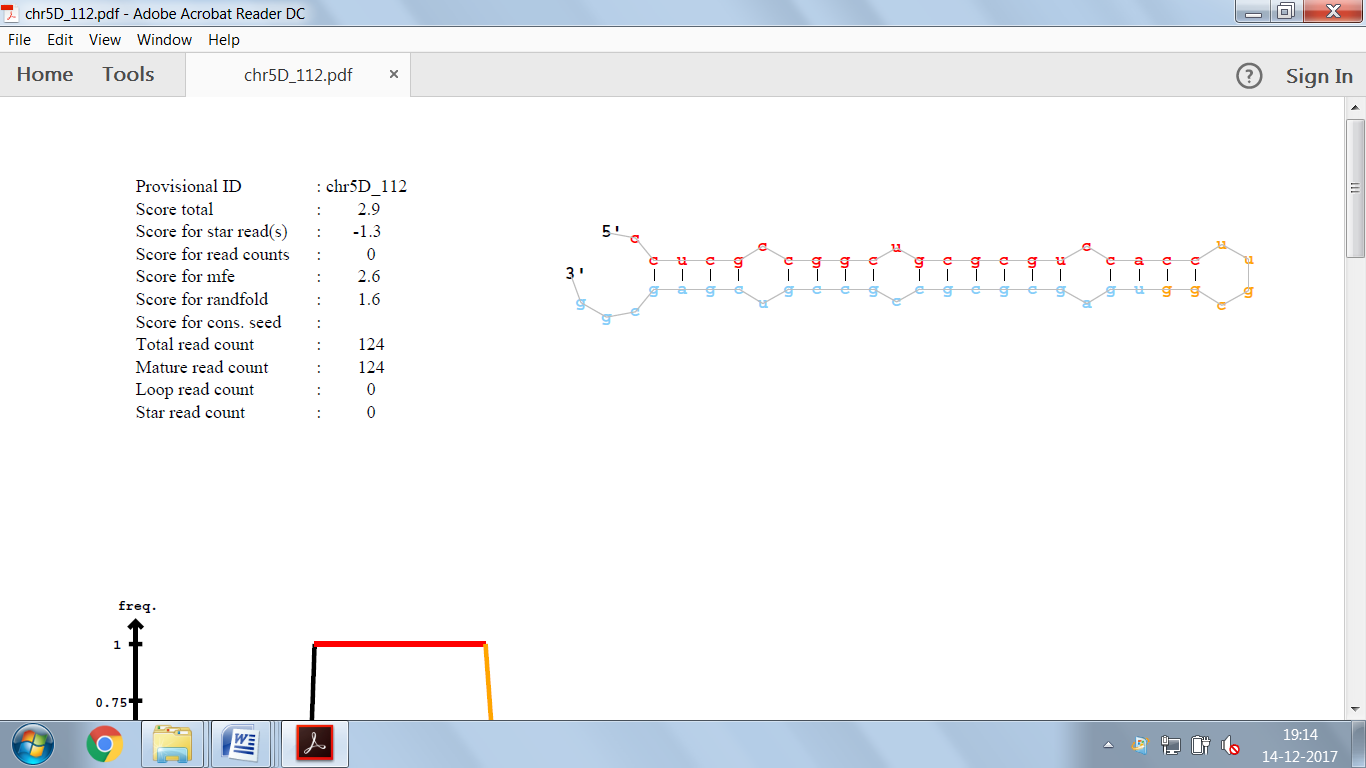


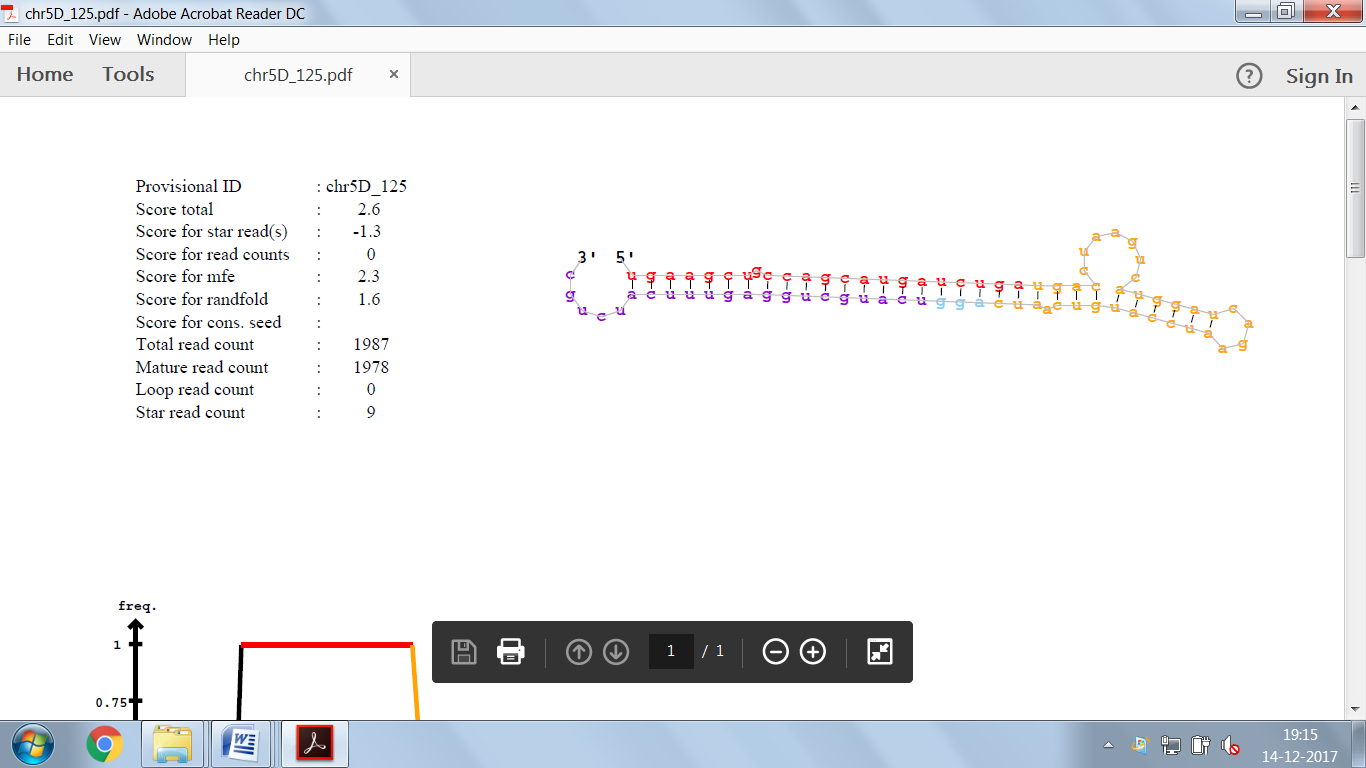


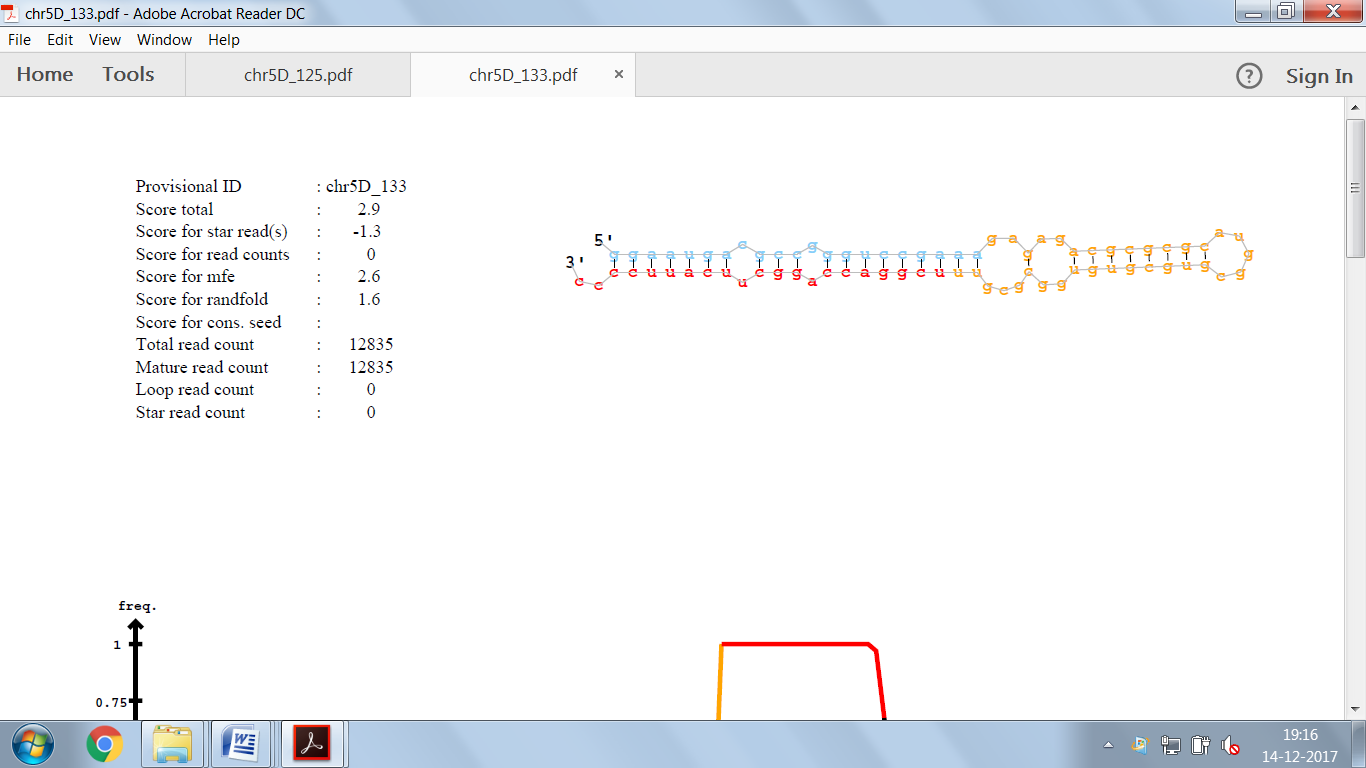


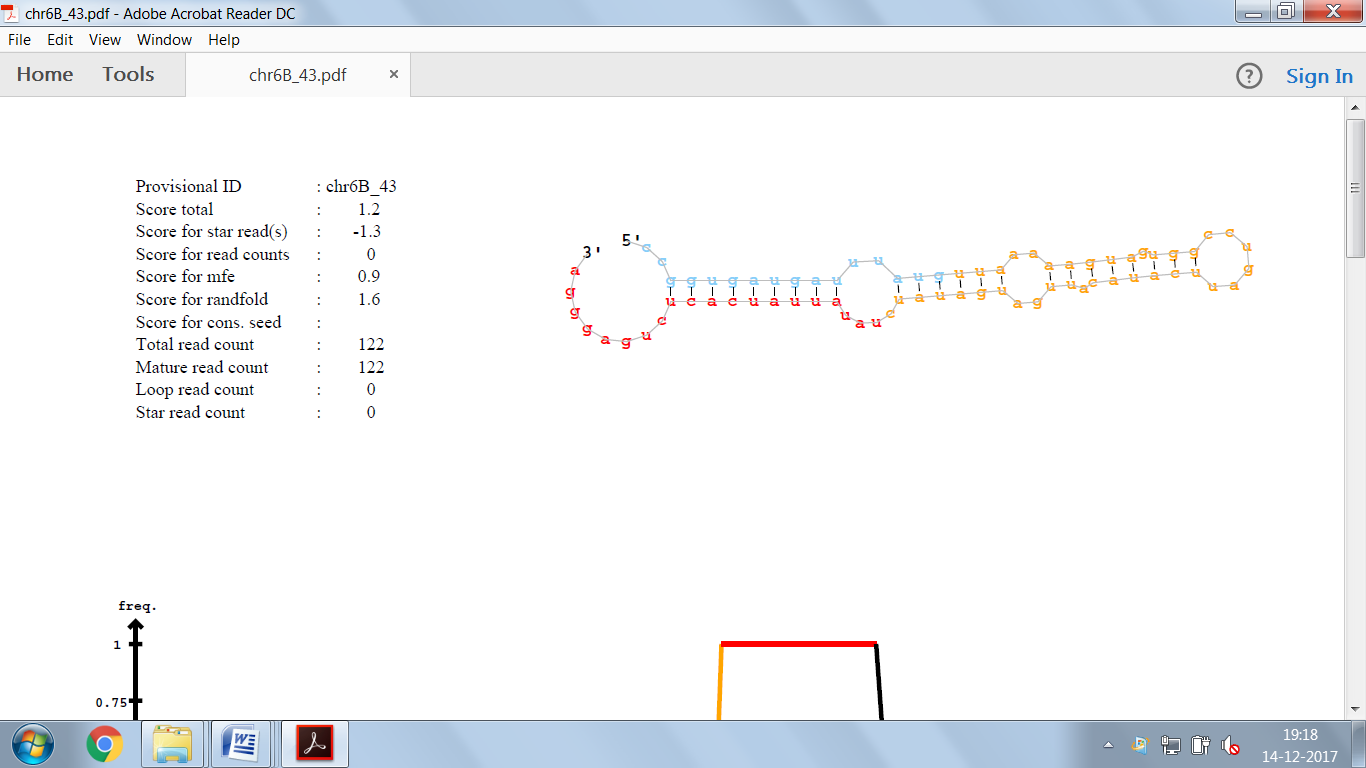


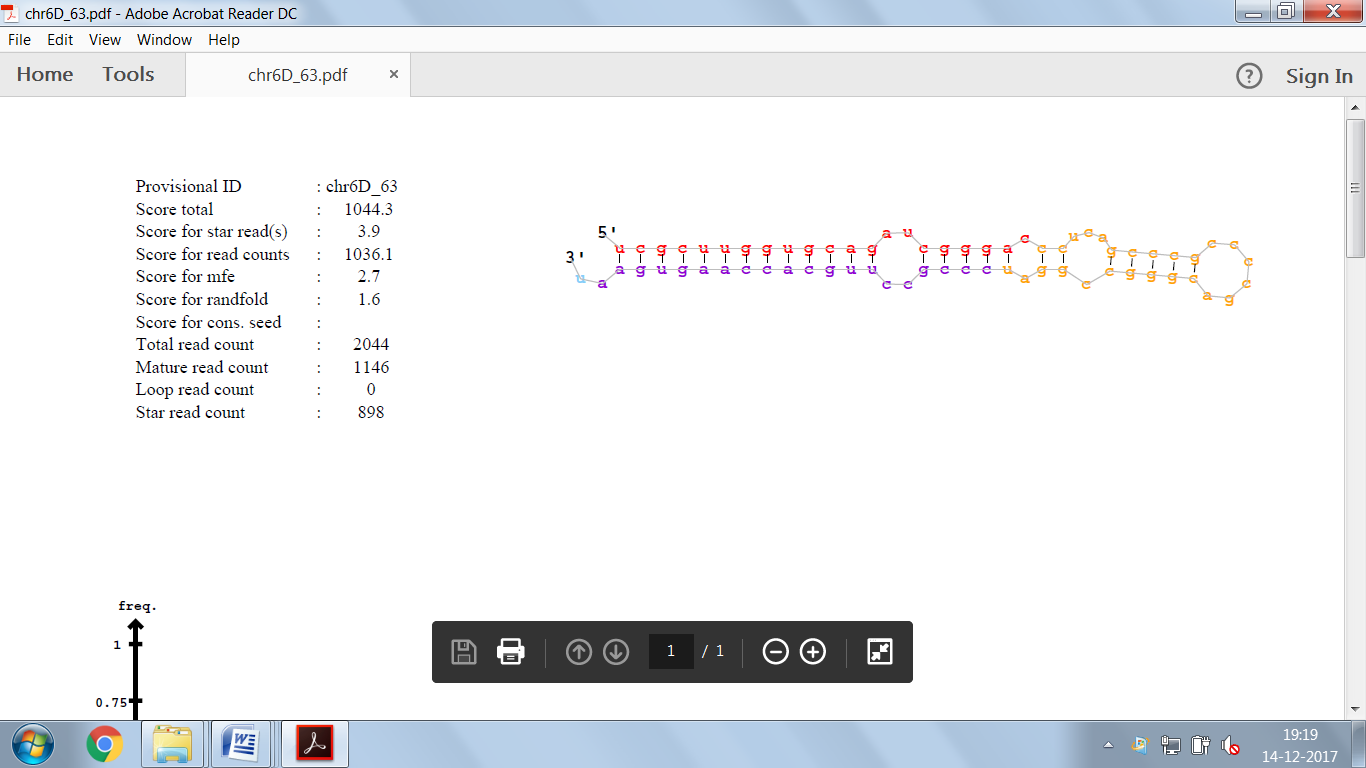


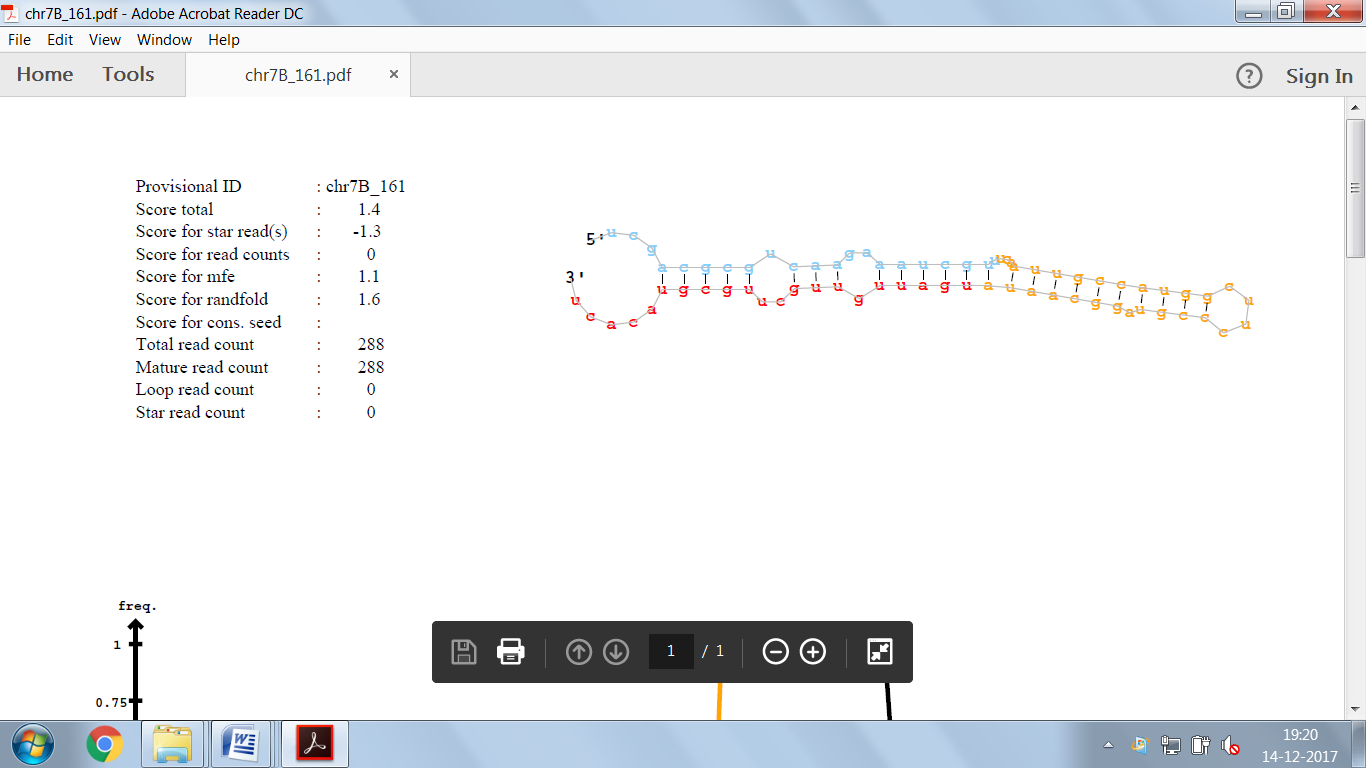


**Supp. Fig. 1c: Novel miRNAs structure identified in SC library.**


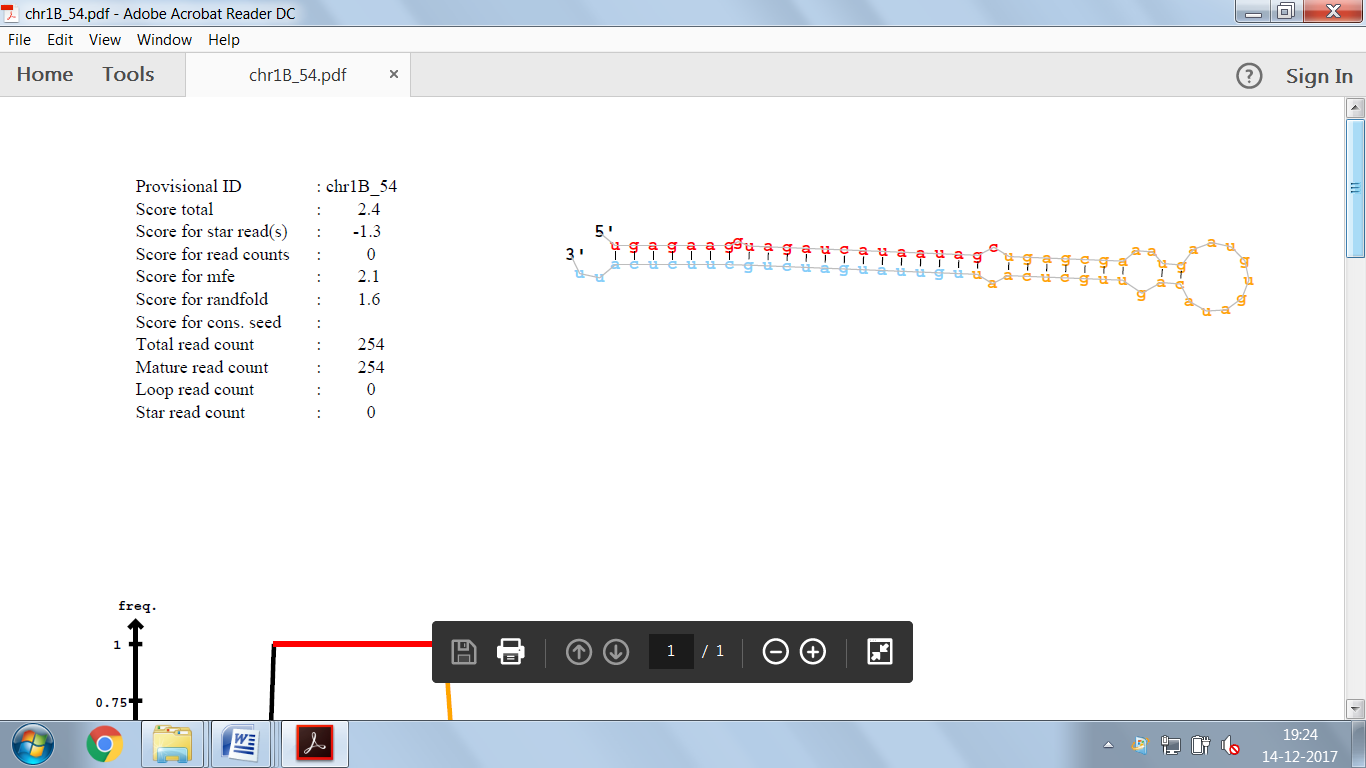


chr1B_54

chr1B_55

chr2A_121

chr2B_148

chr2B_157

chr2D_19

chr5B_89

chr5B_91

chr5D_123


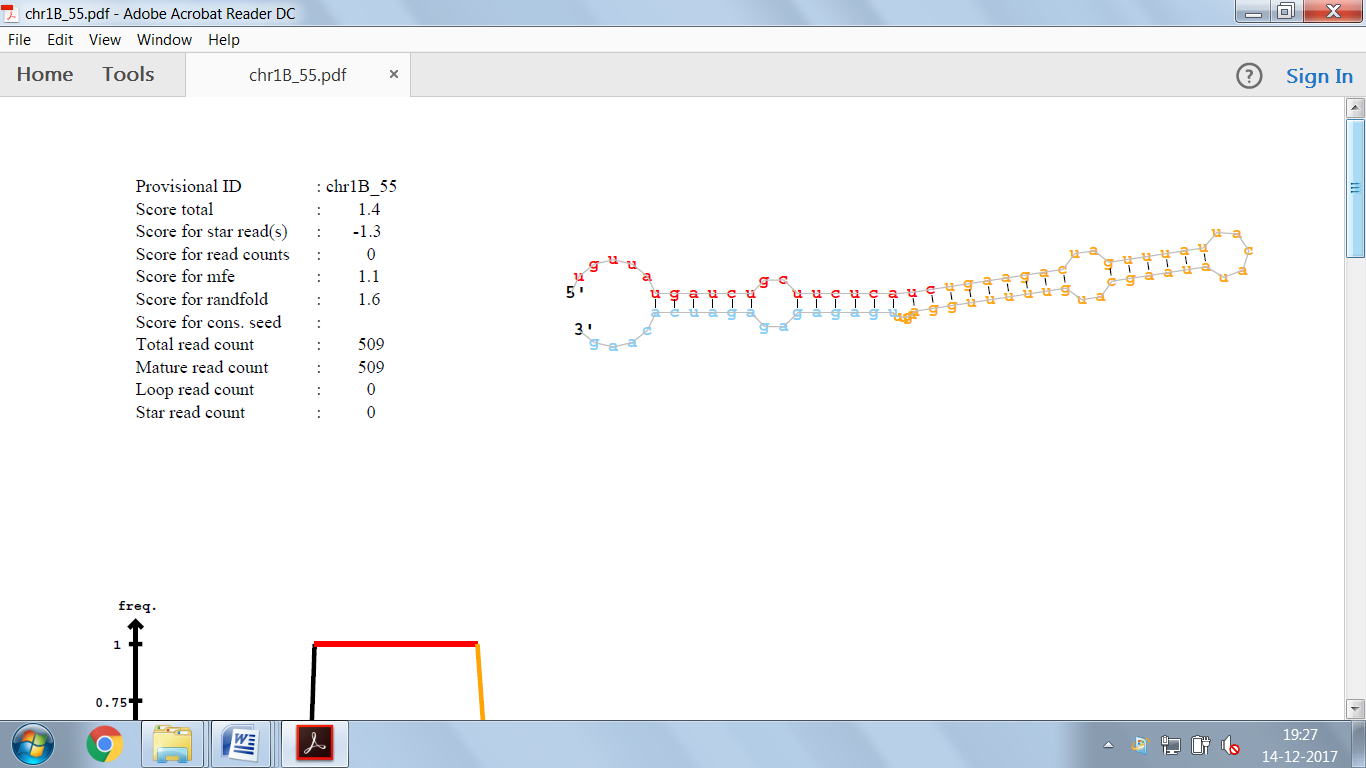


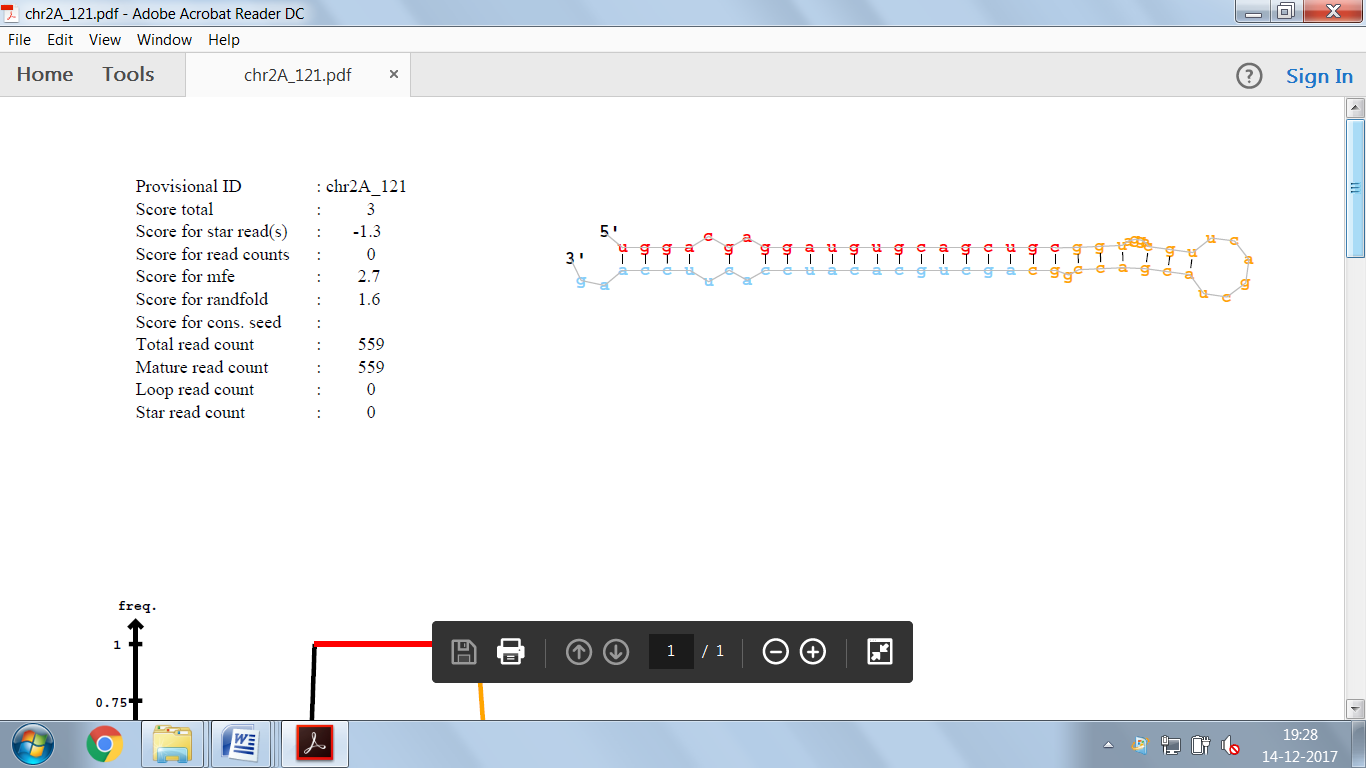


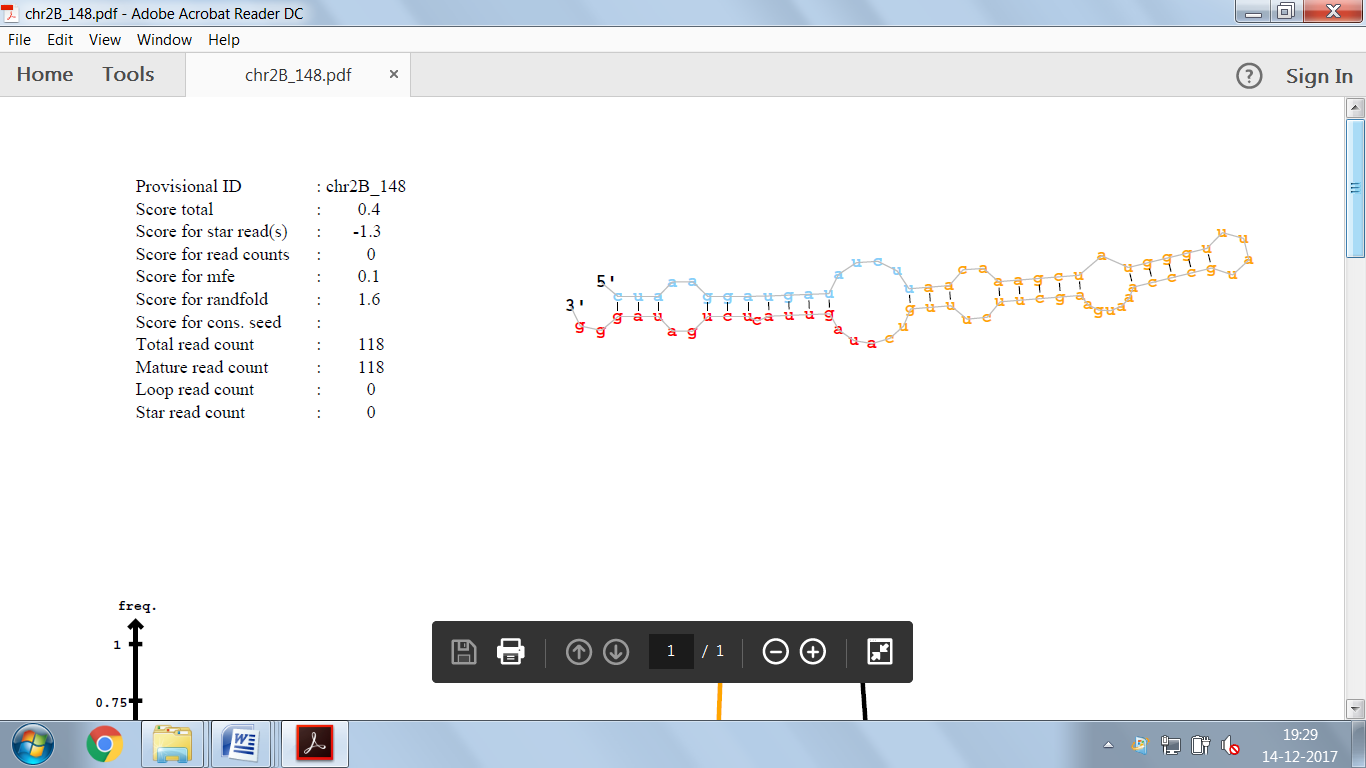


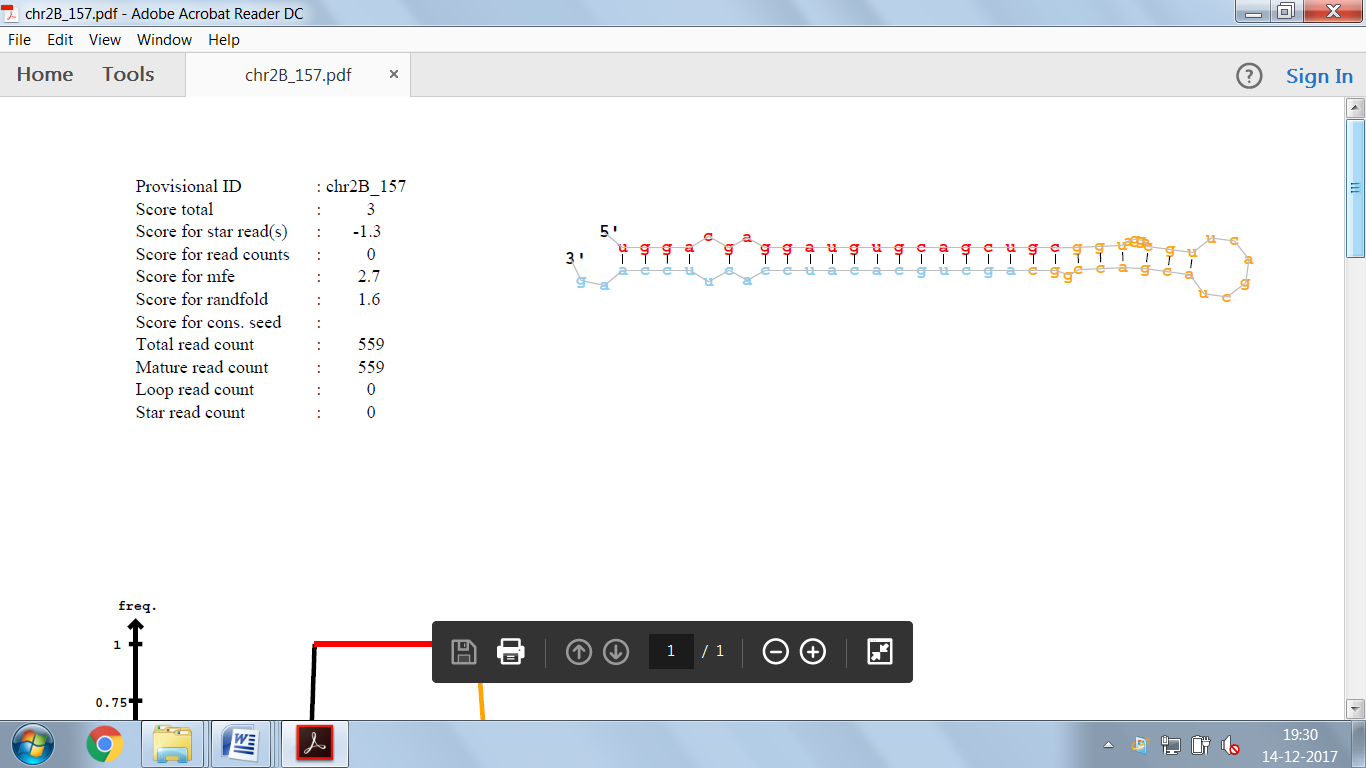


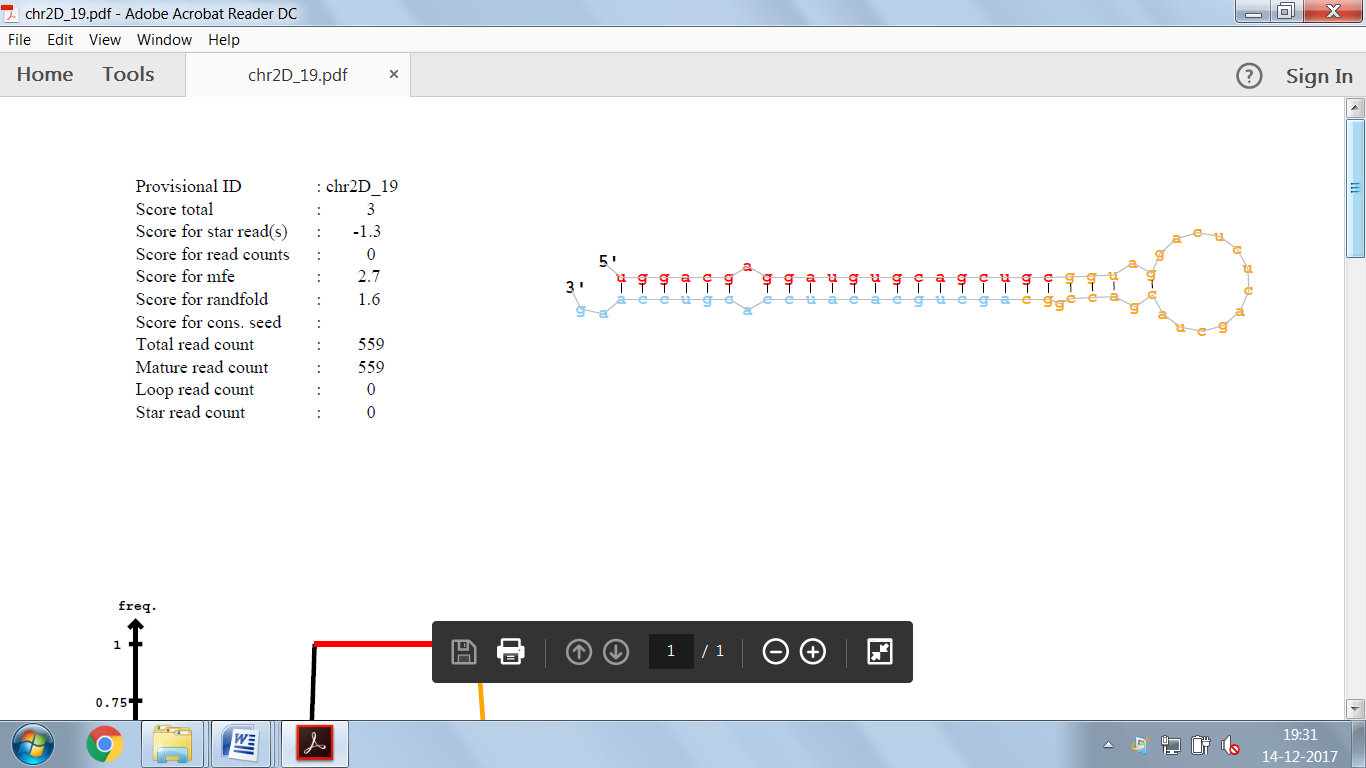


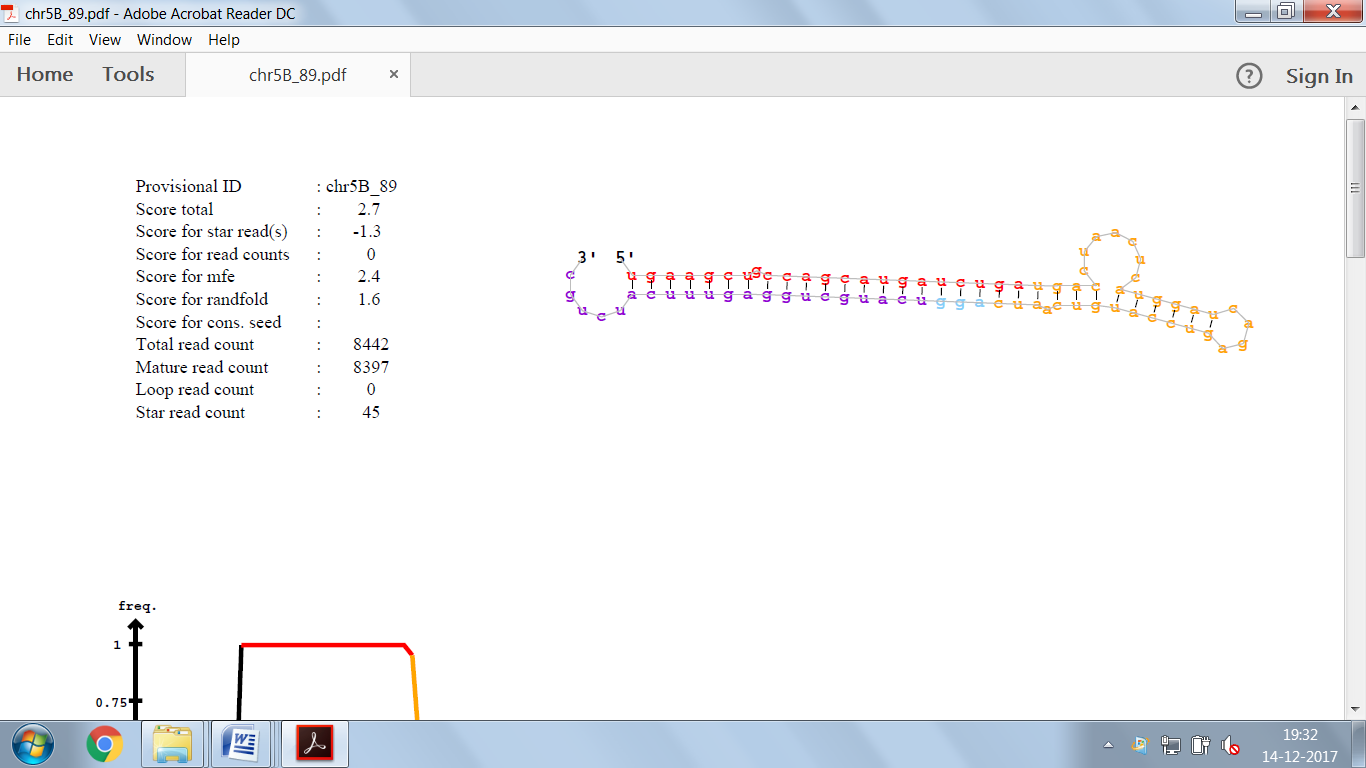


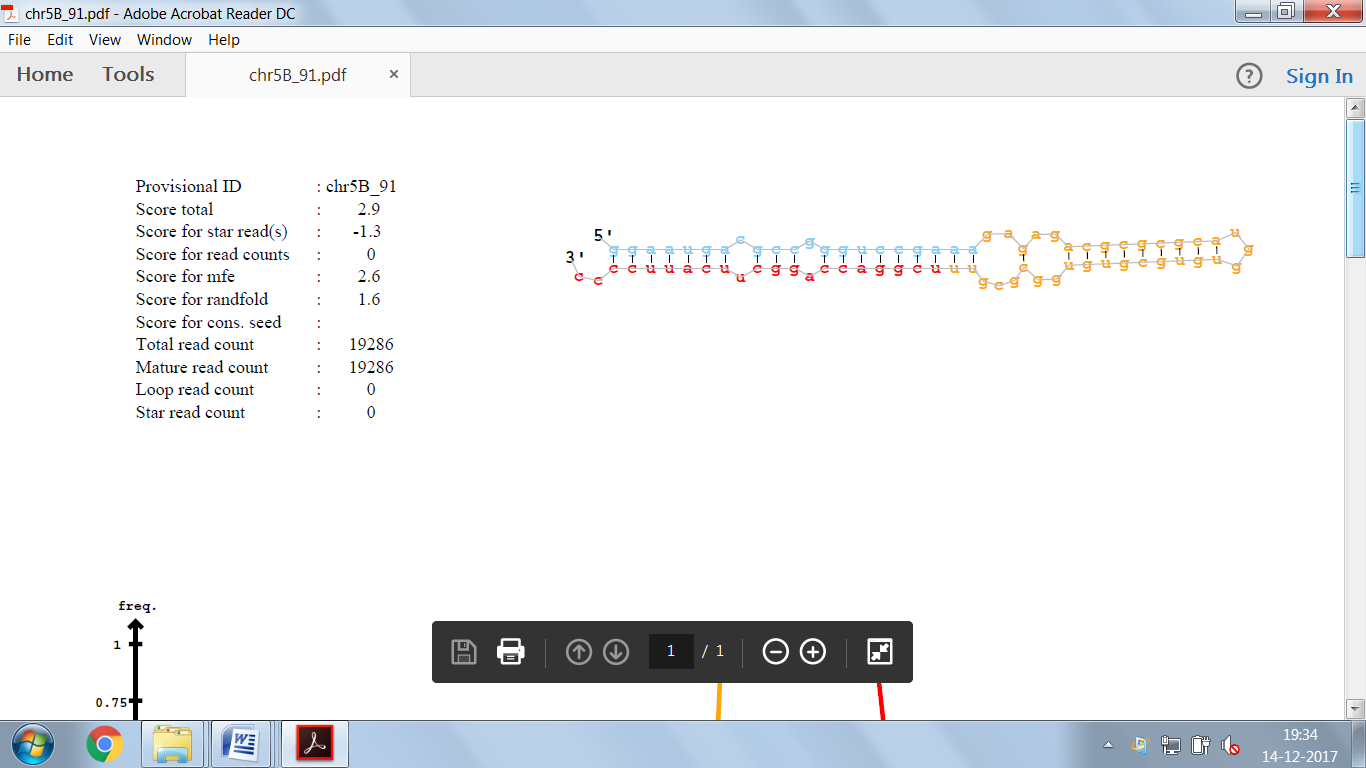


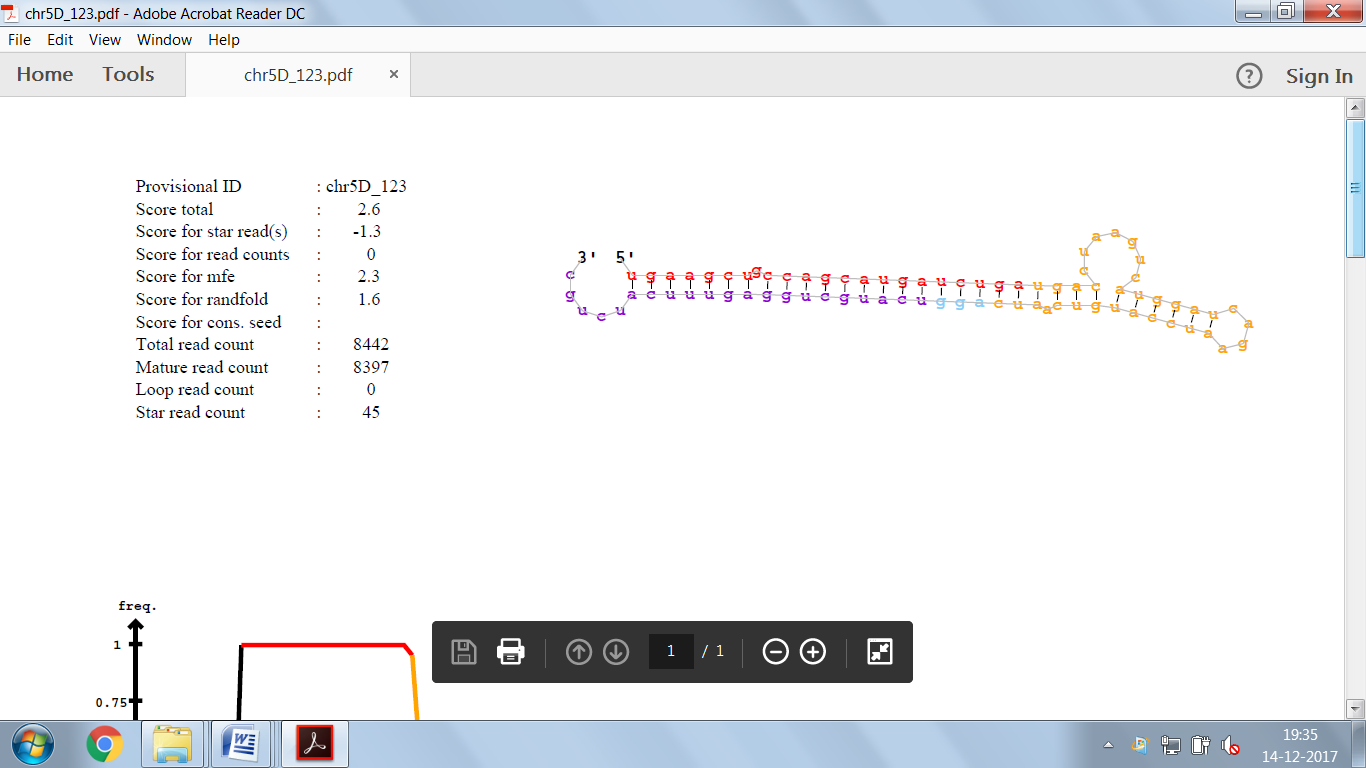


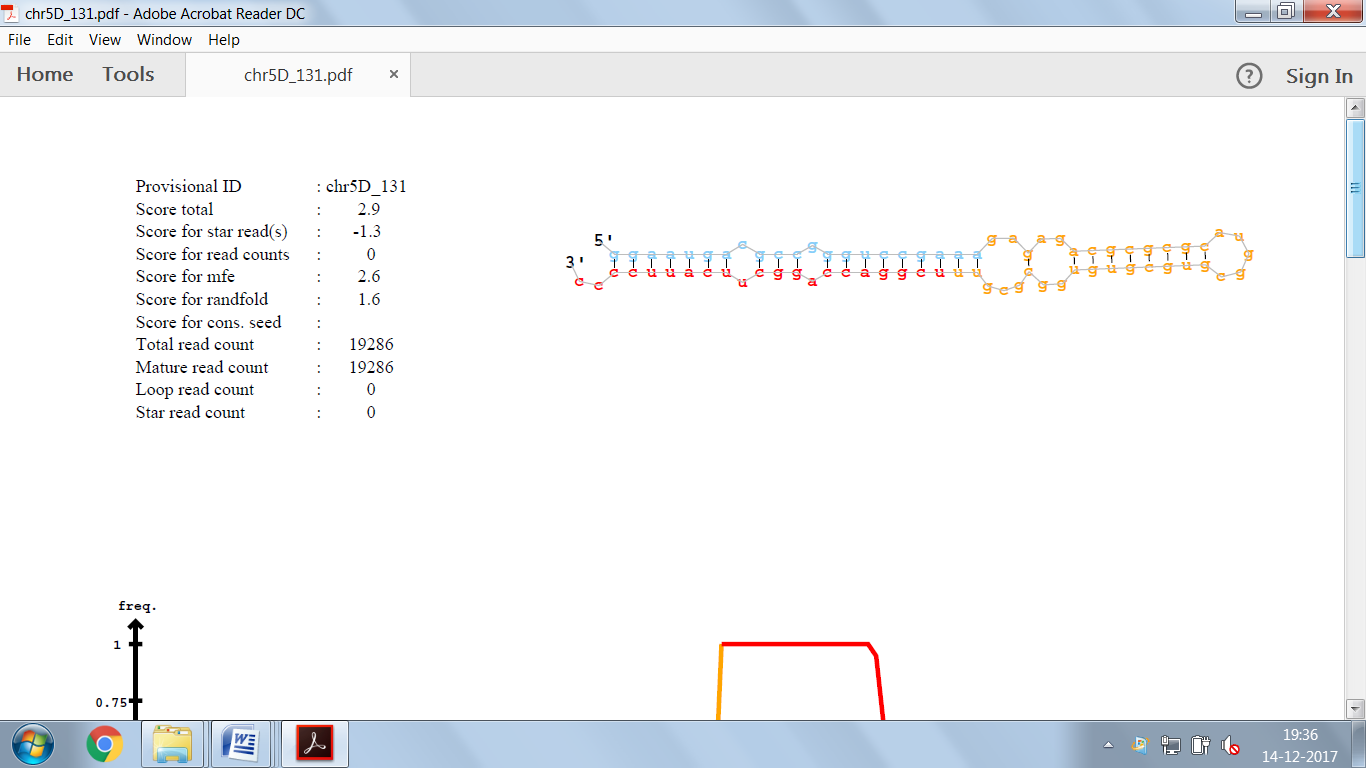


chr5D_131

chr6A_20

chr6B_25

chr6D_55

chr7D_168

chr7D_173


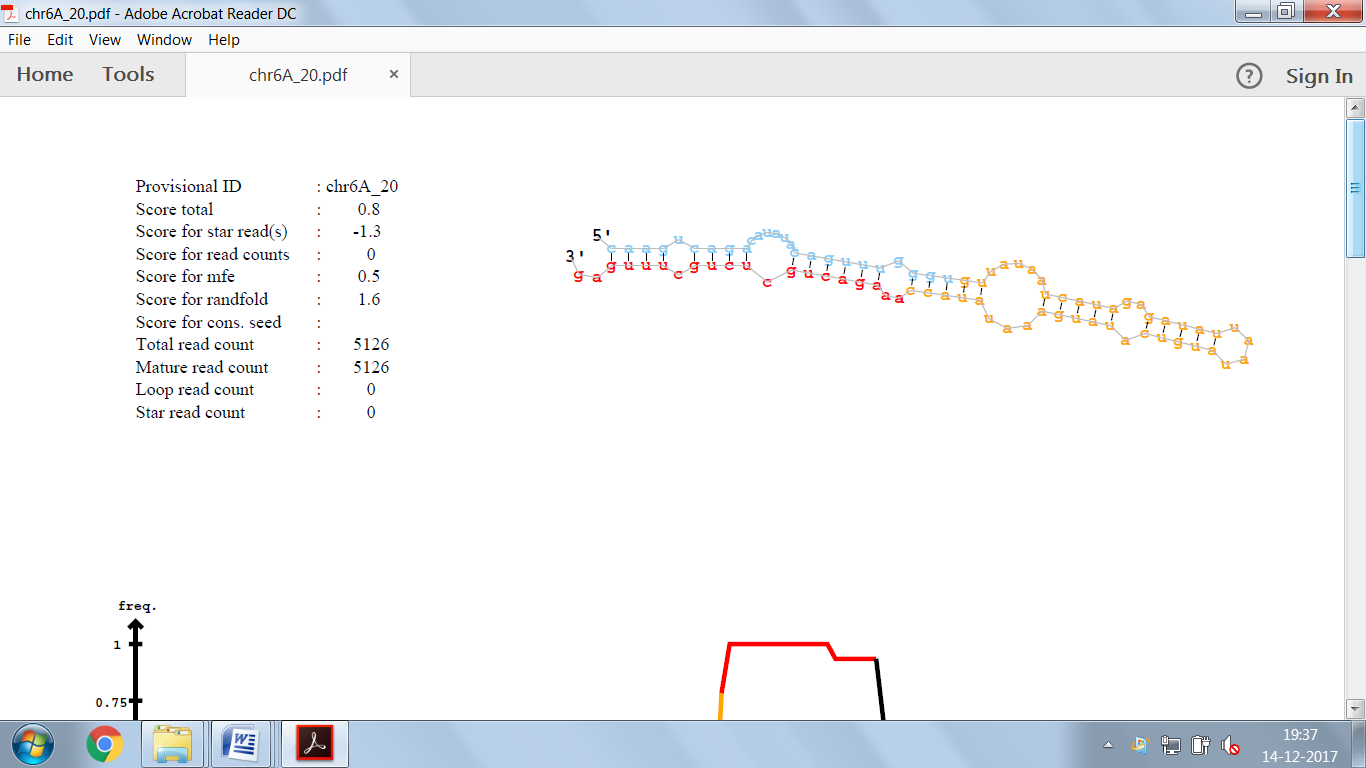


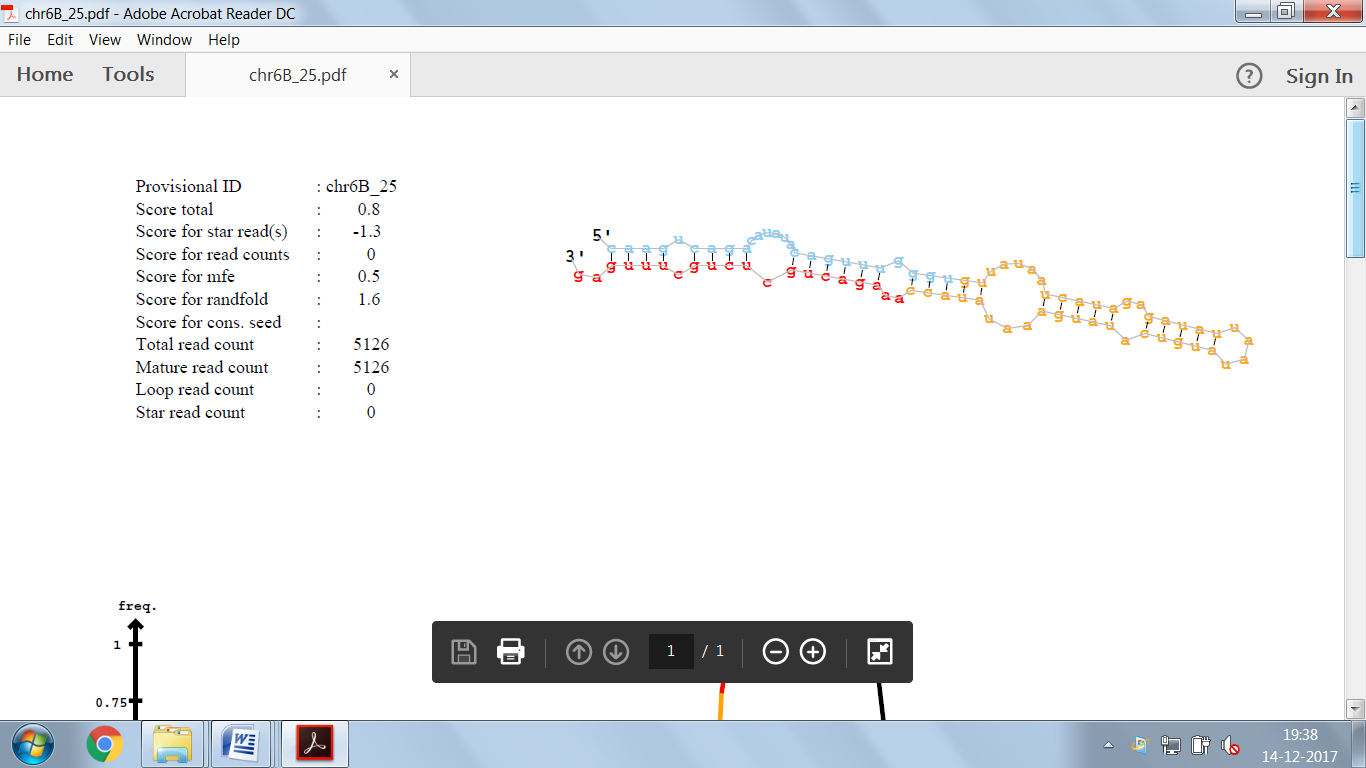


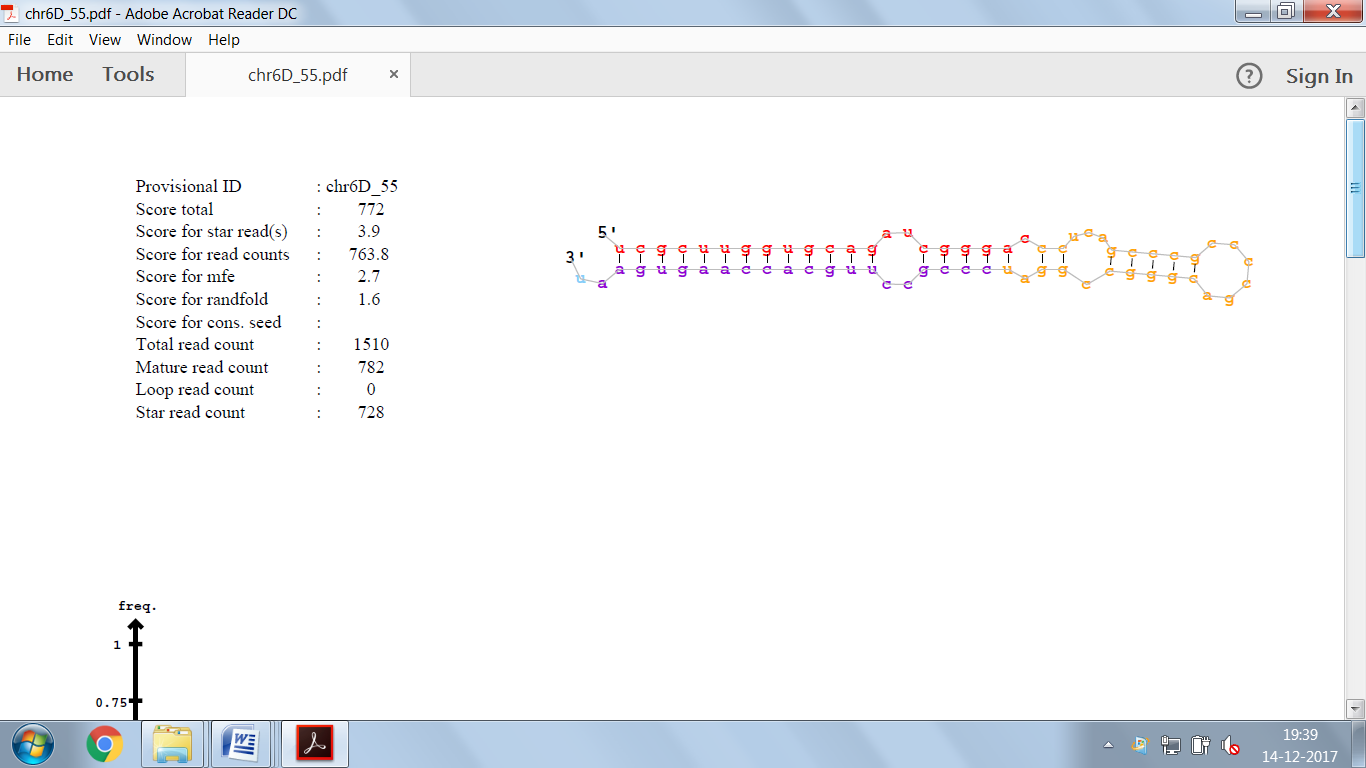


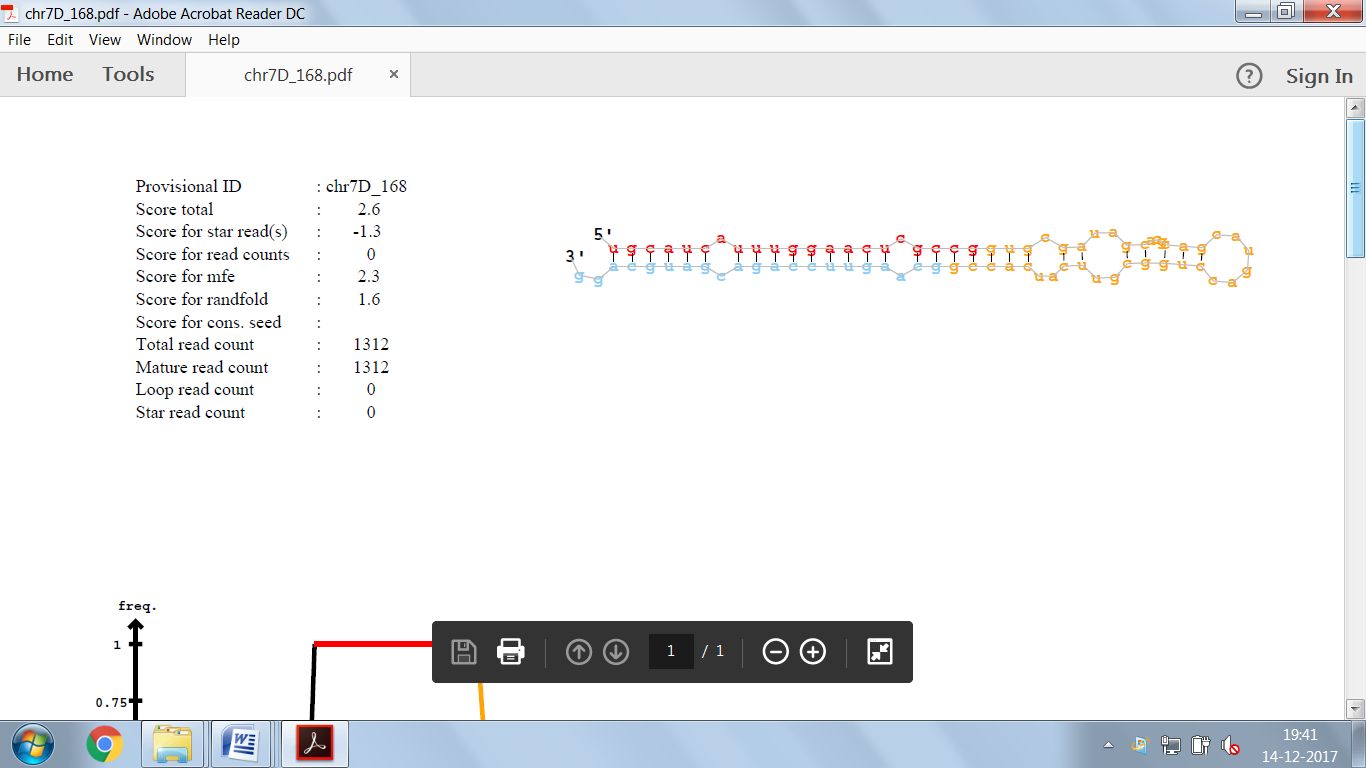


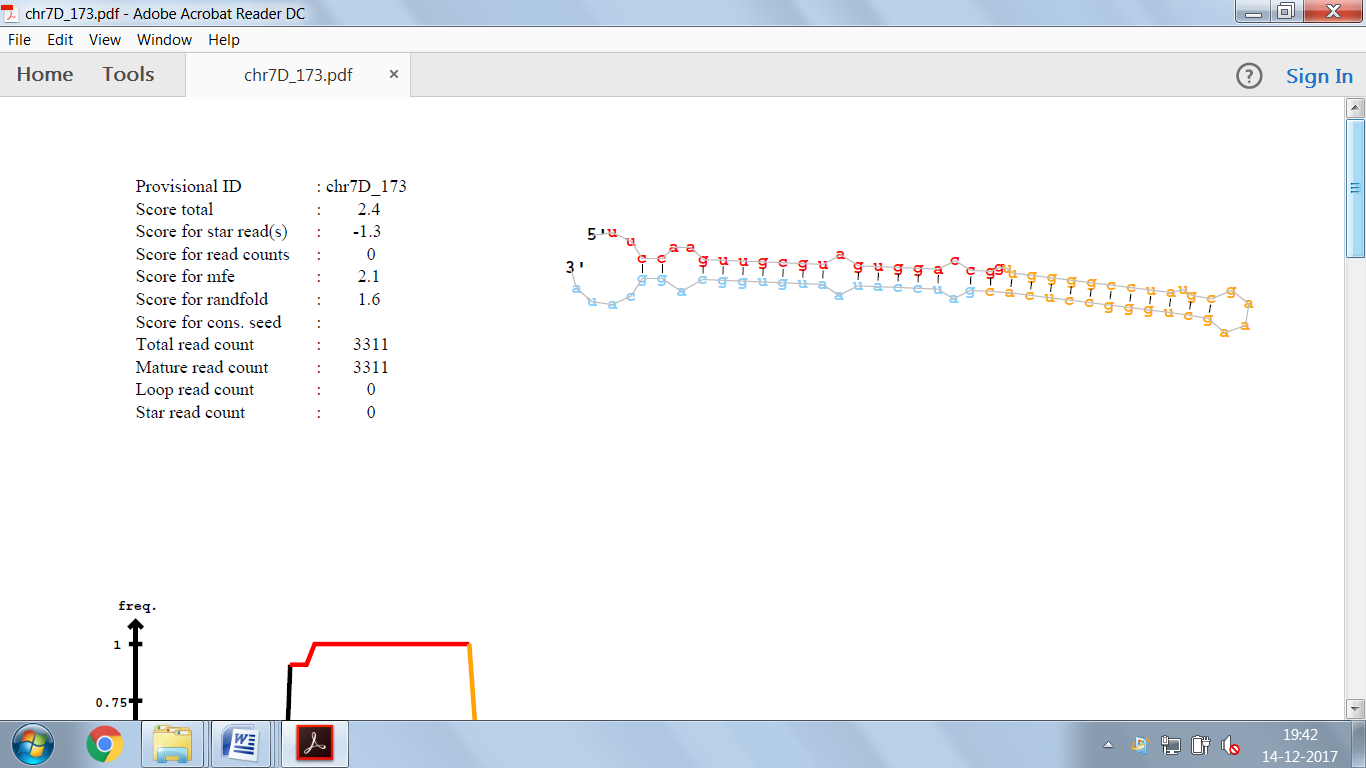


**Supp. Fig. 1d:** Novel miRNA structure identified in SD library. Here, the red and blue indicate the mature miRNA sequence and the complementary miRNA sequence (miRNA*), respectively. The purple color demonstrates the observed miRNA* sequence that differs from the expected miRNA* sequence. The yellow color sequence designates a hairpin loop of miRNA.


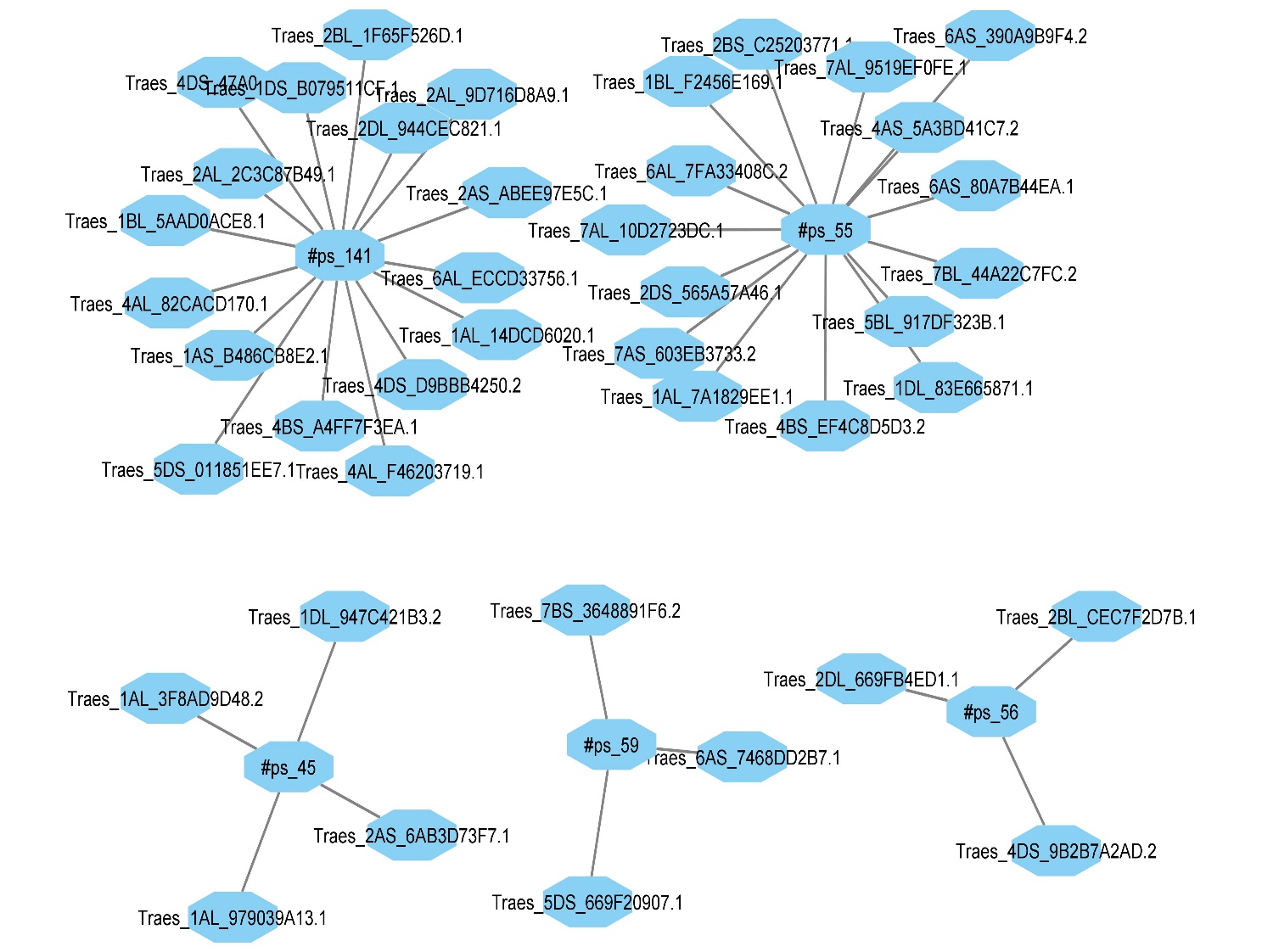


Supp. Fig. 2a: miRNA Network targeting wheat genes under drought stress in the TC library.


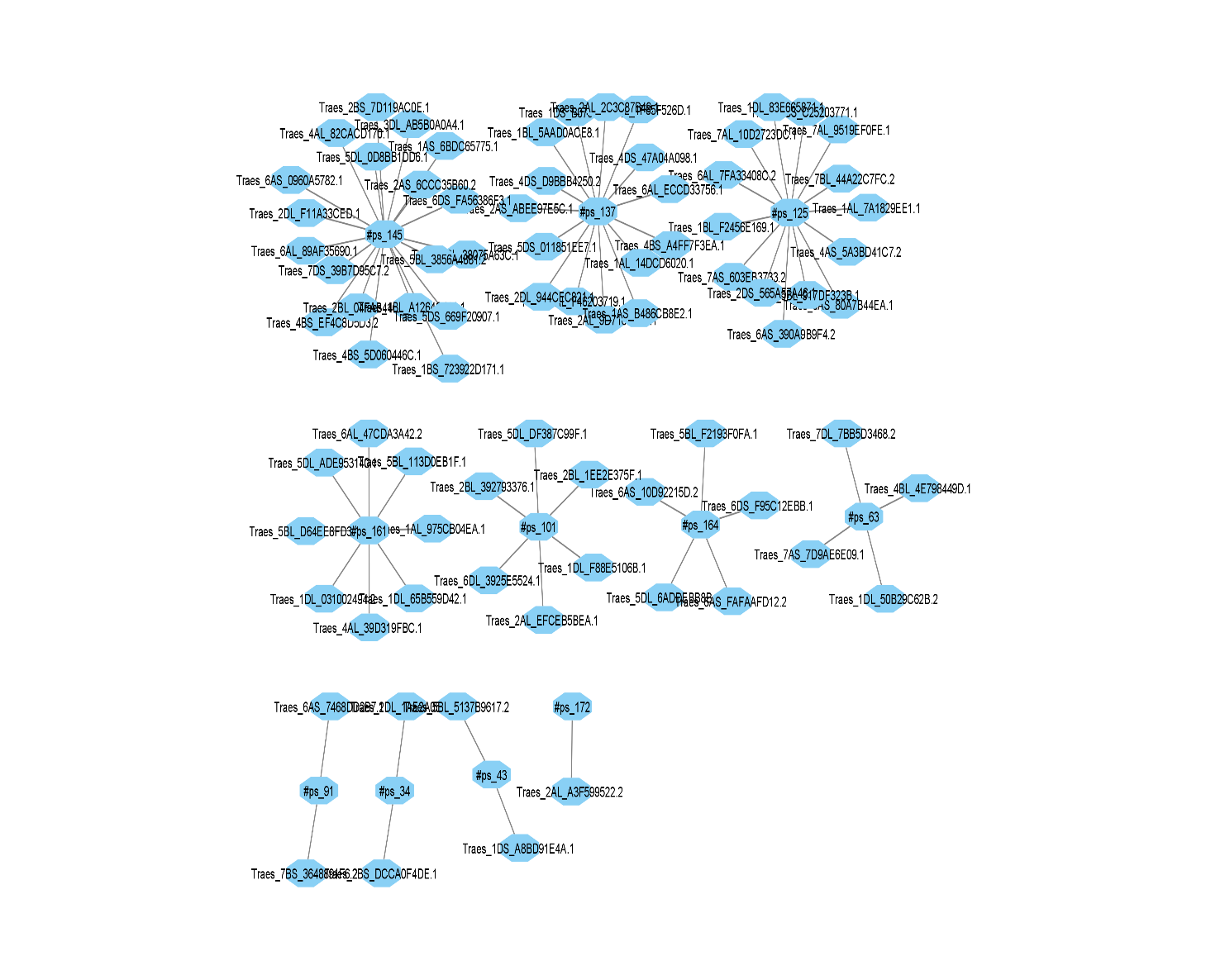


Supp.fig.2b: miRNA Network targeting wheat genes under drought stress in the SC library.


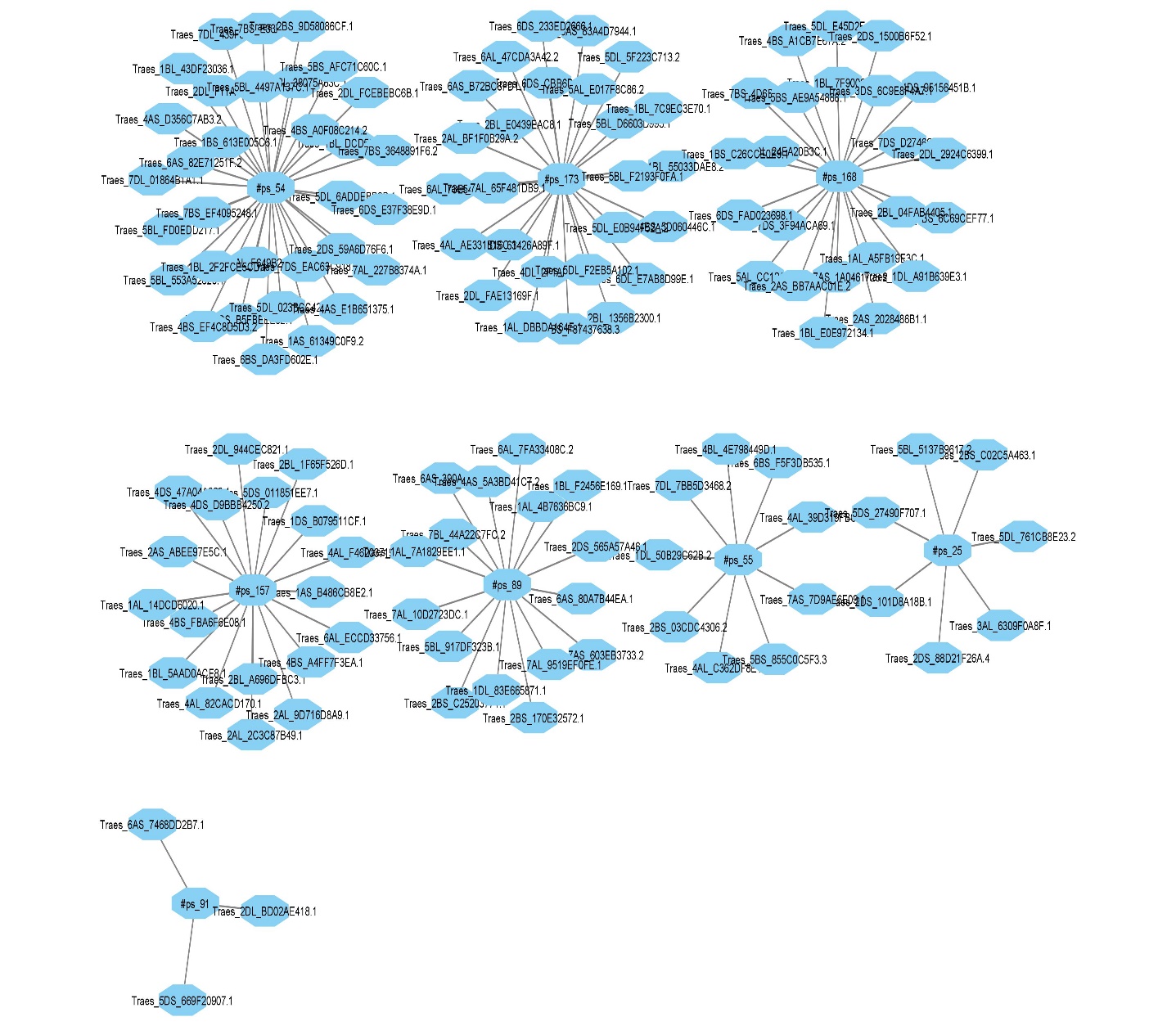


Supp. Fig. 2c: miRNA Network targeting wheat genes under drought stress in the SD library.
